# DUGMO: tool for the detection of unknown genetically modified organisms with high-throughput sequencing data for pure bacterial samples

**DOI:** 10.1186/s12859-020-03611-5

**Published:** 2020-07-06

**Authors:** Julie Hurel, Sophie Schbath, Stéphanie Bougeard, Mathieu Rolland, Mauro Petrillo, Fabrice Touzain

**Affiliations:** 1 ANSES, Laboratoire de Ploufragan, GVB unit, 22440 Ploufragan, France; 2 Université Paris-Saclay, INRAE, MaIAGE, 78350 Jouy-en-Josas, France; 3 ANSES, Laboratoire de Ploufragan, EPISABE unit, 22440 Ploufragan, France; 4 ANSES, Laboratoire de la santé des végétaux, 49000 Angers, France; 5 European Commission, Joint Research Centre (JRC), Ispra, Italy

**Keywords:** Detection, Unknown GMO, Bacteria, Illumina sequencing data, Machine learning

## Abstract

**Background:**

The European Community has adopted very restrictive policies regarding the dissemination and use of genetically modified organisms (GMOs). In fact, a maximum threshold of 0.9% of contaminating GMOs is tolerated for a “GMO-free” label. In recent years, imports of undescribed GMOs have been detected. Their sequences are not described and therefore not detectable by conventional approaches, such as PCR.

**Results:**

We developed DUGMO, a bioinformatics pipeline for the detection of genetically modified (GM) bacteria, including unknown GM bacteria, based on Illumina paired-end sequencing data. The method is currently focused on the detection of GM bacteria with – possibly partial – transgenes in pure bacterial samples. In the preliminary steps, coding sequences (CDSs) are aligned through two successive BLASTN against the host pangenome with relevant tuned parameters to discriminate CDSs belonging to the wild type genome (wgCDS) from potential GM coding sequences (pgmCDSs). Then, Bray-Curtis distances are calculated between the wgCDS and each pgmCDS, based on the difference of genomic vocabulary. Finally, two machine learning methods, namely the Random Forest and Generalized Linear Model, are carried out to target true GM CDS(s), based on six variables including Bray-Curtis distances and GC content. Tests carried out on a GM *Bacillus subtilis* showed 25 positive CDSs corresponding to the chloramphenicol resistance gene and CDSs of the inserted plasmids. On a wild type *B. subtilis*, no false positive sequences were detected.

**Conclusion:**

DUGMO detects exogenous CDS, truncated, fused or highly mutated wild CDSs in high-throughput sequencing data, and was shown to be efficient at detecting GM sequences, but it might also be employed for the identification of recent horizontal gene transfers.

## Background

A genetically modified organism (GMO) is a living being “in which the genetic material has been altered in a way that does not occur naturally by mating and/or natural recombination” (European Commission, 2001) [1]. We call the host genome the wild, original genome to which a genetic modification was made to build a GMO. In this article, we focus on GMOs resulting from the integration of exogenous genes into the host genome. European legislation requires the presence of GMOs in food products to be reported when they represent more than 0.9% of the ingredients [2]. This regulation requires that all GMOs or products sourced from GMOs must be declared to the European authorities, the modifications of their nucleic acid sequences have to be recorded in appropriate databases, and a detection method based on standard molecular techniques (e.g., PCR) must be provided. The GMOMETHODS [3], Eugenius [4], and GMO-Amplicon [5] databases list the GMOs authorized on the European market and their associated detection methods. In addition, Morisset et al. 2014 [6] developed a software program called GMOseek to optimise routine GMO testing in the laboratory, thus helping decision-making at all phases of analysis. More recently, with the development of high-throughput whole genome sequencing (WGS) [1, 7–10], bioinformatics analyses of the sequencing datasets based on alignment similarities [1, 7, 8] have been developed to characterize referenced GMO elements.

Willems et al. 2016 [11] proposed a statistical framework based on next generation sequencing (NGS) to predict the number of reads required to detect insert sequences, and thus confirm their integration into the host genome. All these tools facilitate detection work in routine laboratory testing, as long as the desired insert sequence is known. However, undescribed GMOs are also produced. For these, the sequence inserted into the host genome is completely unknown (not provided by GMO designers). Therefore, they are difficult to detect by the previously described methods. We must distinguish 3 types of unknown GM, those with only suppressed genes, those made by punctual mutations with CRISPR/TALEN technics, and finally those consisting of exogenous CDS insertion, CDS truncation or fusion with exogenous CDS. To detect this third, and most frequent, category of unknown GM, we propose a new bioinformatics tool called DUGMO, currently tailored to analyses of bacterial genomes.

Each species has its own vocabulary that can support statistical analyses. The most studied vocabulary property is codon usage [12], but longer words are used in statistical methods to decipher motifs with biological functions [13], to detect horizontal gene transfer [14], or to identify species-specific viral sequences [15] for instance. No application using these types of properties has been used in the context of GMO detection to date. DUGMO fills this gap.

DUGMO machine learning steps use two sets of CDSs. The first set corresponds to CDSs of the host genome with an extremely precise vocabulary definition. The second set corresponds to known GMO CDSs with a very wide range of vocabularies, only sketching possible diversity. To detect GM insert, DUGMO will report as GM all CDSs of the prediction set whose vocabulary differs from the one of the host genome.

DUGMO detects sequences of unknown exogenous inserts of a genetically modified (GM) bacterium. It uses high-throughput sequencing data that are previously cleaned and sorted thanks to a dedicated pipeline and two BLASTN alignments on the host pangenome (section 2.2). After the cleaning and sorting steps, a databank of known GMO inserts is cleaned to remove wild CDSs of the species (section 2.2). The proposed tool is based on statistical calculation of distances (section 2.3) associated with an automated learning step (section 2.4). GMOs resulting from small modifications by deletion or substitution will not be taken into account. GMOs resulting from methodologies such as CRISPR-CAS9 [16] can only be detected if they incorporate at least one exogenous CDS or involve gene truncation or fusion. Both chromosomal integration and plasmid insertion are detected.

## Results

### Datasets

A GM strain of *B. subtilis* has been sequenced at the Bavarian Health and Food Safety Authority (LGL) in Germany in the Hi-seq 1500 system [17, 18]. This dataset is called ‘Data1’ in the following. The paired Hi-seq 2500 sequencing data of the wild type *B. subtilis* 9407 strain used are available on the NCBI Sequence Read Archive (SRA) under accession number SRR8935610. This dataset is called ‘Data2’ in the following. The paired Mi-seq sequencing data of the three GM strains of *Escherichia coli* [19] used are available on the NCBI SRA under accession numbers SRR9304542, SRR9304539, and SRR9304540. These three genomes come from the same strain but each one has a distinct plasmid containing the *parE* gene from one of three different species: *Agrobacterium tumefaciens*, *Mycobacterium tuberculosis* and *Streptococcus pyogenes*. At the time of writing this article, the NGS datasets used are the only ones publicly available clearly described as GM bacteria. Two reference genomes, *B. subtilis* str. 168 (NC_000964.3) and *E. coli* str. K-12 substr. MG1655 (U00096.3) were also used. Two pangenomes were constructed, one for *B. subtilis* composed of 37 strains, and one for *E. coli* composed of 45 strains. Pangenomes were constructed manually to encompass the full range of analysed species and are available in the “testdata” directory of the DUGMO git repository. For *B. subtilis*, we took a known pangenome [20] and enriched it with wild strains from various environments available on the NCBI (list of accession numbers given in the Additional file 1, section 2). For *E. coli*, the 5 main species subgroups [21] were considered and enriched with newer complete PacBio genomes and plasmids from various environments available on the NCBI (list of accession numbers given in the Additional file 1, section 3). A databank of GMO coding sequences (CDSs) provided by the JRC and supplemented by insert CDSs of bacterial GMOs from the literature was created (list specified in Additional file 2, related literature references given in Additional file 3). This databank currently does not include GM CDSs of bacteria analysed in this paper. DUGMO requires paired Illumina sequencing data of a single bacterial species; it does not deal with metagenomic or multigenomic data. The JRC sequences included in the GM databank provided in this paper are obtained by PCR simulation screening of public nucleotide sequence databanks including patents and available whole plant genomes [22].

### Cleaning pipeline for raw data, host pangenome and known GMO databank

**Fig. 1:**
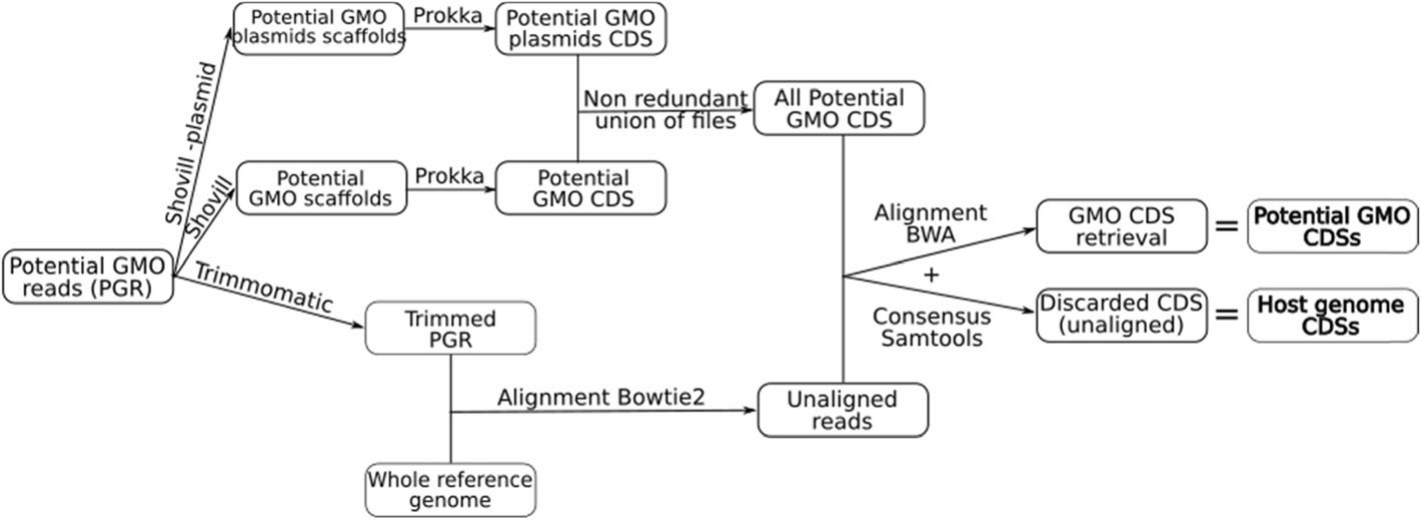
Diagram of cleaning pipeline for raw data allowing preliminary sorting of the CDSs of the potential GM genome

The objective of this section is to describe the production of three sets of data: host genome CDSs, potential GM CDSs, and known GMO CDSs. To create these datasets, a pipeline was implemented to clean the raw data of the suspected GM bacterium by eliminating all sequences belonging to the reference genome. This step is frequently used in the processing of high-throughput sequencing data (e.g., refer to Baron et al. 2018 [23] to remove chromosomal reads to assemble *E. coli* plasmids). This pipeline sorts CDSs into two categories: CDSs of potential GMO inserts and CDSs belonging to the wild type genome. Several steps are required (Fig. 1). First, the raw sequencing data of the potential GM bacterium are assembled twice with Shovill 1.0.4 [24] with the trim option, once on the whole genome, and once focusing on plasmids (plasmid option of SPAdes 3.13.1 [25]). Scaffolds with a coverage depth of less than 3 are eliminated; this threshold is intended to remove potential contamination of the bacterial genome, such as contamination by another sequenced library or by the matrix in which the bacteria are located. These two results of assembly are annotated with Prokka 1.12 [26] with the use-genus option to predict CDSs. These CDSs are merged and filtered out for duplicated sequences. In parallel, the raw sequencing data of the potential GM bacterium are “trimmed” using Trimmomatic software 0.39 [27], and aligned by Bowtie2 2.3.4.3 [28] with the reference genome. Then, the non-aligned reads (including any GMO inserts) are aligned with the CDSs predicted in the previous Prokka CDSs using BWA MEM 0.7.17 [29]. A consensus of the covered CDSs is deduced from the BWA aligned reads using Samtools 1.9 [30]. At the end of these steps, the CDSs present in this BWA alignment consensus and containing the potential GM insert sequences are retained as potential GM CDSs. The remaining CDSs are kept as CDSs related to the host genome.

A pangenome groups together the full range of genes present in the same species. Two BLASTN 2.6.0 [31] alignments of the potential GM CDSs generated by the cleaning pipeline are performed on the pangenome of the potentially GM bacterium, one on CDSs and one on whole genomes (Additional file 1, Fig. S1). The aim of these BLASTN alignments is to carry out secondary sorting (the first was done by the cleaning pipeline) in order to separate the CDSs related to the host genome from potential GM CDSs. The first BLASTN on pangenome CDSs removes CDSs belonging to the host genome that are present in potential GM inserts (for parameters see Additional file 1, section 4.1). The second BLASTN on the pangenome aims to remove CDSs predicted by Prokka, but corresponding in our pangenome to non-coding sequences (for parameters see Additional file 1, section 4.2). The CDSs aligned at least in one of the two BLASTN steps are added to the CDSs related to the host genome defined at the end of the cleaning pipeline. The non-aligned CDSs are kept as potential GM CDSs.

After these two sorting steps, the databank of known GMO inserts is filtered to define a set of known GM CDSs used for distance calculation (section 2.3). A CDS is qualified as GM only when considering it in a setting different from the genome in which it is naturally present. When the aim is to identify whether a bacterium or an organism is a GMO or not, all CDSs naturally present in the genome of the species of the suspected strain are eliminated from our databank of GMO CDSs using a BLASTN alignment (for parameters see Additional file 1, section 4.3). In addition, the parts of CDSs aligned during this BLASTN are also removed from the CDSs in the databank, leading to a filtered bank of GMO CDSs. We computed median for each of the three distances (P L3M1, P L4M2 and F L9M7 in section 2.3) of each host genome CDS and the set of all host genome CDSs and called them respectively medL3M1, medL4M2 and medL9M7. Finally, CDSs of known GMO databank whose distances to the set of all host genome CDSs verify (P L3M1 < medL3M1 and P L4M2 < medL4M2 and F L9M7 < medL9M7) are discarded.

After the two sorting steps and filtering of the databank, we then produced three disjoint sets of CDSs in which each CDS is individually compared to the set of all host genome CDSs: (a) the filtered bank of GMO CDSs, (b) the host genome CDSs, and (c) the potential GM CDSs.

### Vocabulary of the host genome and bray-Curtis (BC) distances

#### General information

To assess whether or not each potentially GM CDS is distant from the CDSs of the host genome, a distance calculation is proposed with the idea that the greater the distance, the higher the probability that a CDS will be part of a GM insert. Our hypothesis is that the insert CDS introduced into the unknown GMO should have a different “vocabulary” from that of the genome at the source of the GMO. In fact, a genome has its own vocabulary, made up of words. A word is a short sequence of nucleotides with a predefined length, such as “ATGCCT”. The vocabulary, denoted by *M*, that is used in our method can be either the set of all words of a given length *l* or a subset of these words, specific to the host genome. In the second case, the word selection is made initially by using R’MES software [32] that searches for exceptional words in a given sequence, i.e. significantly over- or under-represented words with respect to a given Markov model. For each word of a given length, R’MES computes a score of exceptionality that measures the significance of the difference between its observed and expected number of occurrences. For this, the distribution of the number of occurrences is approximated by Gaussian distribution, well adapted for frequent words.

#### Bray-Curtis (BC) distances

After comparing several alternative distances (Additional file 1, section 5), the most relevant Bray-Curtis dissimilarity was selected. This dissimilarity index does not verify the triangle inequality so it should not be called a distance. However, to facilitate understanding, the term “Bray-Curtis distance” will be used in this article. The Bray-Curtis distance is initially an ecological distance [33] to evaluate the dissimilarity between two given samples in terms of the abundance of available species. This distance is equal to 1 when the two compared datasets have no common point, and 0 when they have identical compositions. Our aim is to compare a sequence *S* and a set of sequences *H* based on their word compositions, via the following equation

1 $$ BC\left(S,H\right)=1-\frac{2{\sum}_{m\in M}\left[f\left(m,S\right)+f\left(m,H\right)\right]}{\sum_{m\in M}\left[f\left(m,S\right)+f\left(m,H\right)\right]} $$

where *M* is the set of the selected words of length *l* (in case of no selection, *M* is the set of all the *4*^*l*^ possible words of length *l* in the alphabet {A,C,G,T}), *m is* one word in *M*, *f* is a function set either to *F* (for frequencies; defined in the following Eq. (2)) or *P* (for proportions; defined in the following Eq. (3)), and relates to the count of motif *m* in the ad-hoc sequence. In practice, *S* is typically a CDS (potential GM or not) and *H* is the set of deduced host CDSs, leading to a notable difference in the cumulated length of these two sequence sets, and then in the order of magnitude of the counts in *S* and *H*. To circumvent this problem, the Bray-Curtis distance is computed on normalized counts, and two normalizations are considered, called in frequencies versus in proportions, as explained below.

#### BC distance in frequencies

The *f* function in Eq. (1) is set to

2 $$ f\left(m,S\right)=F\left(m,S\right):= \frac{C\left(m,S\right)}{\sum_{w\in M}C\left(w,S\right)} $$

where *C*(*m*,*S*) denotes the number of occurrences – or count – of word *m* in sequence *S*, and *w* is every word in *M*. In other words, f(m,S) is the normalized frequency of m in S. For this calculation of Bray-Curtis distance, only the nucleotides at the third position of the codons of the CDSs used are considered, denoted by CDS_3_. The justification for the concatenation of the third position of the codons is given in the Additional file 1, section 6. The CDS_3_ set allows us to consider words of size *n* containing the useful information of words of size 3*n* in the entire CDSs.

#### BC distance in proportions

The *f* function in Eq. (1) is set to

3 $$ f\left(m,S\right)={P}_H\left(m,S\right):= C\left(m,S\right)\times \frac{\sum_{w\in M}C\left(w,H\right)}{\sum_{w\in M}C\left(w,S\right)}. $$

In other words, *f(m, S)* is a normalization of the count of *m* in *S* equivalent to consider *m* in a sequence as large as the cumulated length of *H*. Note that with this definition, when *S = H*, we get *f(m, H) = C(m, H)*.

#### Preliminary results

In an initial step, the BC distances are computed both in frequencies and in proportions, with words of length *l* from 3 to 9, maximum Markov models of order *k = l-2* and different ratios of selected words. These combinations of parameters are tested on our datasets Data1 and Data2, in which the GM bacterium status is known (section 2.1) to better separate the host CDSs from the GM CDSs in terms of distance. Three different combinations are chosen to be used jointly in the machine learning step described below:

- (P L3M1): BC in proportions for all 3-letter words under Markov model of order 1.
- (P L4M2): BC in proportions for all 4-letter words under Markov model of order 2.
- (F L9M7): BC in frequencies for the 10% most over-represented 9-letter words under Markov model of order 7.

P L3M1 was chosen because this calculation characterizes the three-letter words of the CDSs, which is specific to the host genome vocabulary, including codon usage. F L9M7 uses long over-represented words (due to the concatenation of the third position of the codon) to make the words found in the compared CDS very specific (because of length) and fitted to the host genome codon usage (considering only third letters of CDS 27-letter words). P L4M2 allows us to characterize CDS by using small and therefore more frequent words distinct from codon usage, in order to obtain a more accurate distribution of words on the CDS.

**Fig. 2:**
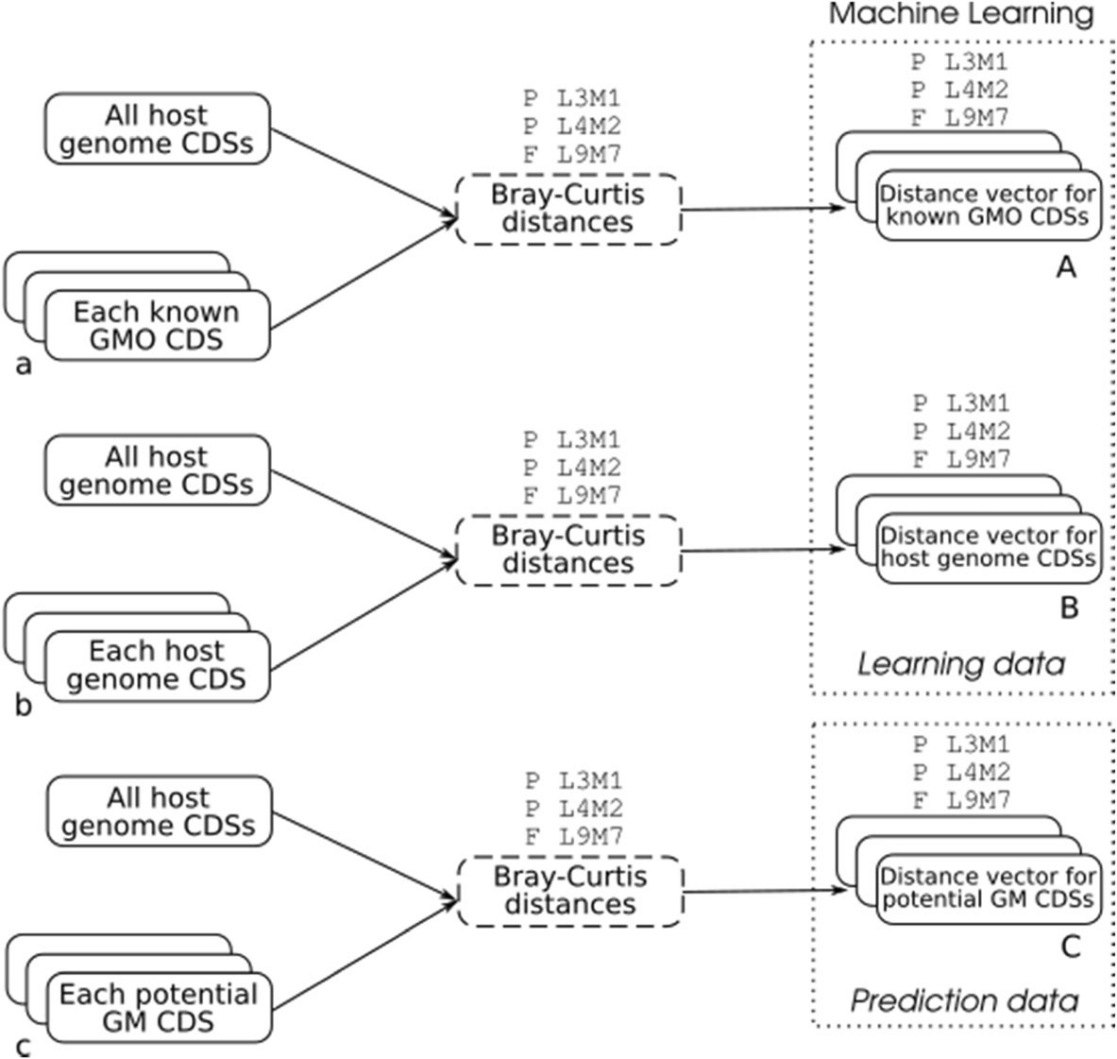
Bray-Curtis distances in proportions (L3M1, L4M2) and frequencies (L9M7) applied to obtain learning and prediction data used in the machine learning step

These Bray-Curtis distance computations (P L3M1, P L4M2 and F L9M7) are performed on each of the three sets of CDSs obtained at the end of the cleaning pipeline (section 2.2), as summarized in Fig. 2, in order to prepare the machine learning step (next paragraph). Note that only CDSs with a length greater than or equal to 27 nucleotides are used, because of the minimum length of the word size considered in the calculation of the Bray-Curtis distance in frequencies.

### Prediction of GM inserts with a machine learning procedure and selection of the method

#### Methods

Our aim is to target true GM insert CDSs. To this end, a large number of classification methods were applied, within a machine learning procedure (caret 6.0_81 package in R 3.5.1 [34]), and the most efficient method in terms of predicting GM CDSs was selected. The 12 classification methods used were: 4 parametric methods: Generalized Linear Model (logit), Stepwise Linear Discriminant Analysis (stepLDA), Stepwise Quadratic Discriminant Analysis (stepQDA) and Partial Least Squares (plsda); 8 non-parametric methods: Neural Networks (nnet), Support Vector Machines with Radial Basis Function Kernel (svmRadial), K-Nearest Neighbours (knn), as well as 5 based on decision trees: Classification Tree (Rpart), Random Forest (RF), Bagging Classification (Treebag), Extreme Gradient Boosting Tree (xgboost), and the supervised classification algorithm C5.0. In this process, a K-fold cross-validation procedure was used. It consists in splitting the whole dataset into K subsets, namely a calibration set (consisting of K-1 subsets) and a validation set (the K remaining subset). In a first step, the calibration set is used to select the parameters of the model and its fitting ability. In a second step, this model is validated on the prediction set to get its prediction ability. This calibration and prediction processes were repeated K times. The parameters of each machine learning method were optimised following a 10-fold cross-validation procedure. The performances of these methods were compared on the basis of a 2-fold cross-validation, in which data were previously stratified and centred. It is worth noting that stratified sampling provides homogeneous distribution of GM CDSs in the sampled data.

#### Criteria to compare methods

*B. subtilis* GM (Data1) was used to select the best predictive method, four performance criteria were proposed: false negative rate, specificity, sensitivity [35], and false positive rate. Among these four criteria, the most crucial one was that the method should not predict a GM insert CDS as a host genome CDS (i.e., no false negatives). The importance of the variables of each method were also calculated. For each method, the ROC curve and the importance of each variable usage among the six available variables were calculated (Additional file 1, section 7).

#### Data

The learning data were the CDSs of the host genome and the databank of filtered known GMO CDSs (Fig. 2a and b). CDSs encoding RNAs were deleted from these learning data. The prediction data were the candidate GM CDSs (Fig. 2c) that did not align with the pangenome (Section 2.2). The associated variables were the Bray-Curtis distances in proportions L4M2, L3M1 and in frequencies L9M7, the length of the CDS, the average *A*_*H*_*(S)* of the exceptionality scores provided by R’MES in the host genome for L4M2 and L9M7 (see Eq. 4), the count density per nucleotide in the CDS for 4 and 9-letter words (sum of the counts *C(m,S)* of all selected words *m*, divided by the length of the CDS *S*) and the CDS GC content. The count density per nucleotide is a way of measuring the proportion of the host exceptional words in the CDS. The average of exceptionality scores is a measure of exceptionality of the words found in the CDS, considering host CDSs composition

4 $$ {A}_H(S)=\frac{\sum_{m\in M}K\left(m,H\right)\times C\left(m,S\right)}{\sum_{m\in M}C\left(m,S\right)} $$

where *K(m,H)* is the R’MES exceptionality score of one word *m* in the host CDSs *H.*

#### Results

**Fig. 3:**
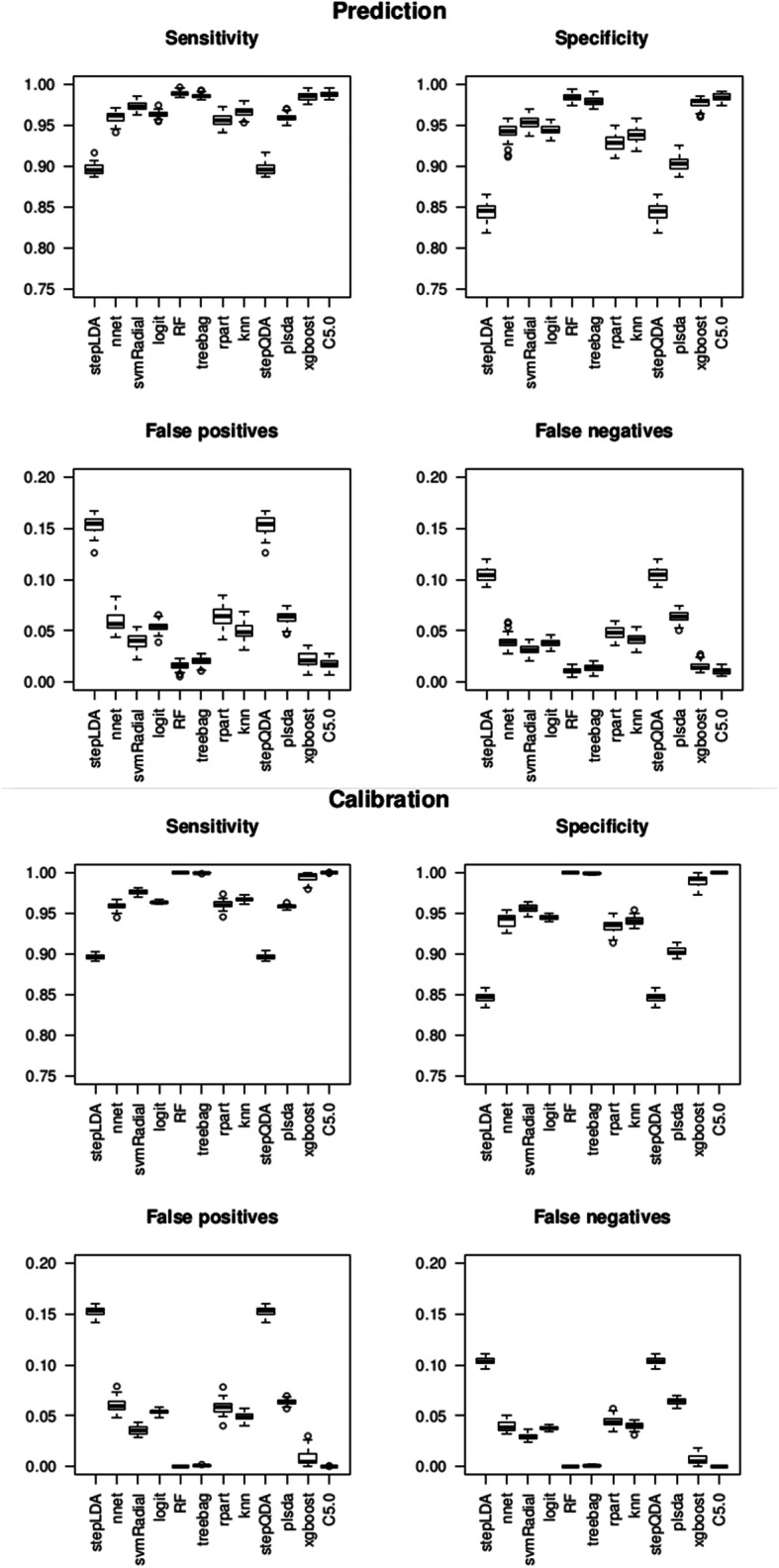
Results of 12 classification methods performed on 50 simulations using the learning data of the *B. subtilis* GM bacteria (Data1). Calibration and prediction results come from the two-fold cross-validation procedures

The results, illustrated in Fig. 3, were obtained from the 12 classification methods run 50 times and applied to the learning data (Fig. 2a and b) of the *B. subtilis* GM bacteria (Data1). These process redundancies ensured robustness of results, some methods being non-deterministic.

The RF, C5.0, Treebag, and xgboost methods gave the best results on calibration and prediction. For calibration purposes, the RF, Treebag and C5.0 methods had a sensitivity and specificity of 1, a false positive and a false negative rate of 0. The xgboost method obtained 0.99 in sensitivity and specificity, and a false positive and a false negative rate of 0.01. For prediction purposes, RF and C5.0 had a sensitivity and a specificity of 0.98, with a positive and a false negative rate of 0.01. The Treebag and xgboost methods had a sensitivity and a specificity of 0.97 with a false positive and a false negative rate of 0.015. For the logit method, the prediction data and the calibration data had the same results. The logit method had a sensitivity of 0.95 and a specificity of 0.94, with a false positive rate of 0.05 and a false negative rate of 0.04.

Following our performance criteria (i.e., false negative rate, specificity, sensitivity and false positive rate), the non-redundant union of positive results of RF and logit methods was retained for the automatic learning step. Then, global sensitivity and specificity of the program were deduced (Additional file 1, section 7.1). The most important variables for predicting CDSs with the Random Forest method were the GC content and the Bray-Curtis distance in proportions L4M2, and with the logit method, Bray-Curtis distances in frequencies L9M7 and proportions L3M1 (Additional file 1, section 7.2). They have complementary selection criteria according to their variable importance, and do not belong to the same method family.

### Application of DUGMO on real data

DUGMO was developed using the data from *B. subtilis* (i.e., Data1, Data2). Validation was conducted on *E. coli* genomes. Table 1 summarizes DUGMO results on the two *B. subtilis* genomes (i.e., Data1, Data2) and on the three *E. coli* genomes. The GM CDSs on GM *B. subtilis* (Data1), correctly predicted by DUGMO, are the main genes located on the plasmids (riboflavin) and the gene conferring resistance to chloramphenicol. Combining the results of the RF and logit methods provides added value for accurately identifying potential GM inserts. At the end of the automatic learning step for the wild type *B. subtilis* (Data2), no CDS was predicted as a GM insert (see line GM *B. subtilis* and Machine learning column in Table 1). With the GM *E. coli* carrying genes of *A. tumefaciens*, *M. tuberculosis* or *S. pyogenes,* the results contained respectively 5, 4 and 5 CDSs predicted as GM inserts. However, one false positive was found in the results of *E. coli* carrying genes of *S. pyogenes*. The CDS found corresponds to an annotated *aslA* gene present in *E. coli* strongly truncated in our strain (984 nt instead of 1650 nt or more for those of the pangenome). In the case of incorporation of a highly truncated protein from a protein family poorly represented in the host genome, this sequence may be considered a GM insert. DUGMO makes it possible to target the insert CDSs if the unknown genome submitted is in fact a GM bacterium.

**Table 1:** DUGMO results for two *B. subtilis* genomes (i.e., Data1, Data2) and three *E. coli* genomes

	Machine Learning						
	Learning data		Prediction data	DUGMO final results			
	Number of host genome CDSs (1)	Number of known GMO CDSs (2)	Number of potential GM CDSs (1)	True positives (3)	False positives (3)	Max false negatives (3) (4)	True negatives (3)
GM *B. subtilis* (Data1)	4102	2714	39	25	0	12	2
Wild type *B. subtilis* (Data2)	3941	2724	4	–	0	–	0
*E. coli* with genes of *A. tumefaciens*	4033	2588	6	5	0	0	1
*E. coli* with genes of *M. tuberculosis*	4018	2589	5	4	0	0	1
*E. coli* with genes of *S. pyogenes*	4015	2587	6	5	1	0	0

(1) After two BLASTN alignments on pangenomes without RNA. (2) After filtering out CDSs of the known GMO databank that are too close to the host species (paragraph 3 of section 2.2). (3) In “potential GM inserts”. The “DUGMO final results” column details the results obtained after combining the results of the RF and logit methods, using the data from the learning data and prediction data columns. (4) Estimation of the maximum number of false negatives: the true number cannot be deduced because of the unknown origins of CDSs, potentially from the Bacillales family. (-) Does not apply

To complete the evaluation of our tool, DUGMO was tested on 6 additional other wild type bacteria: *Campylobacter jejuni*, *Lactococcus lactis*, *Listeria monocytogenes*, *Mycobacterium tuberculosis*, *Salmonella typhimurium* and *Staphylococcus aureus* to check that almost no false positive was present in the results. Then, DUGMO was evaluated on 45 additional tests on synthetic GM samples based on these six bacteria with seven external genes including five genes from these exogenous bacteria, one human gene and one rice gene. Each gene was inserted between two CDS in a reference genome. Then, ART 2.5.8 [36] was used on each modified genome using art_illumina progam with parameters: -ss HS25, −p, −l 150, −f 11, −m 200, −s 10 to create synthetic illumina sequencing data. All the pangenomes used in these tests can be used by users for GM analyses. The pangenomes are provided and the results are detailed in Additional file 1, section 8.

Among the 6 tests on wild type bacteria, we obtained only one false positive, a *M. tuberculosis* gene that we strongly suspect to come from a recent horizontal gene transfer. Indeed, it does not have megablast [37] alignment with a better identity than 94.7% in all genomes of actinomycetes found in NCBI wgs database (01/05/2020). Among the 45 synthetic GM tests, we obtained as few as one false positive, probably due to the open *S. aureus* pangenome and to the environmental and similar genome characteristics of the inserted *C. jejuni* gene (details in Additional file 1 section 8.6).

Then, we assessed the limits of the method with specific synthetic data. We evaluated the robustness of the method to dicodon optimisation. For this purpose, a *B. subtilis* gene encoding a riboflavin synthase, optimised for *E. coli* dicodon usage, was randomly inserted into the reference genome of *E. coli*. Illumina sequencing data were then generated from this genome with ART with the parameters previously described. This procedure was run 10 times. In all cases, DUGMO detected the optimised *B. subtilis* gene as a GM insert, thus proving the method’s insensitivity to dicodon optimisation, therefore codon optimisation.

To evaluate the proportion of substitution needed to make a wild type gene detectable by DUGMO, we artificially introduced substitutions in a wild type gene of *B. subtilis*. Two wild genes were tested independently, a short (417 nucleotide sequence) and a long sequence (1317 nucleotides). The Surfactin long gene and CadI short gene from the wild type *B. subtilis* genome underwent *n* random substitutions, avoiding mutation events that would be too easily detectable by DUGMO such as substitutions in the start codon, the stop codons,and those that introduce an early stop codon in the sequence. Each modified gene was then replaced into the wild type *B. subtilis* assembly. Finally, Illumina sequencing data were generated using ART [36] software as described above. This process was repeated 10 times. The DUGMO results indicate that beyond 9% mutations, a mutated CDS is detected as GM.

## Discussion

We propose a method to detect undescribed GM bacteria. DUGMO is intended to assess the presence of bacterial GM following purification of bacteria, as processed in Paracchini et al. [18]. Once a suspected GM is confirmed by DUGMO, PCR targeting the sequence identified by DUGMO can be employed for routine detection of GM in food or environment samples. DUGMO uses both pangenome and genome species properties to feed machine learning and distinguish GM CDSs from host CDSs. The robustness of the pangenome relies heavily on its completeness achievement, and also on accurate genomes attribution to their respective species when they are submitted to public databanks. Species attribution is most often deduced from 16S RNA leading to frequent errors in attribution and then resulting potentially in mixed species pangenomes [38]. The introduction of synthetic strains in the pangenome may also lead to biased statistical properties, due to inconsistent BLAST matches in the host CDSs sorting steps (section 2.3). This highlights the urgent need for tools dedicated to the assessment of the completeness of a pangenome [39].

Recently, Berbers et al. [40] described a revision of an unauthorized GM *B. subtilis* strain 2014–3557 with one pGMrib plasmid and a 53 kb chromosomal insertion, firstly described (Data1) by Paracchini et al. [18] with four plasmids pGMBsub01–04. For sake of clarity, in following results, we provide within parentheses gene names used in Paracchini et al.. The GM *B. subtilis* contains one incorporated plasmid pGMrib, designed for vitamin B2 production (riboflavin) and several chromosomal modifications. First, the *cat* gene conferring resistance to chloramphenicol is inserted into the chromosomal *recA* gene*.* Second, a 53 kb insertion occurs within the *scpA* gene. Within this integration, several occurrences of the full *ribDEAHT* and partial *ribDEA* operons show high similarity to the *rib* operon of *Bacillus amyloliquefaciens*. In addition, multiple copies of beta-lactamase and kanamycin resistance genes are found in the 53 kb insertion. Lastly, a bleomycin resistance gene is present.

Both the *cat* and *recA* portions of the chromosome are detected as GM CDSs by DUGMO. One *B. subtilis* gene is discarded by the machine learning step, the chromosomal RNA polymerase delta subunit. DUGMO distinguishes the GM *B. amyloliquefaciens scpB* CDS of the chromosomal insert from the wild *B. subtilis scpB* of the pGMrib plasmid. The pGMrib plasmid (plasmid visualization in Additional file 1, section 9) includes two portions. The first one has a pure *B. subtilis ribDEATH* operon that is recognized and discarded by DUGMO and includes *sipS* (*lepB*) and *GAY71_RS22375* (*gerPA*). The second one carries bacilli genes conferring resistance to erythromycin. In this second portion, nine genes are recognized as GM by DUGMO (*GAY71_RS22270* (*repS*), *bin3 / GAY71_RS22275* (*beta*)*, soj* (*delta*)*, zeta, GAY71_RS22265* (*copS*)*, GAY71_RS22365* (*ORF psi*)*, GAY71_RS22350* (*ORF phi*)*, GAY71_RS22355* portion (*ORF chi*)*, and GAY71_RS22370* (*hyprORF-t*)), while the other ten are discarded (*GAY71_RS22250* (*eta*), *GAY71_RS22255* (*theta)*, *GAY71_RS22260* (*iota*), *topB* / *GAY71_RS22280* (*gamma*), *GAY71_RS22290 / GAY71_RS22330 (omega)*, *GAY71_RS22295* / *GAY71_RS22325 (epsilon)*, *erm(B)*, *GAY71_RS22315* with additional portion from nucleotides 14,107 to 14,138 (*ermC*)*, GAY71_RS22340* (*tau-gamma*) and *GAY71_RS22345* (*ypsilon*)). Among them, the *ermC* and *tau-gamma* genes are discarded at the BLASTN steps on the pangenome; interestingly, these genes have a nucleotide sequence very close to the CDSs of *B. subtilis* and are included in host CDSs learning data. Most of the other discarded CDSs match best with enterococci genes, which are gram+ bacilli phylogenetically close to *B. subtilis*. Part of pGMrib (pGMBsub04 of Paracchini et al.) is thought to derive from the patented pMX45 plasmid for which only *B. subtilis* origins and genetic engineering methods are mentioned [18]. However, some plasmids have a very wide host range and asserting their origin is not always possible. In this case, the genetic distance of these ten genes, if they were not of *B. subtilis* origin, does not allow their discrimination. Interestingly, an additional CDS, not described in pGMrib (pGMBsub04 portion) is found twice by DUGMO, once on the plasmid and once on the chromosome. This CDS is present at positions 2652 to 2822 in pGMrib, included in *GAY71_RS22250* (1 to 171 in the pGMBsub04 plasmid), and corresponds to a hypothetical protein of an *Enterococcus faecalis* plasmid (accession number AP018546.1). In addition pGMrib (pGMBsub03 portion) has two additional non *B. subtilis* genes, part of *GAY71_RS22445* (*repB*) and a shortened version of *tet(L)* (*tetR*) found GM in DUGMO results. The 53 kb chromosomal insertion (pGMBsub01 portion) carries genes of different origins (*Bacillus amyloliquefaciens* and *Staphylococcus aureus*). The *S. aureus* genes *GAY71_RS12530* (*bleR*) and *aadD1* (*kanR*) and the *ribD*, *ribA*, *ribT* and *scpB* CDSs of the *B. amyloliquefaciens ribDEATH* operon are correctly detected as GM CDSs by DUGMO. The gene of beta lactamase TEM-116 (a*mpR / ampiR*, six loci in chromosome*,* one in pGMrib), *ribH* and *scpA* CDSs align with the pangenome CDSs in respect to BLASTN parameters, and are included in host CDSs learning data, showing the limits of CDS categorisation for closely related species. This probably also explains the non-detection of *rep* and *ribE*. Prokka annotation provides two additional hypothetical CDSs, matching positions 4759–5103 and 10,112–10,201 of the *B. amyloliquefaciens ribDEATH* operon (pGMBsub01 portion) (loci *GAY71_RS12515*, *GAY71_RS12560*, *GAY71_RS12605*, *GAY71_RS12650*, *GAY71_RS12725*, *GAY71_RS12770* and *GAY71_RS12480*, *GAY71_RS12690* respectively in the chromosome). The second is labelled GM and the first not.

DUGMO, on the *E. coli strain* carrying genes of *S. pyogenes,* found as a false positive CDS that corresponds to a strongly truncated *arlS* CDS (coding for the arylsulfatase protein), whose full-length version is naturally present in the *E. coli* genome. During the analysis, the BLASTN on the pangenome CDSs step verify that the lengths of matching CDSs do not vary by more than 15%. For the other two *E. coli* genomes, carrying the genes of *A. tumefaciens* or *M. tuberculosis*, the *arlS* CDS is full length in the assemblies and is not detected by DUGMO. The truncated *arlS* CDS in *E. coli* carrying genes of *S. pyogenes* is localized at the end of a de novo contig, and the 15% missing part is found in another contig. Therefore, DUGMO is able to detect CDSs that may consist of a long deletion or insertion in a wild CDS. The mean coverage depth of data for this sample is 41, while the coverage depth mean of the other two *E. coli* strains is 63 and 68, respectively. This example indicates that the assembly with an average overlap depth of 41 is partial and leads to a false positive, due to the truncation of a CDS. On the other hand, data assemblies with a coverage depth mean greater than 60 do not have gene truncation problem in our datasets. Most of bacterial assemblers recommend a coverage depth between 80 [41] and 100 (N50 was shown to increase with the coverage depth until a value of 100 for bacterial genomes [42]). A coverage depth of 60 is potentially the acceptable lower limit to obtain a complete assembly without CDS truncation. These observations emphasize the need to obtain a good assembly, and therefore a sufficient coverage depth. In a future version, DUGMO will be able to accept assemblies, including long read assemblies, removing false positive cases due to assembly truncations.

The process of GM bacteria analysis described by Paracchini et al. [18] required more than one month of work (personal communication), while DUGMO needed three hours with 10 threads of a core i7 CPU with 64 GB RAM. The speed of data processing is a major advantage of this tool, which furthermore, does not require prior GMO expertise for end users except to validate the limited set of probable GM genes detected in results. User must have Linux command line skills and have enough biological knowledge to construct a pangenome. In addition, DUGMO has the potential for continuous improvement with the possibility, for the end users, to add new confirmed GMO CDSs to the learning data, and new wild type genomes and CDSs in the pangenome.

## Conclusions

The proposed DUGMO tool combines a high-throughput sequencing data cleaning pipeline with BLASTN alignments on pangenomes, and different Bray-Curtis distance calculations associated with a combination of machine learning methods, the Random Forest and Generalized Linear Model, selected to be the most predictive for GM bacteria data. The tool requires a reliable species-specific pangenome and a reference genome, member of the suspected GM species. Tested on a GM *B. subtilis* and three GM *E. coli*, DUGMO is able to detect exogenous or truncated or fused GM CDS and generates few or no false positives and false negatives. This tool is a proof of concept about the detectability of unknown, single, GM bacteria, based on selected properties of the wild type genome. As DUGMO uses statistical properties related to third positions of CDSs, specific to codon usage, it will not detect GM bacterium with only tRNA or rRNA inserts. Conceptually, DUGMO is made to find CDS that do not use the vocabulary of the host genome. DUGMO is not able to distinguish horizontal transferred gene and GM gene. The usage of DUGMO may be deflected to find in wild type bacteria a gene acquired by recent horizontal gene transfer. This DUGMO usage was not tested. We plan to extend DUGMO to plant and animal GMO detection by adapting the parameters of distance calculations. Finally, for the first time, DUGMO enables fast, systematic detection of unknown GM bacteria in suspicious samples.

## Supplementary information

**Additional file 1.** Supplementary material.

**Additional file 2.** Databank of known GMO CDSs.

**Additional file 3.** List of papers from which known bacterial GM CDSs of the databank were deduced from (format is tabulation separated text file .tsv).

## Data Availability

All data generated or analysed during this study are included in this published article and its supplementary information files.

## Abbreviations

GM: Genetically modified
GMO: Genetically modified organism
CDS: Coding sequence
PCR: Polymerase Chain Reaction
BC: Bray-Curtis
RF: Random Forest
logit: Generalized Linear Model
