# Supplementary material for: DUGMO: tool for the detection of unknown genetically modified organisms with high-throughput sequencing data for pure bacterial samples
Source: BMC Bioinformatics. 2020 Jul 6;21:284. doi: 10.1186/s12859-020-03611-5 (PMC7336441; doi:10.1186/s12859-020-03611-5)
Supplement: Supplementary file 1 — Additional file 1. Supplementary material. [file 12859_2020_3611_MOESM1_ESM.docx]

**Supplementary Material**

GMO_inserts.fa: List of CDS inserts of bacterial GMOs from the literature

**1 Diagram illustrating the two BLASTN steps of potential GMO insert sequences against pangenome**

**
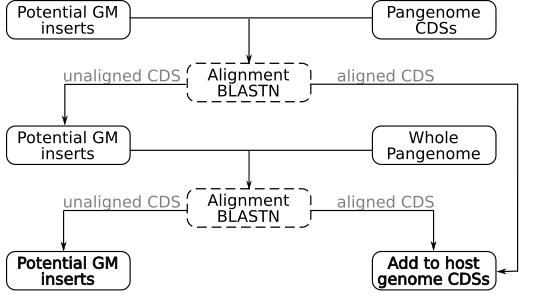
**

Figure S1: Diagram of two BLASTN with pangenome CDSs and whole pangenome

**2 List of the 37 genomes used to make the *Bacillus Subtilis* pangenome (partially adapted from** **(1)****)**

| **Organism/name** | **NCBI Accession number** | **Nb of genes** | **Size (Mb)** | **Level** |
| --- | --- | --- | --- | --- |
| *Bacillus* *subtilis* strain BJ3-2 | CP025941.1 | 4443 | 4.2 | complete |
| *Bacillus* *subtilis* subsp. *subtilis* strain BS155 | CP029052.1 | 4576 | 4.3 | complete |
| *Bacillus* *subtilis* subsp. *subtilis* str. OH 131.1 | CP007409.1 | 4062 | 4.0 | complete |
| *Bacillus* *subtilis* subsp. *subtilis* str. JH642 substr. AG174 | CP007800.1 | 4361 | 4.2 | complete |
| *Bacillus* *subtilis* subsp. *subtilis* str. AG1839 | CP008698.1 | 4361 | 4.2 | complete |
| *Bacillus* *subtilis* strain B-1 | CP009684.1 | 3868 | 3.9 | genome |
| *Bacillus* *subtilis* subsp. *subtilis* strain IIG-Bs27-47-24 | CP016787.1 | 3035 | 2.9 | genome |
| *Bacillus* *subtilis* subsp. *subtilis* strain PG10 | CP016788.1 | 2890 | 2.8 | genome |
| *Bacillus* *subtilis* subsp. *subtilis* strain PS38 | CP016789.1 | 2824 | 2.7 | genome |
| *Bacillus* *subtilis* subsp. *subtilis* strain QB5412 | CP017312.1 | 4436 | 4.2 | genome |
| *Bacillus* *subtilis* subsp. *subtilis* strain QB5413 | CP017313.1 | 5248 | 4.2 | genome |
| *Bacillus* *subtilis* strain SR1 | CP021985.1 | 4155 | 4.1 | genome |
| *Bacillus* *subtilis* subsp. spizizenii strain SW83 | CP030925.1 | 4126 | 4.0 | chromosome |
| *Bacillus* *subtilis* subsp. *subtilis* strain IITK SM1 | CP031675.1 | 4238 | 4.0 | chromosome |
| *Bacillus* *subtilis* strain FB6-3 | CP032089.1 | 4105 | 4.2 | chromosome |
| *Bacillus* *subtilis* strain MZK05 | CP032315.1 | 4352 | 4.1 | complete |
| *Bacillus* *subtilis* strain SRCM104005 | CP035164.1 | 4325 | 4.1 | complete |
| *Bacillus* *subtilis* strain SRCM103571 | CP035231.1 | 4329 | 4.1 | complete |
| *Bacillus* *subtilis* strain SRCM103835 | CP035400.1 | 4325 | 4.1 | complete |
| *Bacillus* *subtilis* strain SRCM103622 | CP035411.1 | 4385 | 4.1 | complete |
| *Bacillus* *subtilis* strain SRCM103629 | CP035413.1 | 4306 | 4.1 | complete |
| *Bacillus* *subtilis* subsp. *subtilis* str. 168 | NC_000964.3 | 4106 | 4.2 | complete |
| *Bacillus* *subtilis* subsp. spizizenii str. W23 | NC_014479.1 | 4116 | 4.0 | complete |
| *Bacillus* *subtilis* BSn5 | NC_014976.1 | 4237 | 4.1 | complete |
| *Bacillus* *subtilis* subsp. spizizenii TU-B-10 | NC_016047.1 | 4315 | 4.2 | complete |
| *Bacillus* *subtilis* subsp. *subtilis* RO-NN-1 | NC_017195.1 | 4115 | 4.0 | complete |
| *Bacillus* *subtilis* subsp. natto BEST195 DNA | NC_017196.2 | 4366 | 4.1 | complete |
| *Bacillus* sp. JS | NC_017743.1 | 4185 | 4.1 | complete |
| *Bacillus* *subtilis* QB928 | NC_018520.1 | 4332 | 4.1 | complete |
| *Bacillus* *subtilis* subsp. *subtilis* str. BSP1 | NC_019896.1 | 4155 | 4.0 | complete |
| *Bacillus* *subtilis* XF-1 | NC_020244.1 | 4175 | 4.0 | complete |
| *Bacillus* *subtilis* subsp. *subtilis* 6051-HGW | NC_020507.1 | 4422 | 4.2 | complete |
| *Bacillus* *subtilis* subsp. *subtilis* str. BAB-1 | NC_020832.1 | 4124 | 4.0 | complete |
| *Bacillus* *subtilis* PY79 | NC_022898.1 | 4187 | 4.0 | complete |
| *Bacillus* *subtilis* strain SG6 | NZ_CP009796.1 | 4223 | 4.0 | complete |
| *Bacillus* *subtilis* strain B4146 | NZ_JXHR01000001.1 | 4464 | 4.0 | assembly |
| *Bacillus* *subtilis* strain HDZK-BYSB7 | CP026608.1 | 5695 | 5.3 | complete |

**3 List of the 45 genomes used to make the *Echerichia coli* pangenome (partially adapted from** **(2))**

| Organism/name | NCBI Accession number | Nb of genes | Size (Mb) | Level |
| --- | --- | --- | --- | --- |
| *Escherichia coli* B strain C2566 | CP014268.2 | 4428 | 4.4 | complete |
| *Escherichia coli* 0127:H6 strain E2348/69 | NC_011601.1 | 5388 | 4.9 | complete |
| *Escherichia coli* ST131 strain EC958 | NZ_HG941718.1 | 5472 | 5.1 | complete |
| *Escherichia coli* BL21(DE3) | NC_012892.2 | 4700 | 4.5 | complete |
| *Escherichia coli* strain BA22372 | NZ_CP040397.1 | 4833 | 4.7 | complete |
| *Escherichia coli* strain RM14715 | NZ_CP027104.1 | 4852 | 4.8 | complete |
| *Escherichia coli* strain FORC_081 | NZ_CP029057.1 | 5138 | 4.7 | complete |
| *Escherichia coli* strain WCHEC025970 | NZ_CP036177.1 | 5284 | 4.7 | complete |
| *Escherichia coli* strain 2012C-4227 | NZ_CP013029.1 | 5534 | 5.2 | complete |
| *Escherichia coli* Nissle 1917 | NZ_CP007799.1 | 5495 | 5.4 | complete |
| *Escherichia coli* UM146 | NC_017632.1 | 5049 | 4.9 | complete |
| *Escherichia coli* CFT073 | NC_004431.1 | 5169 | 5.2 | complete |
| *Escherichia coli* strain ECZP248 | NZ_CP034784.1 | 4812 | 4.4 | complete |
| *Escherichia coli* strain BH100 substr. MG2014 | NZ_CP024650.2 | 5102 | 5.0 | complete |
| *Escherichia coli* strain FAM21845 | NZ_CP017220.1 | 5139 | 4.9 | complete |
| *Escherichia coli* strain SCEC020022 | NZ_CP032892.1 | 5417 | 4.9 | complete |
| *Escherichia coli* str. K-12 substr. MG1655 | NC_000913.3 | 4395 | 4.6 | complete |
| *Escherichia coli* strain WCHEC025943 | NZ_CP027205.2 | 6343 | 4.8 | complete |
| *Escherichia coli* strain FORC_069 | NZ_CP023061.1 | 6478 | 5.1 | complete |
| *Escherichia coli* strain cq9 | NZ_CP031546.1 | 7284 | 5.2 | complete |
| *Escherichia coli* O157:H7 str. Sakai DNA | NC_002695.2 | 5361 | 5.5 | complete |
| *Escherichia coli* strain FORC 064 | NZ_CP022664.1 | 5405 | 4.8 | complete |
| *Escherichia coli* APEC O2-211 | NZ_CP006834.2 | 5565 | 5.1 | complete |
| *Escherichia coli* strain EC590 | NZ_CP016182.2 | 4788 | 4.6 | complete |
| *Escherichia coli* str. Sanji | NZ_CP011061.1 | 5702 | 4.9 | complete |
| *Escherichia coli* strain SF-088 | NZ_CP012635.1 | 5474 | 5.0 | complete |
| *Escherichia coli* strain 2009C-3133 | NZ_CP013025.1 | 5860 | 5.1 | complete |
| *Escherichia coli* strain CFSAN029787 | NZ_CP011416.1 | 5633 | 4.9 | complete |
| *Escherichia coli* O145:H28 str. RM12581 | NZ_CP007136.1 | 6116 | 5.5 | complete |
| *Escherichia coli* strain ST2747 | NZ_CP007393.1 | 5039 | 5.0 | complete |
| *Escherichia coli* S88 | NC_011742.1 | 5427 | 5.0 | complete |
| *Escherichia coli* UMN026 | NC_011751.1 | 5033 | 5.2 | complete |
| *Escherichia coli* APEC O18 | NZ_CP006830.1 | 5149 | 5.0 | complete |
| *Escherichia coli* strain D9 | NZ_CP010152.1 | 5006 | 4.6 | complete |
| *Escherichia coli* strain D9 plasmid A | NZ_CP010153.1 | 130 | 0.1 | plasmid |
| *Escherichia coli* strain D9 plasmid B | NZ_CP010154.1 | 87 | 0.08 | plasmid |
| *Escherichia coli* strain D9 plasmid C | NZ_CP010155.1 | 52 | 0.04 | plasmid |
| *Escherichia coli* strain D9 plasmid D | NZ_CP010156.1 | 7 | 0.005 | plasmid |
| *Escherichia coli* strain S50 | NZ_CP010238.1 | 5239 | 4.8 | complete |
| *Escherichia coli* strain M8 | NZ_CP010191.1 | 5232 | 4.8 | complete |
| *Escherichia coli* strain M8 plasmid A | NZ_CP010192.1 | 185 | 0.2 | plasmid |
| *Escherichia coli* strain M8 plasmid B | NZ_CP010193.1 | 57 | 0.04 | plasmid |
| *Escherichia coli* strain M8 plasmid C | NZ_CP010194.1 | 46 | 0.03 | plasmid |
| *Escherichia coli* strain M8 plasmid D | NZ_CP010195.1 | 11 | 0.005 | plasmid |
| *Escherichia coli* strain C8 | NZ_CP010125.1 | 5233 | 4.9 | complete |
| *Escherichia coli* strain C8 plasmid A | NZ_CP010126.1 | 181 | 0.1 | plasmid |
| *Escherichia coli* strain C8 plasmid B | NZ_CP010127.1 | 52 | 0.04 | plasmid |
| *Escherichia coli* strain C8 plasmid C | NZ_CP010128.1 | 14 | 0.008 | plasmid |
| *Escherichia coli* strain H3 | NZ_CP010167.1 | 4797 | 4.6 | complete |
| *Escherichia coli* strain H3 plasmid A | NZ_CP010168.1 | 59 | 0.05 | plasmid |
| *Escherichia coli* strain H1 | NZ_CP010160.1 | 4994 | 4.8 | complete |
| *Escherichia coli* strain H1 plasmid A | NZ_CP010161.1 | 7 | 0.004 | plasmid |
| *Escherichia coli* strain H1 plasmid B | NZ_CP010162.1 | 4 | 0.004 | plasmid |
| *Escherichia coli* strain S3 | NZ_CP010228.1 | 4773 | 4.6 | complete |
| *Escherichia coli* O145:H28 str. RM13516 | NZ_CP006262.1 | 5899 | 5.4 | complete |
| *Escherichia coli* KO11 | NC_016902.1 | 5217 | 4.9 | complete |
| *Escherichia coli* KO11 plasmid pEKO1102 | NC_016903.1 | 9 | 0.005 | plasmid |
| *Escherichia coli* KO11 plasmid pEKO1101 | NC_016904.1 | 136 | 0.1 | plasmid |
| *Escherichia coli* IAI1 | NC_011741.1 | 4786 | 4.7 | complete |
| *Escherichia coli* APEC O1 | NC_008563.1 | 5543 | 5.0 | complete |
| *Escherichia coli* IAI39 | NC_011750.1 | 4936 | 5.1 | complete |
| *Escherichia coli* ST131 strain EC958 plasmid pEC958 | NZ_HG941719.1 | 173 | 0.1 | plasmid |
| *Escherichia coli* ST131 strain EC958 plasmid pEC958B | NZ_HG941720.1 | 4 | 0.004 | plasmid |
| *Escherichia coli* UM146 plasmid pUM146 | NC_017630.1 | 157 | 0.1 | plasmid |
| *Escherichia coli* strain SF-088 plasmid pSF-088-1 | NZ_CP012636.1 | 192 | 0.1 | plasmid |
| *Escherichia coli* strain SF-088 plasmid pSF-088-2 | NZ_CP012637.1 | 5 | 0.005 | plasmid |
| *Escherichia coli* strain SF-088 plasmid pSF-088-3 | NZ_CP012638.1 | 3 | 0.004 | plasmid |
| *Escherichia coli* O145:H28 str. RM12581 plasmid pRM12581 | NZ_CP007137.1 | 97 | 0.06 | plasmid |
| *Escherichia coli* O145:H28 str. RM12581 plasmid pO145-12581 | NZ_CP007138.1 | 102 | 0.09 | plasmid |

**4 BLASTN on pangenome parameters**

**4.1 First BLASTN parameters**

For the BLASTN on pangenome CDSs, the options gapopen and gapextend are set respectively to 3 and 1 to avoid cutting proteins with an internal gap. The retained CDSs have an identity percentage greater than 98%, or a percent query coverage per HSP (High-scoring Segment Pair) greater than 98% and an identity percentage greater than 95%. Then, only alignments whose length is included in an interval of plus or minus 15% of the length of the potential GM CDS (query length) and of the length of the CDS of the pangenome (subject length) are retained to ensure a high percentage of alignment coverage on CDS with a long gap on BLASTN of pangenome CDSs.

**4.2 Second BLASTN parameters**

For the BLASTN on the pangenome, the options gapopen and gapextend are set respectively to 3 and 1 to avoid cutting proteins with an internal gap. The retained CDSs have an an identity percentage greater than 95% and a percent query coverage per HSP greater than 98%. Only alignments whose length is included in an interval of plus or minus 15% of the length of the potential GM CDS (query length) are retained.

**4.3 Third BLASTN parameters**

For the BLASTN on the GMO databank, the options gapopen and gapextend are set respectively to 3 and 1 to avoid cutting proteins with an internal gap. The retained CDSs have an identity percentage greater than 98%, or a percent query coverage per HSP (High-scoring Segment Pair) greater than 98% and an identity percentage greater than 95%.

**5 Different distances used: Euclidean, Kullback-Leibler and Bray-Curtis**

Three distances were tested, the Euclidean distance, the Bray-Curtis distance and the Kullback-Leibler distance. Bray-Curtis is directly applicable on frequencies, Kullback-Leibler is used in the detection of viral sequences close to a known but divergent virus (3) and Euclidean is a simple, classical and known distance. The first tests were carried out on a GMO maize genome, based on these results on different R'MES analyses, the Euclidean distance has been removed. It did not discriminate GMO CDSs and the CDS of the reference maize and obtained less results compared to the other two distances. We also tested the bacterium *Streptomyces avermitilis* where analyses showed that the exceptionality scores calculated by R'MES with Euclidean distance decreased with the size of words. Contrary to other distances, with Euclidian distance no clear separation of GM CDS was observed although different R'MES parameters were used (word size, phase 3, Markov order). The distances of Bray-Curtis and Kullback-Leibler often present similar results although their properties are totally different. The final choice was the Bray-Curtis distance because a higher number of GM inserts were found with the Bray-Curtis distance.

**6 Justification of the concatenation of third positions of codons**

The third positions are the most representative of the use of codons by the bacteria or the organisms (table 2 (4)). We wanted to make R’MES analyzing words of 27 nucleotides in phase 3. The phase parameter is used to take into account the periodicity of the sequence, in our case every three nucleotides, the third position of the codon being the most specific. Calculations on words of 27 nucleotides with R'MES are impossible (the maximum word size is 14) but statistical results are based only on the third codon positions. The same results can be obtained by analyzing 9 letters words of sequences where only third codon positions were retained. The use of R'MES is therefore possible to obtain results equivalent to those of 27-letters word analysis.

**7 Evaluation of machine learning methods on the GM *B. subtilis* genome**

**7.1 Calibration and prediction results from the non-redundant union of RF and logit methods performed on 50 simulations using the learning data of the *B. subtilis* GM bacteria**

**
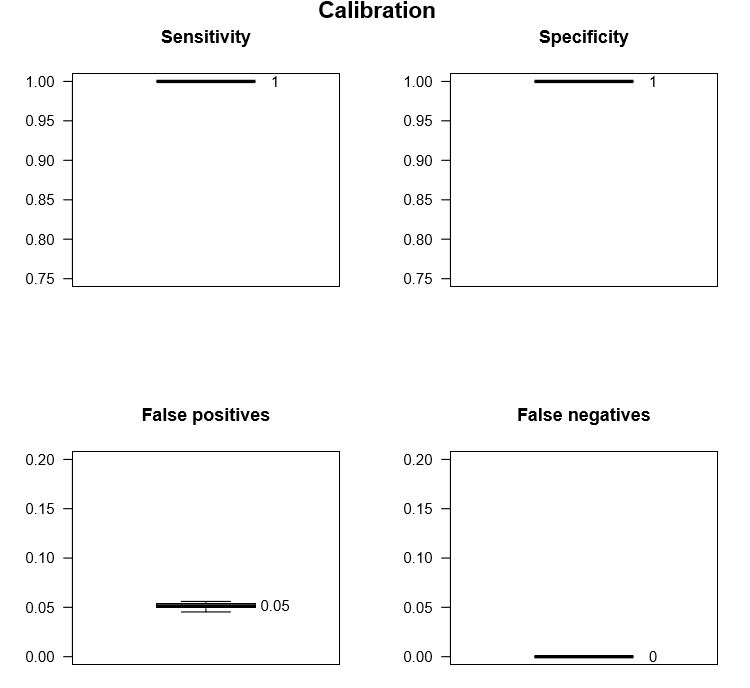
**

**
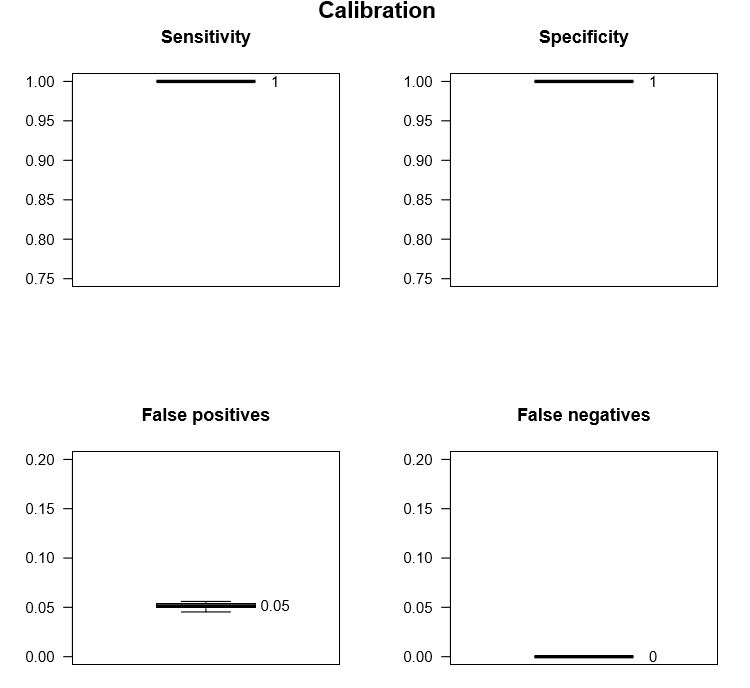
**

**
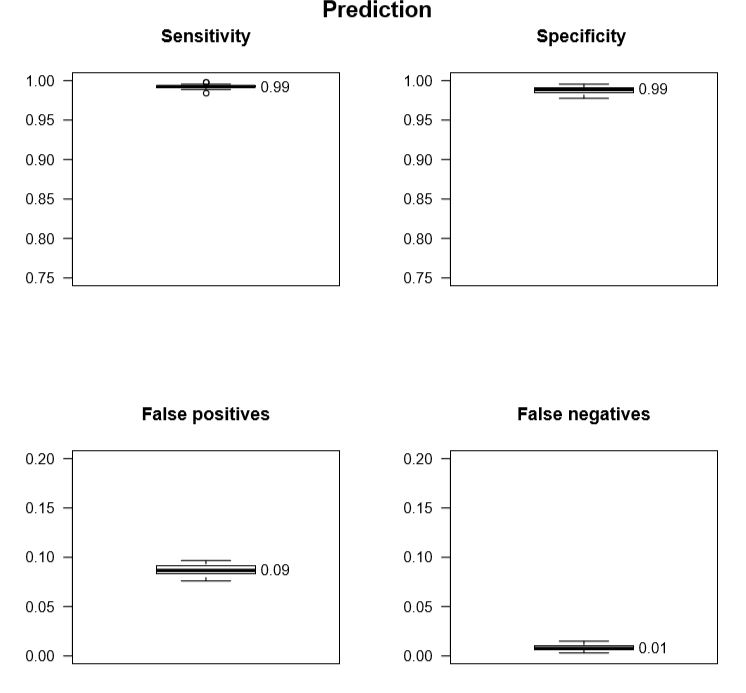
**

**
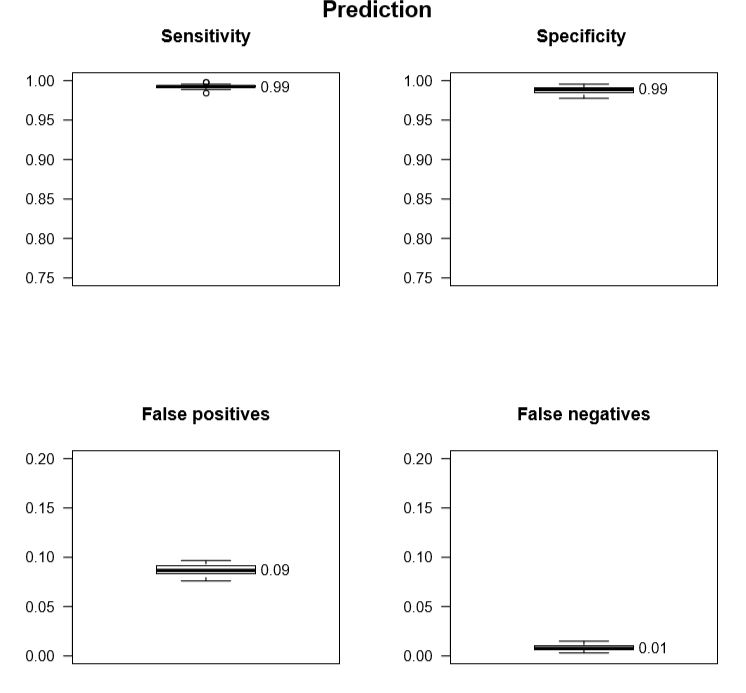
**


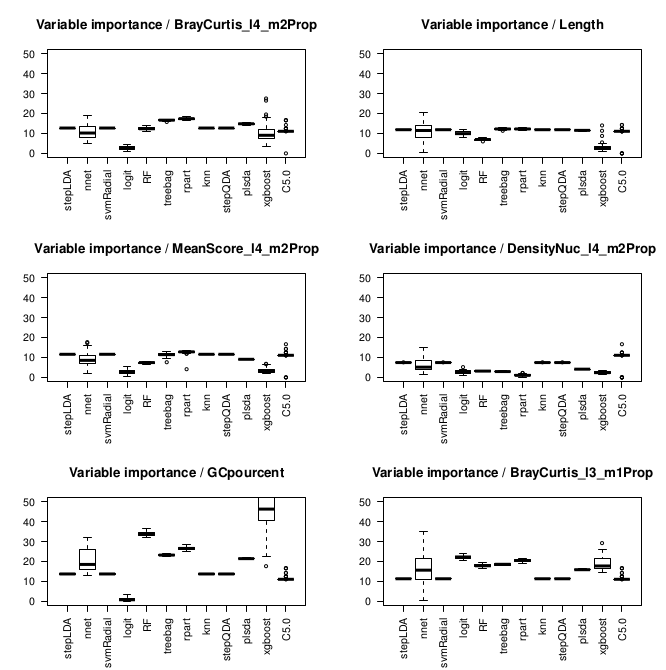
**7.2 Graphics for evaluation of variable importance on the GM *B. subtilis* genome**

**
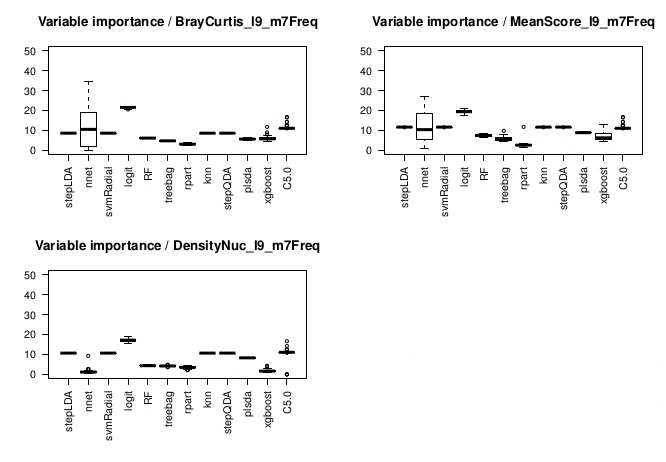
**


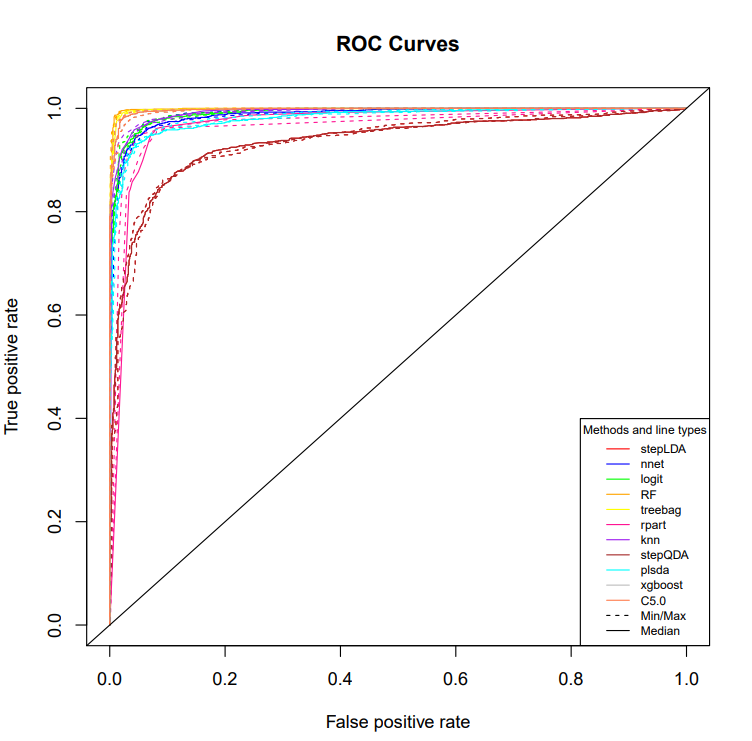
**7.3 ROC Curves on the GM *B. subtilis* genome**

**8 DUGMO results and associated pangenomes on 6 bacteria: *Campylobacter jejuni*, *Lactococcus lactis*, *Listeria monocytogenes*, *Mycobacterium tuberculosis*, *Salmonella typhimurium* and *Staphylococcus aureu*s**

Analyses were run on the following wild bactera: *Campylobacter jejuni* subsp. jejuni NCTC 11168

(accession: AL111168.1, SRX6930849), *Lactococcus lactis* subsp. Lactis Il1403 (accession: AE005176.1, SRX4873416), *Listeria monocytogenes* EGD-e (accession: NC_003210.1, SRX7828066), *Mycobacterium tuberculosis* H37Rv (accession: NC_000962.3, ERX3965724), *Salmonella enterica* subsp. enterica serovar Typhimurium str. LT2 (accession: AE006468.2, SRX7828483), *Staphylococcus aureus* subsp. aureus NCTC 8325 (accession: NC_007795.1, SRX7817854).

Genes used to simulate GM bacteria were: *Campylobacter jejuni* *ansA* gene (CAL34210.1_29, Cj0029 locus in AL111168.1), *Lactococcus lactis* *ydgG* gene (AAK04455.1_357, L162952 locus in AE005176.1), *Listeria monocytogenes lmo0075* gene (NP_463608.1_75, lmo0075 locus in NC_003210.1), *Mycobacterium tuberculosis* Rv1478 gene (NP_215994.1_1477, Rv1478 locus in NC_000962.3), *Salmonella typhimurium* Gifsy-1 prophage gene (AAL21510.1_2546, STM2616 locus in AE006468.2), *Staphylococcus aureus* YP_501325.1_2598 CDS (SAOUHSC_02869 locus in NC_007795.1), *Homo sapiens RHEX* gene (NP_001356419.1_9568 from NC_000001.11), and *Oryza sativa* Japonica Group putative *eIF4E* gene cultivar Azucena (in AM411441.1).

Genome accession numbers of pangenomes are provided in section 8.7 (*Campylobacter jejuni*), 8.8 (*Lactococcus lactis*), 8.9 (*Listeria monocytogenes*), 8.10 (*Staphylococcus aureus*), 8.11 (*Mycobacterium tuberculosis*), 8.12 (*Salmonella typhymrium*).

For next tables, (1) After two BLASTN alignments on pangenomes without RNA. (2) After filtering out CDSs of the known GMO databank that are too close to the host species (paragraph 3 of section 2.2). (3) In “potential GM inserts”. The “DUGMO final results” column details the results obtained after combining the results of the RF and logit methods, using the data from the learning data and prediction data columns.

**8.1 DUGMO results on *Campylobacter jejuni***

|  | **Machine Learning** | | |  | | | |
| --- | --- | --- | --- | --- | --- | --- | --- |
|  | **Learning data** | | **Prediction data** | **DUGMO final results** | | | |
|  | Number of host genome CDSs (1) | Number of known GMO CDSs (2) | Number of potential GM CDSs (1) | True positives (3) | False positives (3) | False negatives (3) | True negatives (3) |
| Wild type *C. jejuni* | 1561 | 2794 | 26 | - | 0 | - | 26 |
| *C. jejuni* with gene of *L. lactis* | 1441 | 2794 | 1 | 1 | 0 | 0 | 0 |
| *C. jejuni* with gene of *L. monocytogenes* | 1344 | 2794 | 1 | 1 | 0 | 0 | 0 |
| *C. jejuni* with gene of *S. aureus* | 1391 | 2794 | 1 | 1 | 0 | 0 | 0 |
| *C. jejuni* with gene of *M. tuberculosis* | 1407 | 2794 | 1 | 1 | 0 | 0 | 0 |
| *C. jejuni* with gene of *S. typhimurium* | 1434 | 2794 | 1 | 1 | 0 | 0 | 0 |
| *C. jejuni* with gene of *O. sativa* | 1383 | 2794 | 1 | 1 | 0 | 0 | 0 |
| *C. jejuni* with gene of *H. sapiens* | 1385 | 2794 | 1 | 1 | 0 | 0 | 0 |

No CDS were detected as an insert in DUGMO results on the wild type *C. jejuni* genome and on synthetics genomes.

**8.2 DUGMO results on *Lactococcus lactis***

|  | **Machine Learning** | | |  | | | |
| --- | --- | --- | --- | --- | --- | --- | --- |
|  | **Learning data** | | **Prediction data** | **DUGMO final results** | | | |
|  | Number of host genome CDSs (1) | Number of known GMO CDSs (2) | Number of potential GM CDSs (1) | True positives (3) | False positives (3) | False negatives (3) | True negatives (3) |
| Wild type *L. lactis* | 1358 | 2786 | 0 | - | 0 | - | 0 |
| *L. lactis* with gene of *C. jejuni* | 1928 | 2784 | 1 | 1 | 0 | 0 | 0 |
| *L. lactis* with gene of *L. monocytogenes* | 1924 | 2779 | 1 | 1 | 0 | 0 | 0 |
| *L. lactis* with gene of *S. aureus* | 1904 | 2780 | 1 | 1 | 0 | 0 | 0 |
| *L. lactis* with gene of *M. tuberculosis* | 1916 | 2783 | 1 | 1 | 0 | 0 | 0 |
| *L. lactis* with gene of *S. typhimurium* | 1951 | 2778 | 1 | 1 | 0 | 0 | 0 |
| *L. lactis* with gene of *O. sativa* | 1933 | 2779 | 1 | 1 | 0 | 0 | 0 |
| *L. lactis* with gene of *H. sapiens* | 1940 | 2779 | 1 | 1 | 0 | 0 | 0 |

No CDS were detected as an insert in DUGMO results on the wild type *L. lactis* genome and on synthetics genomes. However, for the analysis of the wild type *L. lactis* genome, the minimum of coverage depth after assembly has been modified to 50 (mincov option in Shovill). Indeed, the presence of fish contamination was detected in these sequencing data.

**8.3 DUGMO results on *Listeria monocytogenes***

|  | **Machine Learning** | | |  | | | |
| --- | --- | --- | --- | --- | --- | --- | --- |
|  | **Learning data** | | **Prediction data** | **DUGMO final results** | | | |
|  | Number of host genome CDSs (1) | Number of known GMO CDSs (2) | Number of potential GM CDSs (1) | True positives (3) | False positives (3) | False negatives (3) | True negatives (3) |
| Wild type *L. monocytogenes* | 2753 | 2778 | 0 | - | 0 | - | 0 |
| *L. monocytogenes* with gene of *L. lactis* | 2495 | 2782 | 1 | 1 | 0 | 0 | 0 |
| *L. monocytogenes* with gene of *C. jejuni* | 2525 | 2778 | 1 | 1 | 0 | 0 | 0 |
| *L. monocytogenes* with gene of *S. aureus* | 2491 | 2780 | 1 | 1 | 0 | 0 | 0 |
| *L. monocytogenes* with gene of *M. tuberculosis* | 2547 | 2780 | 1 | 1 | 0 | 0 | 0 |
| *L. monocytogenes* with gene of *S. typhimurium* | 2397 | 2782 | 1 | 1 | 0 | 0 | 0 |
| *L. monocytogenes* with gene of *O. sativa* | 2463 | 2779 | 1 | 1 | 0 | 0 | 0 |
| *L. monocytogenes* with gene of *H. sapiens* | 2788 | 2782 | 1 | 1 | 0 | 0 | 0 |

No CDS were detected as an insert in DUGMO results on the wild type *L. monocytogenes* genome and on synthetics genomes. The coverage depth option of ART has been changed to -f18 for *L. monocytogenes* with gene of *H. sapiens.* The coverage depth (option -f11 in ART) was not sufficient to complete the alignment BWA step during the cleaning pipeline.

**8.4 DUGMO results on *Mycobacterium tuberculosis***

|  | **Machine Learning** | | |  | | | |
| --- | --- | --- | --- | --- | --- | --- | --- |
|  | **Learning data** | | **Prediction data** | **DUGMO final results** | | | |
|  | Number of host genome CDSs (1) | Number of known GMO CDSs (2) | Number of potential GM CDSs (1) | True positives (3) | False positives (3) | False negatives (3) (4) | True negatives (3) |
| Wild type *M. tuberculosis* | 4001 | 2809 | 14 | - | 1 | - | 13 |
| *M. tuberculosis* with gene of *L. lactis* | 3695 | 2808 | 1 | 1 | 0 | 0 | 0 |
| *M. tuberculosis* with gene of *L. monocytogenes* | 3646 | 2807 | 1 | 1 | 0 | 0 | 0 |
| *M. tuberculosis* with gene of *S. aureus* | 3593 | 2808 | 2 | 2 | 0 | 0 | 0 |
| *M. tuberculosis* with gene of *C. jejuni* | 3657 | 2808 | 1 | 1 | 0 | 0 | 0 |
| *M. tuberculosis* with gene of *S. typhimurium* | 3699 | 2806 | 1 | 1 | 0 | 0 | 0 |
| *M. tuberculosis* with gene of *O. sativa* | 3676 | 2808 | 1 | 1 | 0 | 0 | 0 |
| *M. tuberculosis* with gene of *H. sapiens* | 3685 | 2807 | 1 | 1 | 0 | 0 | 0 |

In the DUGMO results on the wild type *M. tuberculosis* genome, one CDS was found as an insert. This CDS has less than 95% percentage identity when BLASTN alignment was performed on whole pangenome of *M. tuberculosis*. This CDS may come from a horizontal gene transfer from an unidentified bacterium. A MEGABLAST alignment of this CDS was run on WGS database (exhaustive environmental samples of NCBI databank) on all actinomycetes but no better results were provided. Therefore, the origin of this CDS could not be determined. The coverage depth option of ART has been changed to -f18 for *M. tuberculosis* with gene of *L. lactis.* The coverage depth (option -f11 in ART) was not sufficient to complete the alignment BWA step during the cleaning pipeline.

**8.5 DUGMO results on *Salmonella typhimurium***

|  | **Machine Learning** | | |  | | | |
| --- | --- | --- | --- | --- | --- | --- | --- |
|  | **Learning data** | | **Prediction data** | **DUGMO final results** | | | |
|  | Number of host genome CDSs (1) | Number of known GMO CDSs (2) | Number of potential GM CDSs (1) | True positives (3) | False positives (3) | False negatives (3) | True negatives (3) |
| Wild type *S. typhimurium* | 4629 | 2574 | 0 | - | 0 | - | 0 |
| *S. typhimurium* with gene of *L. lactis* | 4080 | 2573 | 2 | 1 | 0 | 0 | 1 |
| *S. typhimurium* with gene of *L. monocytogenes* | 4065 | 2571 | 0 | 1 | 0 | 0 | 0 |
| *S. typhimurium* with gene of *S. aureus* | 4141 | 2572 | 1 | 1 | 0 | 0 | 0 |
| *S. typhimurium* with gene of *M. tuberculosis* | 4106 | 2573 | 1 | 1 | 0 | 0 | 0 |
| *S. typhimurium* with gene of *C. jejuni* | 4145 | 2570 | 1 | 1 | 0 | 0 | 0 |
| *S. typhimurium* with gene of *O. sativa* | 4089 | 2573 | 1 | 1 | 0 | 0 | 0 |
| *S. typhimurium* with gene of *H. sapiens* | 4501 | 2574 | 1 | 1 | 0 | 0 | 0 |

No CDS were detected as an insert in DUGMO results on the wild type *S. typhimurium* genome and on synthetics genomes. The coverage depth option of ART has been changed to -f18 for *S. typhimurium* with gene of *H. sapiens*. The coverage depth (option -f11 in ART) was not sufficient to complete the alignment BWA step during the cleaning pipeline.

**8.6 DUGMO results on *Staphylococcus aureus***

|  | **Machine Learning** | | |  | | | |
| --- | --- | --- | --- | --- | --- | --- | --- |
|  | **Learning data** | | **Prediction data** | **DUGMO final results** | | | |
|  | Number of host genome CDSs (1) | Number of known GMO CDSs (2) | Number of potential GM CDSs (1) | True positives (3) | False positives (3) | False negatives (3) | True negatives (3) |
| Wild type *S. aureus* | 2485 | 2794 | 2 | - | 0 | - | 2 |
| *S. aureus* with gene of *L. lactis* | 2267 | 2794 | 1 | 0 | 0 | 0 | 0 |
| *S. aureus* with gene of *L. monocytogenes* | 2204 | 2792 | 1 | 1 | 0 | 0 | 0 |
| *S. aureus* with gene of *C. jejuni* | 2216 | 2793 | 1 | 0 | 0 | 1 | 0 |
| *S. aureus* with gene of *M. tuberculosis* | 2251 | 2791 | 1 | 1 | 0 | 0 | 0 |
| *S. aureus* with gene of *S. typhimurium* | 2190 | 2783 | 1 | 1 | 0 | 0 | 0 |
| *S. aureus* with gene of *O. sativa* | 2544 | 2793 | 1 | 1 | 0 | 0 | 0 |
| *S. aureus* with gene of *H. sapiens* | 2156 | 2792 | 1 | 1 | 0 | 0 | 0 |

For the analysis of the wild type *S.aureus* genome, we add the option -dust no in the BLASTN alignment on whole pangenome because this option mask regions of low complexity by default. One CDS was found as a false negative when this option is set by default. The false negative present in the analysis of *S. aureus* with gene of *C. jejuni* is the result of the RF and Logit methods that predict this GM CDS insert as a wild type CDS. The coverage depth option of ART has been changed to -f18 for *S. aureus* with gene of *O. sativa*. The coverage depth (option -f11 in ART) was not sufficient to complete the alignment BWA step during the cleaning pipeline.

The false negative (*C. jejuni* gene inserted) may be explained by the extreme plasticity of Staphylococcus aureus genome and its propensity to integrate genes from other low-GC-content gram-positive bacteria as underlined in (9): “Gene transfer between Staphylococci and low-GC-content gram-positive bacteria appears to have shaped their virulence and resistance profiles”. If the *S. aureus* genome and the pangenome include genes from recent horizontal gene transfer from other low-GC-content gram-positive bacteria, wild genome vocabulary definition is less precise for machine learning and can lead to few false negative CDSs.

**8.7 List of the 219 genomes and plasmids used to make the *Campylobacter jejuni* pangenome**

| Organism/name | NCBI Accession number | Nb of genes | Size (Mb) | Level |
| --- | --- | --- | --- | --- |
| *Campylobacter jejuni* subsp. *jejuni* NCTC 11168 = ATCC 700819 | AL111168.1 | 1668 | 1.6 | complete |
| *Campylobacter jejuni* subsp. doylei strain NCTC11951 | LR134359.1 | 2052 | 1.9 | complete |
| *Campylobacter jejuni* subsp. *jejuni* strain ATCC 33560 | CP019838.1 | 1836 | 1.8 | complete |
| *Campylobacter jejuni* strain NCTC11351 | LN831025.1 | 1803 | 1.8 | complete |
| *Campylobacter jejuni* subsp. doylei 269.97 strain 269.97 | CP000768.1 | 1972 | 1.8 | complete |
| *Campylobacter jejuni* subsp. doylei strain FDAARGOS_295 | CP027403.1 | 1969 | 1.8 | complete |
| *Campylobacter jejuni* strain CFSAN054107 | CP028185.1 | 1999 | 1.9 | complete |
| *Campylobacter jejuni* strain CFSAN054107 plasmid pGMI16-002 | CP028186.1 | 81 | 0.1 | plasmid |
| *Campylobacter jejuni* strain NCTC13265 | LR134498.1 | 1948 | 1.8 | complete |
| *Campylobacter jejuni* strain HF5-4A-4 | CP007188.1 | 1971 | 1.8 | complete |
| *Campylobacter jejuni* subsp. *jejuni* strain NADC 20827 | CP045048.1 | 1956 | 1.9 | complete |
| *Campylobacter jejuni* subsp. *jejuni* strain NADC 20827 plasmid p20827L | CP045046.1 | 54 | 0 | plasmid |
| *Campylobacter jejuni* subsp. *jejuni* strain NADC 20827 plasmid p20827S | CP045047.1 | 5 | 0 | plasmid |
| *Campylobacter jejuni* strain NCTC13268 | LR134497.1 | 1864 | 1.8 | complete |
| *Campylobacter jejuni* subsp. *jejuni* R14 strain R14 | CP005081.1 | 1943 | 1.8 | complete |
| *Campylobacter jejuni* subsp. *jejuni* strain HPC5 | CP032316.1 | 1936 | 1.8 | complete |
| *Campylobacter jejuni* strain CJ018CCUA | CP012221.1 | 1926 | 1.8 | complete |
| *Campylobacter jejuni* subsp. *jejuni* strain MTVDSCj16 | CP017033.1 | 1866 | 1.8 | complete |
| *Campylobacter jejuni* RM1221 strain RM1221 | CP000025.1 | 1888 | 1.8 | complete |
| *Campylobacter jejuni* strain FDAARGOS_421 | CP023866.1 | 1887 | 1.8 | complete |
| *Campylobacter jejuni* strain YH002 | CP020776.1 | 1935 | 1.8 | complete |
| *Campylobacter jejuni* strain YH002 plasmid pCJP002 | CP020775.1 | 54 | 0 | plasmid |
| *Campylobacter jejuni* strain FDAARGOS_262 | CP022076.1 | 1824 | 1.8 | complete |
| *Campylobacter jejuni* strain ZP3204 | CP017856.1 | 1888 | 1.8 | complete |
| *Campylobacter jejuni* strain ZP3204 plasmid pCJDM204L | CP017854.1 | 45 | 0 | plasmid |
| *Campylobacter jejuni* strain ZP3204 plasmid pCJDM204S | CP017855.1 | 6 | 0 | plasmid |
| *Campylobacter jejuni* strain TS1218 | CP017860.1 | 1881 | 1.8 | complete |
| *Campylobacter jejuni* strain TS1218 plasmid pCJDM218 | CP017861.1 | 46 | 0 | plasmid |
| *Campylobacter jejuni* subsp. doylei strain NCTC11924 | LR134530.1 | 1845 | 1.8 | complete |
| *Campylobacter jejuni* strain AR-0412 | CP044173.1 | 1925 | 1.8 | complete |
| *Campylobacter jejuni* strain AR-0412 plasmid pAR-0412 | CP044174.1 | - | - | plasmid |
| *Campylobacter jejuni* strain CFSAN032806 | CP045789.1 | 1916 | 1.8 | complete |
| *Campylobacter jejuni* strain CFSAN032806 plasmid pCFSAN032806 | CP045790.1 | 63 | 0 | plasmid |
| *Campylobacter jejuni* subsp. *jejuni* M129 strain M129 | CP007749.1 | 1863 | 1.8 | complete |
| *Campylobacter jejuni* subsp. *jejuni* M129 strain M129 plasmid pTet-M129 | CP007750.1 | 51 | 0 | plasmid |
| *Campylobacter jejuni* subsp. *jejuni* strain CLB104 | CP034393.1 | 1855 | 1.8 | complete |
| *Campylobacter jejuni* strain YQ2210 | CP017859.1 | 1886 | 1.8 | complete |
| *Campylobacter jejuni* strain YQ2210 plasmid pCJDM210L | CP017857.1 | 45 | 0 | plasmid |
| *Campylobacter jejuni* strain YQ2210 plasmid pCJDM210S | CP017858.1 | 6 | 0 | plasmid |
| *Campylobacter jejuni* strain CJ071CC464 | CP012217.1 | 1851 | 1.7 | complete |
| *Campylobacter jejuni* subsp. *jejuni* strain 00-0949 | CP010301.1 | 1955 | 1.8 | complete |
| *Campylobacter jejuni* subsp. *jejuni* strain 00-0949 plasmid pTet | CP010302.1 | 52 | 0 | complete |
| *Campylobacter jejuni* subsp. *jejuni* strain 00-0949 plasmid pVir | CP010303.1 | 46 | 0 | complete |
| *Campylobacter jejuni* strain IF1100 | CP017863.1 | 1853 | 1.7 | complete |
| *Campylobacter jejuni* strain IF1100 plasmid pCJDM100 | CP017864.1 | 6 | 0 | plasmid |
| *Campylobacter jejuni* strain YH003 | CP041584.1 | 1813 | 1.7 | complete |
| *Campylobacter jejuni* subsp. *jejuni* strain 01-1512 | CP010072.1 | 1954 | 1.8 | complete |
| *Campylobacter jejuni* subsp. *jejuni* strain 01-1512 plasmid pCj1 | CP010073.1 | 51 | 0 | plasmid |
| *Campylobacter jejuni* subsp. *jejuni* strain 01-1512 plasmid pCj2 | CP010074.1 | 46 | 0 | plasmid |
| *Campylobacter jejuni* subsp. *jejuni* strain 00-1597 | CP010306.1 | 1801 | 1.7 | complete |
| *Campylobacter jejuni* strain SCJK2 | CP038862.1 | 1993 | 1.9 | complete |
| *Campylobacter jejuni* strain SCJK2 plasmid p2 | CP038864.1 | 49 | 0 | plasmid |
| *Campylobacter jejuni* strain SCJK2 plasmid unnamed1 | CP038863.1 | 101 | 0.1 | plasmid |
| *Campylobacter jejuni* subsp. doylei strain NCTC11925 | LS483295.1 | 1789 | 1.7 | complete |
| *Campylobacter jejuni* strain CFSAN032806 | CP023543.1 | 1870 | 1.8 | complete |
| *Campylobacter jejuni* strain CFSAN032806 plasmid pCFSAN032806 | CP023544.1 | 66 | 0 | plasmid |
| *Campylobacter jejuni* strain CJ067CC45 | CP012206.1 | 1823 | 1.7 | complete |
| *Campylobacter jejuni* subsp. *jejuni* 00-2544 strain 00-2544 | CP006709.2 | 1860 | 1.8 | complete |
| *Campylobacter jejuni* subsp. *jejuni* 00-2544 strain 00-2544 plasmid unnamed | CP006710.1 | 44 | 0 | plasmid |
| *Campylobacter jejuni* subsp. *jejuni* 00-2538 strain 00-2538 | CP006707.2 | 1805 | 1.7 | complete |
| *Campylobacter jejuni* subsp. *jejuni* 00-2425 strain 00-2425 | CP006729.2 | 1801 | 1.7 | complete |
| *Campylobacter jejuni* strain AR-0414 | CP044169.1 | 1778 | 1.7 | complete |
| *Campylobacter jejuni* subsp. *jejuni* strain YH001 | CP010058.1 | 1809 | 1.7 | complete |
| *Campylobacter jejuni* subsp. *jejuni* strain 14980A | CP017029.1 | 1799 | 1.8 | complete |
| *Campylobacter jejuni* subsp. *jejuni* strain 14980A plasmid pCJ14980A | CP017030.1 | 51 | 0 | plasmid |
| *Campylobacter jejuni* strain 12567 | CP028909.1 | 1774 | 1.7 | complete |
| *Campylobacter jejuni* 32488 strain 32488 | CP006006.1 | 1765 | 1.7 | complete |
| *Campylobacter jejuni* strain FJ3124 | CP017862.1 | 1777 | 1.7 | complete |
| *Campylobacter jejuni* strain CJ074CC443 | CP012216.1 | 1764 | 1.7 | complete |
| *Campylobacter jejuni* subsp. *jejuni* F38011 strain F38011 | CP006851.1 | 1773 | 1.7 | complete |
| *Campylobacter jejuni* strain FDAARGOS_422 | CP023867.1 | 1771 | 1.7 | complete |
| *Campylobacter jejuni* strain AR-0419 | CP044162.1 | 1765 | 1.7 | complete |
| *Campylobacter jejuni* subsp. *jejuni* strain MTVDSCj13 | CP017032.1 | 1746 | 1.7 | complete |
| *Campylobacter jejuni* strain NCTC 12660 | CP028910.1 | 1756 | 1.7 | complete |
| *Campylobacter jejuni* subsp. *jejuni* strain huA17 | CP028372.1 | 1910 | 1.8 | complete |
| *Campylobacter jejuni* subsp. *jejuni* strain huA17 plasmid unnamed1 | CP028373.1 | 152 | 0.1 | plasmid |
| *Campylobacter jejuni* strain WP2202 | CP014742.1 | 1880 | 1.8 | complete |
| *Campylobacter jejuni* strain WP2202 plasmid pCJDM202 | CP014743.1 | 116 | 0.1 | plasmid |
| *Campylobacter jejuni* subsp. *jejuni* S3 strain S3 | CP001960.1 | 1818 | 1.7 | complete |
| *Campylobacter jejuni* subsp. *jejuni* S3 strain S3 plasmid pTet | CP001961.1 | 50 | 0 | plasmid |
| *Campylobacter jejuni* strain CJ017CCUA | CP012212.1 | 1772 | 1.7 | complete |
| *Campylobacter jejuni* subsp. *jejuni* 00-2426 strain 00-2426 | CP006708.2 | 1750 | 1.7 | complete |
| *Campylobacter jejuni* strain CJ677CC520 | CP010501.1 | 1777 | 1.7 | complete |
| *Campylobacter jejuni* subsp. *jejuni* D42a strain D42a | CP007751.1 | 1809 | 1.7 | complete |
| *Campylobacter jejuni* subsp. *jejuni* D42a strain D42a plasmid pTet-D42a | CP007752.1 | 54 | 0 | plasmid |
| *Campylobacter jejuni* strain CJ677CC032 | CP010496.1 | 1770 | 1.7 | complete |
| *Campylobacter jejuni* strain CJ677CC538 | CP010495.1 | 1775 | 1.7 | complete |
| *Campylobacter jejuni* strain NS4-5-1 | CP007192.1 | 1723 | 1.7 | complete |
| *Campylobacter jejuni* subsp. *jejuni* strain RM1285 | CP015209.1 | 1744 | 1.7 | complete |
| *Campylobacter jejuni* strain OD267 | CP014744.1 | 1921 | 1.8 | complete |
| *Campylobacter jejuni* strain OD267 plasmid pCJDM67 L | CP014745.1 | 108 | 0.1 | plasmid |
| *Campylobacter jejuni* strain OD267 plasmid pCJDM67 S | CP014746.1 | 46 | 0 | plasmid |
| *Campylobacter jejuni* strain CJ677CC073 | CP010475.1 | 1773 | 1.7 | complete |
| *Campylobacter jejuni* strain CJ677CC014 | CP010502.1 | 1777 | 1.7 | complete |
| *Campylobacter jejuni* subsp. *jejuni* strain 00-6200 | CP010307.1 | 1743 | 1.7 | complete |
| *Campylobacter jejuni* strain NS4-1-1 | CP007191.1 | 1719 | 1.7 | complete |
| *Campylobacter jejuni* strain CJ677CC002 | CP010472.1 | 1773 | 1.7 | complete |
| *Campylobacter jejuni* 4031 strain 4031 | HG428754.1 | 1732 | 1.7 | complete |
| *Campylobacter jejuni* strain CJ677CC100 | CP010462.1 | 1769 | 1.7 | complete |
| *Campylobacter jejuni* strain CJ677CC521 | CP010476.1 | 1779 | 1.7 | complete |
| *Campylobacter jejuni* strain CJ677CC524 | CP010480.1 | 1773 | 1.7 | complete |
| *Campylobacter jejuni* strain CJ677CC526 | CP010477.1 | 1754 | 1.7 | complete |
| *Campylobacter jejuni* strain CJ677CC523 | CP010508.1 | 1765 | 1.7 | complete |
| *Campylobacter jejuni* strain NCTC13255 | LR134499.1 | 1740 | 1.7 | complete |
| *Campylobacter jejuni* subsp. *jejuni* str. RM3420 strain RM3420 | CP017456.1 | 1733 | 1.7 | complete |
| *Campylobacter jejuni* strain NCTC13261 | LR134500.1 | 1753 | 1.7 | complete |
| *Campylobacter jejuni* strain CJ677CC531 | CP010492.1 | 1767 | 1.7 | complete |
| *Campylobacter jejuni* strain CJ677CC036 | CP010479.1 | 1745 | 1.7 | complete |
| *Campylobacter jejuni* strain NCTC13266 | LR134496.1 | 1738 | 1.7 | complete |
| *Campylobacter jejuni* subsp. *jejuni* strain RM3196 | CP012690.1 | 1738 | 1.7 | complete |
| *Campylobacter jejuni* subsp. *jejuni* strain RM3197 | CP012689.1 | 1740 | 1.7 | complete |
| *Campylobacter jejuni* strain CJ677CC024 | CP010467.1 | 1745 | 1.7 | complete |
| *Campylobacter jejuni* strain RM1246-ERRC | CP022470.1 | 1781 | 1.7 | complete |
| *Campylobacter jejuni* strain RM1246-ERRC plasmid pRM1246_ERRC | CP022471.1 | 53 | 0 | plasmid |
| *Campylobacter jejuni* strain CJ066CC508 | CP012224.1 | 1713 | 1.7 | complete |
| *Campylobacter jejuni* strain CJ677CC533 | CP010458.1 | 1760 | 1.7 | complete |
| *Campylobacter jejuni* subsp. *jejuni* strain NCTC12109 | LR134505.1 | 1710 | 1.7 | complete |
| *Campylobacter jejuni* subsp. *jejuni* strain MTVDSCj07 | CP017031.1 | 1708 | 1.7 | complete |
| *Campylobacter jejuni* strain CJ515CC45 | CP012210.1 | 1741 | 1.7 | complete |
| *Campylobacter jejuni* subsp. *jejuni* strain MTVDSCj20 | CP008787.1 | 1711 | 1.7 | complete |
| *Campylobacter jejuni* strain RM3194 | CP014344.1 | 1782 | 1.7 | complete |
| *Campylobacter jejuni* strain RM3194 plasmid unnamed | CP014345.1 | 80 | 0.1 | plasmid |
| *Campylobacter jejuni* strain FDAARGOS_265 | CP022079.1 | 1761 | 1.7 | complete |
| *Campylobacter jejuni* strain FDAARGOS_265 plasmid unnamed1 | CP022078.1 | 54 | 0 | plasmid |
| *Campylobacter jejuni* strain CJ677CC530 | CP010489.1 | 1743 | 1.6 | complete |
| *Campylobacter jejuni* strain 11168H/araE | CP022559.1 | 1715 | 1.6 | complete |
| *Campylobacter jejuni* strain AR-0415 | CP044167.1 | 1757 | 1.7 | complete |
| *Campylobacter jejuni* strain AR-0415 plasmid pAR-0415 | CP044168.1 | - | - | plasmid |
| *Campylobacter jejuni* strain BfR-CA-14430 | CP043763.1 | 1746 | 1.7 | complete |
| *Campylobacter jejuni* strain BfR-CA-14430 plasmid pBfR-CA-14430 | CP043764.1 | 45 | 0 | plasmid |
| *Campylobacter jejuni* strain 11168H/lacY | CP022439.1 | 1709 | 1.6 | complete |
| *Campylobacter jejuni* strain CJ677CC528 | CP010500.1 | 1728 | 1.6 | complete |
| *Campylobacter jejuni* strain CJ677CC064 | CP010468.1 | 1717 | 1.6 | complete |
| *Campylobacter jejuni* strain CJ677CC062 | CP010493.1 | 1721 | 1.6 | complete |
| *Campylobacter jejuni* strain NS4-9-1 | CP007193.1 | 1700 | 1.6 | complete |
| *Campylobacter jejuni* strain CJ677CC527 | CP010506.1 | 1718 | 1.6 | complete |
| *Campylobacter jejuni* strain CJ677CC532 | CP010490.1 | 1717 | 1.6 | complete |
| *Campylobacter jejuni* strain CJ677CC519 | CP010471.1 | 1713 | 1.6 | complete |
| *Campylobacter jejuni* strain CJ677CC013 | CP010461.1 | 1718 | 1.6 | complete |
| *Campylobacter jejuni* strain CJ677CC522 | CP010463.1 | 1714 | 1.6 | complete |
| *Campylobacter jejuni* strain CJ677CC536 | CP010474.1 | 1715 | 1.6 | complete |
| *Campylobacter jejuni* strain CJ677CC525 | CP010469.1 | 1711 | 1.6 | complete |
| *Campylobacter jejuni* subsp. *jejuni* NCTC 11168-GSv strain NCTC 11168-rNRC | CP006689.1 | 1715 | 1.6 | complete |
| *Campylobacter jejuni* subsp. *jejuni* NCTC 11168-BN148 strain NCTC 11168-BN148 | HE978252.1 | 1708 | 1.6 | complete |
| *Campylobacter jejuni* subsp. *jejuni* NCTC 11168-K12E5 strain NCTC 11168-K12E5 | CP006685.1 | 1710 | 1.6 | complete |
| *Campylobacter jejuni* subsp. *jejuni* NCTC 11168-Kf1 strain NCTC 11168-Kf1 | CP006686.1 | 1710 | 1.6 | complete |
| *Campylobacter jejuni* subsp. *jejuni* NCTC 11168-mcK12E5 strain NCTC 11168-mcK12E5 | CP006687.1 | 1712 | 1.6 | complete |
| *Campylobacter jejuni* subsp. *jejuni* NCTC 11168-mfK12E5 strain NCTC 11168-mfK12E5 | CP006688.1 | 1711 | 1.6 | complete |
| *Campylobacter jejuni* strain FDAARGOS_263 | CP022077.1 | 1714 | 1.6 | complete |
| *Campylobacter jejuni* subsp. *jejuni* strain NCTC11168 | LS483362.1 | 1715 | 1.6 | complete |
| *Campylobacter jejuni* subsp. *jejuni* strain NCTC10983 | LR134511.1 | 1712 | 1.6 | complete |
| *Campylobacter jejuni* strain CJ677CC094 | CP010464.1 | 1714 | 1.6 | complete |
| *Campylobacter jejuni* strain CJ677CC040 | CP010510.1 | 1713 | 1.6 | complete |
| *Campylobacter jejuni* strain AR-0413 | CP044171.1 | 1819 | 1.7 | complete |
| *Campylobacter jejuni* strain AR-0413 plasmid pAR-0413-1 | CP044170.1 | - | - | plasmid |
| *Campylobacter jejuni* strain AR-0413 plasmid pAR-0413-2 | CP044172.1 | - | - | plasmid |
| *Campylobacter jejuni* strain CJ677CC016 | CP010481.1 | 1729 | 1.6 | complete |
| *Campylobacter jejuni* strain CJ677CC034 | CP010484.1 | 1723 | 1.6 | complete |
| *Campylobacter jejuni* strain CJ677CC540 | CP010509.1 | 1720 | 1.6 | complete |
| *Campylobacter jejuni* strain CJ677CC085 | CP010504.1 | 1722 | 1.6 | complete |
| *Campylobacter jejuni* strain CJ677CC539 | CP010457.1 | 1729 | 1.6 | complete |
| *Campylobacter jejuni* strain CJ677CC041 | CP010482.1 | 1721 | 1.6 | complete |
| *Campylobacter jejuni* subsp. *jejuni* CG8421 strain CG8421 | CP005388.1 | 1715 | 1.6 | complete |
| *Campylobacter jejuni* strain RM1285 | CP012696.1 | 1700 | 1.6 | complete |
| *Campylobacter jejuni* strain CJ677CC026 | CP010470.1 | 1725 | 1.6 | complete |
| *Campylobacter jejuni* strain CJ677CC039 | CP010503.1 | 1722 | 1.6 | complete |
| *Campylobacter jejuni* subsp. *jejuni* PT14 strain PT14 | CP003871.4 | 1694 | 1.6 | complete |
| *Campylobacter jejuni* strain CJ677CC047 | CP010459.1 | 1720 | 1.6 | complete |
| *Campylobacter jejuni* strain CJ677CC052 | CP010505.1 | 1717 | 1.6 | complete |
| *Campylobacter jejuni* strain CJ677CC061 | CP010511.1 | 1720 | 1.6 | complete |
| *Campylobacter jejuni* subsp. *jejuni* IA3902 strain IA3902 | CP001876.1 | 1749 | 1.7 | complete |
| *Campylobacter jejuni* subsp. *jejuni* IA3902 strain IA3902 plasmid pVir | CP001877.1 | 53 | 0 | plasmid |
| *Campylobacter jejuni* strain NCTC12851 | LR134507.1 | 1685 | 1.6 | complete |
| *Campylobacter jejuni* strain CJ031CC45 | CP012211.1 | 1723 | 1.6 | complete |
| *Campylobacter jejuni* strain NCTC 12664 | CP028912.1 | 1694 | 1.6 | complete |
| *Campylobacter jejuni* strain CJ677CC541 | CP010466.1 | 1713 | 1.6 | complete |
| *Campylobacter jejuni* strain CJ677CC033 | CP010497.1 | 1718 | 1.6 | complete |
| *Campylobacter jejuni* strain NCTC13257 | LR134502.1 | 1684 | 1.6 | complete |
| *Campylobacter jejuni* strain CJ677CC012 | CP010487.1 | 1717 | 1.6 | complete |
| *Campylobacter jejuni* strain CJ677CC534 | CP010473.1 | 1719 | 1.6 | complete |
| *Campylobacter jejuni* strain CJ677CC535 | CP010483.1 | 1723 | 1.6 | complete |
| *Campylobacter jejuni* strain CJ677CC542 | CP010499.1 | 1717 | 1.6 | complete |
| *Campylobacter jejuni* strain CJ677CC537 | CP010498.1 | 1720 | 1.6 | complete |
| *Campylobacter jejuni* strain CJ677CC092 | CP010488.1 | 1717 | 1.6 | complete |
| *Campylobacter jejuni* strain CJ677CC058 | CP010460.1 | 1720 | 1.6 | complete |
| *Campylobacter jejuni* strain CJ677CC059 | CP010494.1 | 1714 | 1.6 | complete |
| *Campylobacter jejuni* strain CJ677CC078 | CP010507.1 | 1715 | 1.6 | complete |
| *Campylobacter jejuni* strain CJ677CC529 | CP010491.1 | 1715 | 1.6 | complete |
| *Campylobacter jejuni* strain CJ677CC086 | CP010485.1 | 1711 | 1.6 | complete |
| *Campylobacter jejuni* strain FORC_046 | CP017229.1 | 1775 | 1.7 | complete |
| *Campylobacter jejuni* strain FORC_046 plasmid pFORC46.1 | CP017230.1 | 62 | 0 | plasmid |
| *Campylobacter jejuni* strain FORC_046 plasmid pFORC46.2 | CP017231.1 | 41 | 0 | plasmid |
| *Campylobacter jejuni* strain HF5-7-1 | CP007190.1 | 1671 | 1.6 | complete |
| *Campylobacter jejuni* strain HF5-5-1 | CP007189.1 | 1674 | 1.6 | complete |
| *Campylobacter jejuni* strain CJ677CC095 | CP010486.1 | 1713 | 1.6 | complete |
| *Campylobacter jejuni* subsp. *jejuni* 81116 strain 81116; NCTC 11828 | CP000814.1 | 1677 | 1.6 | complete |
| *Campylobacter jejuni* strain FORC_083 | CP028933.1 | 1747 | 1.7 | complete |
| *Campylobacter jejuni* strain FORC_083 plasmid pFORC_083_2 | CP028934.1 | 70 | 0 | plasmid |
| *Campylobacter jejuni* strain FORC_083 plasmid pFORC_083_3 | CP028935.1 | 1 | 0 | plasmid |
| *Campylobacter jejuni* strain CJ677CC008 | CP010465.1 | 1707 | 1.6 | complete |
| *Campylobacter jejuni* strain FDAARGOS_266 | CP022080.1 | 1673 | 1.6 | complete |
| *Campylobacter jejuni* strain 81-176_G1_B0 | CP022440.1 | 1667 | 1.6 | complete |
| *Campylobacter jejuni* subsp. *jejuni* strain ATCC 35925 | CP020045.1 | 1654 | 1.6 | complete |
| *Campylobacter jejuni* strain CJM1cam | CP012149.1 | 1667 | 1.6 | complete |
| *Campylobacter jejuni* subsp. *jejuni* M1 strain M1 | CP001900.1 | 1667 | 1.6 | complete |
| *Campylobacter jejuni* subsp. *jejuni* 81-176 strain 81-176 | CP000538.1 | 1771 | 1.7 | complete |
| *Campylobacter jejuni* subsp. *jejuni* 81-176 strain 81-176 plasmid pTet | CP000549.1 | 52 | 0 | plasmid |
| *Campylobacter jejuni* subsp. *jejuni* 81-176 strain 81-176 plasmid pVir | CP000550.1 | 53 | 0 | plasmid |
| *Campylobacter jejuni* strain 81-176_G1_B7 | CP022551.1 | 1671 | 1.6 | complete |
| *Campylobacter jejuni* strain NCTC 12661 | CP028911.1 | 1656 | 1.6 | complete |
| *Campylobacter jejuni* strain CJ513CC45 | CP012213.1 | 1685 | 1.6 | complete |
| *Campylobacter jejuni* strain NCTC12662 | CP019965.1 | 1657 | 1.6 | complete |
| *Campylobacter jejuni* subsp. *jejuni* strain 35925 | CP010906.1 | 1652 | 1.6 | complete |
| *Campylobacter jejuni* strain BC | CP032522.1 | 1680 | 1.6 | complete |
| *Campylobacter jejuni* strain CJ088CC52 | CP012214.1 | 1674 | 1.6 | complete |
| *Campylobacter jejuni* strain CJ090CC1332 | CP012220.1 | 1638 | 1.6 | complete |
| *Campylobacter jejuni* subsp. *jejuni* ICDCCJ07001 strain ICDCCJ07001 | CP002029.1 | 1669 | 1.7 | complete |
| *Campylobacter jejuni* subsp. *jejuni* ICDCCJ07001 strain ICDCCJ07001 plasmid pTet | CP002030.1 | 37 | 0 | plasmid |
| *Campylobacter jejuni* strain CJ677CC010 | CP010478.1 | 1751 | 1.7 | complete |

**8.8 List of the 188 genomes and plasmids used to make the *Lactococcus lactis* pangenome**

| Organism/name | NCBI Accession number | Nb of genes | Size (Mb) | Level |
| --- | --- | --- | --- | --- |
| *Lactococcus lactis* subsp. *lactis* strain A12 | LT599049.1 | 2700 | 2.7 | complete |
| *Lactococcus lactis* subsp. *lactis* strain A12 plasmid pA12-1 | LT599050.1 | 6 | 0 | plasmid |
| *Lactococcus lactis* subsp. *lactis* strain A12 plasmid pA12-2 | LT599051.1 | 8 | 0 | plasmid |
| *Lactococcus lactis* subsp. *lactis* strain A12 plasmid pA12-3 | LT599052.1 | 58 | 0 | plasmid |
| *Lactococcus lactis* subsp. *lactis* strain A12 plasmid pA12-4 | LT599053.1 | 85 | 0.1 | plasmid |
| *Lactococcus lactis* subsp. *lactis* strain KF147 | CP001834.1 | 2616 | 2.6 | complete |
| *Lactococcus lactis* subsp. *lactis* strain KF147 plasmid pKF147A | CP001835.1 | 38 | 0 | plasmid |
| *Lactococcus lactis* subsp. *lactis* strain 14B4 | CP028160.1 | 2626 | 2.6 | complete |
| *Lactococcus lactis* subsp. *lactis* strain 14B4 plasmid p14B4 | CP028161.1 | 63 | 0 | plasmid |
| *Lactococcus lactis* subsp. *lactis* strain UC06 | CP015902.1 | 2714 | 2.7 | complete |
| *Lactococcus lactis* subsp. *lactis* strain UC06 plasmid pUC06A | CP016734.1 | 43 | 0 | plasmid |
| *Lactococcus lactis* subsp. *lactis* strain UC06 plasmid pUC06B | CP016735.1 | 55 | 0 | plasmid |
| *Lactococcus lactis* subsp. *lactis* strain UC06 plasmid pUC06C | CP016736.1 | 29 | 0 | plasmid |
| *Lactococcus lactis* subsp. *lactis* strain UC06 plasmid pUC06D | CP034579.1 | 10 | 0 | plasmid |
| *Lactococcus lactis* subsp. *lactis* strain UC06 plasmid pUC06E | CP034580.1 | 6 | 0 | plasmid |
| *Lactococcus lactis* subsp. *lactis* strain UC06 plasmid pUC06F | CP034581.1 | 27 | 0 | plasmid |
| *Lactococcus lactis* subsp. *lactis* strain NCDO 2118 | CP009054.1 | 2551 | 2.6 | complete |
| *Lactococcus lactis* subsp. *lactis* strain NCDO 2118 plasmid pNCDO2118 | CP009055.1 | 44 | 0 | plasmid |
| *Lactococcus lactis* subsp. *lactis* strain 275 | CP015897.1 | 2784 | 2.8 | complete |
| *Lactococcus lactis* subsp. *lactis* strain 275 plasmid p275A | CP016699.1 | 104 | 0.1 | plasmid |
| *Lactococcus lactis* subsp. *lactis* strain 275 plasmid p275B | CP016700.1 | 65 | 0 | plasmid |
| *Lactococcus lactis* subsp. *lactis* strain 275 plasmid p275C | CP016701.1 | 62 | 0 | plasmid |
| *Lactococcus lactis* subsp. *lactis* strain 275 plasmid p275D | CP016702.1 | 60 | 0 | plasmid |
| *Lactococcus lactis* subsp. *lactis* strain S0 | CP010050.1 | 2493 | 2.5 | complete |
| *Lactococcus lactis* subsp. *lactis* strain 229 | CP015896.1 | 2663 | 2.6 | complete |
| *Lactococcus lactis* subsp. *lactis* strain 229 plasmid p229A | CP016694.1 | 59 | 0 | plasmid |
| *Lactococcus lactis* subsp. *lactis* strain 229 plasmid p229B | CP016695.1 | 29 | 0 | plasmid |
| *Lactococcus lactis* subsp. *lactis* strain 229 plasmid p229C | CP016696.1 | 29 | 0 | plasmid |
| *Lactococcus lactis* subsp. *lactis* strain 229 plasmid p229D | CP016697.1 | 8 | 0 | plasmid |
| *Lactococcus lactis* subsp. *lactis* strain 229 plasmid p229E | CP016698.1 | 51 | 0 | plasmid |
| *Lactococcus lactis* subsp. *lactis* strain F44 | CP024954.1 | 2350 | 2.4 | complete |
| *Lactococcus lactis* subsp. *lactis* bv. diacety*lactis* strain FM03 | CP020604.1 | 2560 | 2.5 | complete |
| *Lactococcus lactis* subsp. *lactis* bv. diacety*lactis* strain FM03 plasmid pLd1 | CP020605.1 | 10 | 0 | plasmid |
| *Lactococcus lactis* subsp. *lactis* bv. diacety*lactis* strain FM03 plasmid pLd2 | CP020606.1 | 16 | 0 | plasmid |
| *Lactococcus lactis* subsp. *lactis* bv. diacety*lactis* strain FM03 plasmid pLd3 | CP020607.1 | 2 | 0 | plasmid |
| *Lactococcus lactis* subsp. *lactis* bv. diacety*lactis* strain FM03 plasmid pLd4 | CP020608.1 | 11 | 0 | plasmid |
| *Lactococcus lactis* subsp. *lactis* bv. diacety*lactis* strain FM03 plasmid pLd5 | CP020609.1 | 6 | 0 | plasmid |
| *Lactococcus lactis* subsp. *lactis* bv. diacety*lactis* strain FM03 plasmid pLd6 | CP020610.1 | 3 | 0 | plasmid |
| *Lactococcus lactis* subsp. *lactis* bv. diacety*lactis* strain FM03 plasmid pLd7 | CP020611.1 | 30 | 0 | plasmid |
| *Lactococcus lactis* subsp. *lactis* strain UL8 | CP015908.1 | 2495 | 2.5 | complete |
| *Lactococcus lactis* subsp. *lactis* strain UL8 plasmid pUL8A | CP016704.1 | 6 | 0 | plasmid |
| *Lactococcus lactis* subsp. *lactis* strain UL8 plasmid pUL8B | CP016705.1 | 30 | 0 | plasmid |
| *Lactococcus lactis* subsp. *lactis* strain UL8 plasmid pUL8C | CP016706.1 | 3 | 0 | plasmid |
| *Lactococcus lactis* subsp. *lactis* IO-1 strain IO-1 | AP012281.1 | 2329 | 2.4 | complete |
| *Lactococcus lactis* subsp. *lactis* bv. diacety*lactis* strain SD96 | CP043523.1 | 2706 | 2.6 | complete |
| *Lactococcus lactis* subsp. *lactis* bv. diacety*lactis* strain SD96 plasmid pSD96_01 | CP043525.1 | 15 | 0 | plasmid |
| *Lactococcus lactis* subsp. *lactis* bv. diacety*lactis* strain SD96 plasmid pSD96_02 | CP043526.1 | 91 | 0.1 | plasmid |
| *Lactococcus lactis* subsp. *lactis* bv. diacety*lactis* strain SD96 plasmid pSD96_03 | CP043527.1 | 35 | 0 | plasmid |
| *Lactococcus lactis* subsp. *lactis* bv. diacety*lactis* strain SD96 plasmid pSD96_04 | CP043528.1 | 12 | 0 | plasmid |
| *Lactococcus lactis* subsp. *lactis* bv. diacety*lactis* strain SD96 plasmid pSD96_05 | CP043524.1 | 56 | 0 | plasmid |
| *Lactococcus lactis* subsp. *lactis* bv. diacety*lactis* strain SD96 plasmid pSD96_06 | CP043518.1 | 26 | 0 | plasmid |
| *Lactococcus lactis* subsp. *lactis* bv. diacety*lactis* strain SD96 plasmid pSD96_07 | CP043519.1 | 12 | 0 | plasmid |
| *Lactococcus lactis* subsp. *lactis* bv. diacety*lactis* strain SD96 plasmid pSD96_08 | CP043520.1 | 10 | 0 | plasmid |
| *Lactococcus lactis* subsp. *lactis* bv. diacety*lactis* strain SD96 plasmid pSD96_09 | CP043521.1 | 5 | 0 | plasmid |
| *Lactococcus lactis* subsp. *lactis* bv. diacety*lactis* strain SD96 plasmid pSD96_10 | CP043522.1 | 17 | 0 | plasmid |
| *Lactococcus lactis* subsp. *lactis* strain G423 | CP024958.1 | 2345 | 2.4 | complete |
| *Lactococcus lactis* subsp. *lactis* strain CV56 | CP002365.1 | 2516 | 2.5 | complete |
| *Lactococcus lactis* subsp. *lactis* strain CV56 plasmid pCV56A | CP002366.1 | 39 | 0 | plasmid |
| *Lactococcus lactis* subsp. *lactis* strain CV56 plasmid pCV56B | CP002367.1 | 28 | 0 | plasmid |
| *Lactococcus lactis* subsp. *lactis* strain CV56 plasmid pCV56C | CP002368.1 | 29 | 0 | plasmid |
| *Lactococcus lactis* subsp. *lactis* strain CV56 plasmid pCV56D | CP002369.1 | 8 | 0 | plasmid |
| *Lactococcus lactis* subsp. *lactis* strain CV56 plasmid pCV56E | CP002370.1 | 3 | 0 | plasmid |
| *Lactococcus lactis* subsp. *lactis* strain UC063 | CP015905.1 | 2570 | 2.5 | complete |
| *Lactococcus lactis* subsp. *lactis* strain UC063 plasmid pUC063A | CP016715.1 | 79 | 0.1 | plasmid |
| *Lactococcus lactis* subsp. *lactis* strain UC063 plasmid pUC063B | CP016716.1 | 41 | 0 | plasmid |
| *Lactococcus lactis* subsp. *lactis* strain UC063 plasmid pUC063C | CP016717.1 | 15 | 0 | plasmid |
| *Lactococcus lactis* subsp. *lactis* strain UC063 plasmid pUC063D | CP016718.1 | 10 | 0 | plasmid |
| *Lactococcus lactis* subsp. *lactis* strain UC063 plasmid pUC063E | CP016719.1 | 11 | 0 | plasmid |
| *Lactococcus lactis* subsp. *lactis* strain UC11 | CP015904.1 | 2483 | 2.5 | complete |
| *Lactococcus lactis* subsp. *lactis* strain UC11 plasmid pCU11E | CP034572.1 | 8 | 0 | plasmid |
| *Lactococcus lactis* subsp. *lactis* strain UC11 plasmid pUC11A | CP016720.1 | 65 | 0 | plasmid |
| *Lactococcus lactis* subsp. *lactis* strain UC11 plasmid pUC11B | CP016721.1 | 52 | 0 | plasmid |
| *Lactococcus lactis* subsp. *lactis* strain UC11 plasmid pUC11C | CP016722.1 | 18 | 0 | plasmid |
| *Lactococcus lactis* subsp. *lactis* strain UC11 plasmid pUC11D | CP016723.1 | 17 | 0 | plasmid |
| *Lactococcus lactis* subsp. *lactis* strain UC11 plasmid pUC11F | CP016725.1 | 4 | 0 | plasmid |
| *Lactococcus lactis* subsp. *lactis* strain UC08 | CP015903.1 | 2502 | 2.5 | complete |
| *Lactococcus lactis* subsp. *lactis* strain UC08 plasmid pUC08A | CP016726.1 | 102 | 0.1 | plasmid |
| *Lactococcus lactis* subsp. *lactis* strain UC08 plasmid pUC08B | CP016727.1 | 52 | 0 | plasmid |
| *Lactococcus lactis* subsp. *lactis* strain UC08 plasmid pUC08C | CP016728.1 | 21 | 0 | plasmid |
| *Lactococcus lactis* subsp. *lactis* strain UC08 plasmid pUC08D | CP034577.1 | 4 | 0 | plasmid |
| *Lactococcus lactis* subsp. *lactis* strain UC08 plasmid pUC08E | CP034578.1 | 7 | 0 | plasmid |
| *Lactococcus lactis* subsp. *lactis* Il1403 strain IL1403 | AE005176.1 | 2406 | 2.4 | complete |
| *Lactococcus lactis* subsp. *lactis* strain G50 | CP025500.1 | 2300 | 2.3 | complete |
| *Lactococcus lactis* subsp. *Lactis* strain KLDS 4.0325 | CP006766.1 | 2869 | 2.8 | complete |
| *Lactococcus lactis* subsp. *Lactis* strain KLDS 4.0325 plasmid 1 | CP006767.1 | 5 | 0 | plasmid |
| *Lactococcus lactis* subsp. *Lactis* strain KLDS 4.0325 plasmid 2 | CP007042.1 | 2 | 0 | plasmid |
| *Lactococcus lactis* subsp. *Lactis* strain KLDS 4.0325 plasmid 3 | CP007043.1 | 3 | 0 | plasmid |
| *Lactococcus lactis* subsp. *Lactis* strain KLDS 4.0325 plasmid unnamed4 | CP029291.1 | 14 | 0 | plasmid |
| *Lactococcus lactis* subsp. *Lactis* strain KLDS 4.0325 plasmid unnamed5 | CP029292.1 | 63 | 0 | plasmid |
| *Lactococcus lactis* subsp. *Lactis* strain KLDS 4.0325 plasmid unnamed6 | CP029293.1 | 119 | 0.1 | plasmid |
| *Lactococcus lactis* subsp. *lactis* strain UC77 | CP015906.1 | 2825 | 2.7 | complete |
| *Lactococcus lactis* subsp. *lactis* strain UC77 plasmid pUC77A | CP016713.1 | 7 | 0 | plasmid |
| *Lactococcus lactis* subsp. *lactis* strain UC77 plasmid pUC77B | CP016714.1 | 66 | 0 | plasmid |
| *Lactococcus lactis* subsp. *lactis* strain UC77 plasmid pUC77C | CP034573.1 | 58 | 0 | plasmid |
| *Lactococcus lactis* subsp. *lactis* strain UC77 plasmid pUC77D | CP034574.1 | 47 | 0 | plasmid |
| *Lactococcus lactis* subsp. *lactis* strain UC77 plasmid pUC77E | CP034575.1 | 7 | 0 | plasmid |
| *Lactococcus lactis* subsp. *lactis* strain 184 | CP015895.1 | 2441 | 2.4 | complete |
| *Lactococcus lactis* subsp. *lactis* strain 184 plasmid p184A | CP016691.1 | 13 | 0 | plasmid |
| *Lactococcus lactis* subsp. *lactis* strain 184 plasmid p184B | CP016692.1 | 6 | 0 | plasmid |
| *Lactococcus lactis* subsp. *lactis* strain 184 plasmid p184C | CP016693.1 | 14 | 0 | plasmid |
| *Lactococcus lactis* subsp. *lactis* strain 184 plasmid p184D | CP034584.1 | 3 | 0 | plasmid |
| *Lactococcus lactis* subsp. *lactis* strain 184 plasmid p184E | CP034585.1 | 4 | 0 | plasmid |
| *Lactococcus lactis* subsp. *lactis* strain 184 plasmid p184F | CP034586.1 | 8 | 0 | plasmid |
| *Lactococcus lactis* subsp. *lactis* strain C10 | CP015898.1 | 2437 | 2.4 | complete |
| *Lactococcus lactis* subsp. *lactis* strain C10 plasmid pC10A | CP016703.1 | 4 | 0 | plasmid |
| *Lactococcus lactis* subsp. *lactis* strain C10 plasmid pC10B | CP034582.1 | 48 | 0 | plasmid |
| *Lactococcus lactis* subsp. *lactis* strain C10 plasmid pC10C | CP034583.1 | 5 | 0 | plasmid |

**8.9 List of the 48 genomes and plasmids used to make the *listeria monocytogenes* pangenome (partially adapted from (5))**

| Organism/name | NCBI Accession number | Nb of genes | Size (Mb) | Level |
| --- | --- | --- | --- | --- |
| *Listeria monocytogenes* stain J1816 | CP006047.2 | 2947 | 2.9 | complete |
| *Listeria monocytogenes* strain C1-387 | CP006591.1 | 3052 | 3.0 | complete |
| *Listeria monocytogenes* strain J2-064 | CP006592.1 | 2946 | 2.9 | complete |
| *Listeria monocytogenes* strain J2-031 | CP006593.1 | 3020 | 3.0 | complete |
| *Listeria monocytogenes* strain R2-502 | CP006594.1 | 3142 | 3.0 | complete |
| *Listeria monocytogenes* strain J1-108 | CP006596.2 | 2981 | 2.9 | complete |
| *Listeria monocytogenes* strain N1-011A | CP006597.1 | 3160 | 3.1 | complete |
| *Listeria monocytogenes* strain J1776 | CP006598.1 | 2996 | 3.0 | complete |
| *Listeria monocytogenes* strain J1817 | CP006599.1 | 2998 | 3.0 | complete |
| *Listeria monocytogenes* strain J1926 | CP006600.1 | 2995 | 3.0 | complete |
| *Listeria monocytogenes* strain CFSAN023459 | CP014252.1 | 3113 | 3.0 | complete |
| *Listeria monocytogenes* strain CFSAN023459 plasmid CFSAN023459_01 | CP014253.1 | 17 | 0 | plasmid |
| *Listeria monocytogenes* strain CFSAN023459 plasmid CFSAN023459_02 | CP014254.1 | 62 | 0 | plasmid |
| *Listeria monocytogenes* strain FDA00006907 | CP022020.1 | 3066 | 3.0 | complete |
| *Listeria monocytogenes* strain FDA00006907 plasmid pCFSAN021445 | CP022021.1 | 168 | 0.1 | plasmid |
| *Listeria monocytogenes* strain AUSMDU00000224 | CP045972.1 | 2851 | 2.8 | complete |
| *Listeria monocytogenes* strain AUSMDU00000224 plasmid pAUSMDU00000224_01 | CP045973.1 | 62 | 0 | plasmid |
| *Listeria monocytogenes* serotype 1-2c str. SLCC2372 plasmid pLM1-2cUG1 | FR667691.1 | 54 | 0 | plasmid |
| *Listeria monocytogenes* strain SLCC2372 serotype 1/2c | FR733648.1 | 2936 | 2.9 | complete |
| *Listeria monocytogenes* R479a | HG813247.1 | 2982 | 2.9 | complete |
| *Listeria monocytogenes* R479a plasmid pLMR479a | HG813248.1 | 92 | 0.1 | plasmid |
| *Listeria monocytogenes* serotype 4b str. F2365 | NC_002973.6 | 2883 | 2.9 | complete |
| *Listeria monocytogenes* EGD-e chromosome | NC_003210.1 | 2867 | 2.8 | complete |
| *Listeria monocytogenes* HCC23 | NC_011660.1 | 3031 | 3.0 | complete |
| *Listeria monocytogenes* serotype 4b str. CLIP 80459 | NC_012488.1 | 2882 | 2.9 | complete |
| *Listeria monocytogenes* strain 08-5578 | NC_013766.2 | 3144 | 3.1 | complete |
| *Listeria monocytogenes* strain 08-5923 | NC_013768.1 | 3024 | 3.0 | complete |
| *Listeria monocytogenes* strain L99 | NC_017529.1 | 3027 | 3.0 | complete |
| *Listeria monocytogenes* strain M7 | NC_017537.1 | 3031 | 3.0 | complete |
| *Listeria monocytogenes* strain 10403S | NC_017544.1 | 2955 | 2.9 | complete |
| *Listeria monocytogenes* strain J0161 | NC_017545.1 | 3077 | 3.0 | complete |
| *Listeria monocytogenes* strain FSL R2-561 | NC_017546.1 | 2997 | 3.0 | complete |
| *Listeria monocytogenes* strain Finland 1998 | NC_017547.1 | 2902 | 2.9 | complete |
| *Listeria monocytogenes* strain 07PF0776 | NC_017728.1 | 2939 | 2.9 | complete |
| *Listeria monocytogenes* strain ATCC 19117 serotype 4d | NC_018584.1 | 2976 | 2.9 | complete |
| *Listeria monocytogenes* strain SLCC2378 | NC_018585.1 | 2919 | 2.9 | complete |
| *Listeria monocytogenes* strain SLCC2540 | NC_018586.1 | 2951 | 2.9 | complete |
| *Listeria monocytogenes* strain SLCC2755 | NC_018587.1 | 3045 | 3.0 | complete |
| *Listeria monocytogenes* strain SLCC2372 | NC_018588.1 | 3053 | 3.0 | complete |
| *Listeria monocytogenes* strain SLCC2479 | NC_018589.1 | 2997 | 3.0 | complete |
| *Listeria monocytogenes* strain SLCC2376 | NC_018590.1 | 2810 | 2.8 | complete |
| *Listeria monocytogenes* serotype 7 str. SLCC2482 | NC_018591.1 | 2968 | 2.9 | complete |
| *Listeria monocytogenes* strain SLCC5850 | NC_018592.1 | 2911 | 2.9 | complete |
| *Listeria monocytogenes* strain SLCC7179 | NC_018593.1 | 2896 | 2.9 | complete |
| *Listeria monocytogenes* strain L312 | NC_018642.1 | 2880 | 2.9 | complete |
| *Listeria monocytogenes* serotype 4b str. LL195 | NC_019556.1 | 2936 | 2.9 | complete |
| *Listeria monocytogenes* strain La111 | NC_020557.1 | 2884 | 2.9 | complete |
| *Listeria monocytogenes* strain N53-1 | NC_020558.1 | 2894 | 2.9 | complete |

**8.10 List of the 49 genomes and plasmids used to make the *Staphylococcus aureus* pangenome** **(partially adapted from (6))**

| Organism/name | NCBI Accession number | Nb of genes | Size (Mb) | Level |
| --- | --- | --- | --- | --- |
| *Staphylococcus aureus* subsp. *aureus* Mu3 DNA | AP009324.1 | 2699 | 2.7 | complete |
| *Staphylococcus aureus* strain IT4-R | CP028470.1 | 2895 | 2.9 | complete |
| *Staphylococcus aureus* subsp. *aureus* N315 | NC_002745.2 | 2812 | 2.8 | complete |
| *Staphylococcus aureus* subsp. *aureus* Mu50 | NC_002758.2 | 2907 | 2.9 | complete |
| *Staphylococcus aureus* subsp. *aureus* COL | NC_002951.2 | 2862 | 2.9 | complete |
| *Staphylococcus aureus* subsp. *aureus* MRSA252 | NC_002952.2 | 2873 | 2.9 | complete |
| *Staphylococcus aureus* subsp. *aureus* MSSA476 | NC_002953.3 | 2785 | 2.8 | complete |
| *Staphylococcus aureus* subsp. *aureus* MW2 | NC_003923.1 | 2783 | 2.8 | complete |
| *Staphylococcus aureus* RF122 | NC_007622.1 | 2730 | 2.7 | complete |
| *Staphylococcus aureus* subsp. *aureus* USA300_FPR3757 | NC_007793.1 | 2990 | 3.0 | complete |
| *Staphylococcus aureus* subsp. *aureus* NCTC 8325 | NC_007795.1 | 3148 | 3.1 | complete |
| *Staphylococcus aureus* subsp. *aureus* JH9 | NC_009487.1 | 3022 | 3.0 | complete |
| *Staphylococcus aureus* subsp. *aureus* JH1 | NC_009632.1 | 3023 | 3.0 | complete |
| *Staphylococcus aureus* subsp. *aureus* str. Newman | NC_009641.1 | 2933 | 3.0 | complete |
| *Staphylococcus aureus* subsp. *aureus* Mu3 | NC_009782.1 | 2880 | 3.0 | complete |
| *Staphylococcus aureus* subsp. *aureus* USA300_TCH1516 | NC_010079.1 | 2997 | 3.0 | complete |
| *Staphylococcus aureus* subsp. *aureus* ED98 | NC_013450.1 | 2840 | 2.8 | complete |
| *Staphylococcus aureus* subsp. *aureus* VC40 | NC_016912.1 | 2658 | 2.6 | complete |
| *Staphylococcus aureus* subsp. *aureus* M013 | NC_016928.2 | 2766 | 2.7 | complete |
| *Staphylococcus aureus* subsp. *aureus* TW20 | NC_017331.1 | 3112 | 3.2 | complete |
| *Staphylococcus aureus* subsp. *aureus* ST398 | NC_017333.1 | 2858 | 2.8 | complete |
| *Staphylococcus aureus* subsp. *aureus* ED133 | NC_017337.1 | 2886 | 2.9 | complete |
| *Staphylococcus aureus* subsp. *aureus* JKD6159 | NC_017338.1 | 2798 | 2.8 | complete |
| *Staphylococcus aureus* 04-02981 | NC_017340.1 | 2862 | 2.9 | complete |
| *Staphylococcus aureus* subsp. *aureus* str. JKD6008 | NC_017341.1 | 3038 | 3.0 | complete |
| *Staphylococcus aureus* subsp. *aureus* TCH60 | NC_017342.1 | 2778 | 2.8 | complete |
| *Staphylococcus aureus* subsp. *aureus* ECT-R 2 | NC_017343.1 | 2722 | 2.7 | complete |
| *Staphylococcus aureus* subsp. *aureus* T0131 | NC_017347.1 | 2911 | 2.9 | complete |
| *Staphylococcus aureus* subsp. *aureus* LGA251 | NC_017349.1 | 2684 | 2.7 | complete |
| *Staphylococcus aureus* subsp. *aureus* 11819-97 | NC_017351.1 | 2864 | 2.8 | complete |
| *Staphylococcus aureus* subsp. *aureus* 71193 | NC_017673.1 | 2646 | 2.6 | complete |
| *Staphylococcus aureus* subsp. *aureus* HO 5096 0412 | NC_017763.1 | 2809 | 2.8 | complete |
| *Staphylococcus aureus* 08BA02176 | NC_018608.1 | 2782 | 2.8 | complete |
| *Staphylococcus aureus* subsp. *aureus* ST228 | NC_020529.1 | 2759 | 2.7 | complete |
| *Staphylococcus aureus* subsp. *aureus* ST228 | NC_020532.1 | 2762 | 2.7 | complete |
| *Staphylococcus aureus* subsp. *aureus* ST228 | NC_020533.1 | 2761 | 2.7 | complete |
| *Staphylococcus aureus* subsp. *aureus* ST228 | NC_020536.1 | 2762 | 2.7 | complete |
| *Staphylococcus aureus* subsp. *aureus* ST228 | NC_020537.1 | 2753 | 2.7 | complete |
| *Staphylococcus aureus* subsp. *aureus* ST228 | NC_020564.1 | 2759 | 2.7 | complete |
| *Staphylococcus aureus* subsp. *aureus* ST228 | NC_020566.1 | 2765 | 2.7 | complete |
| *Staphylococcus aureus* subsp. *aureus* ST228 | NC_020568.1 | 2755 | 2.è | complete |
| *Staphylococcus aureus* M1 | NC_021059.1 | 2907 | 2.9 | complete |
| *Staphylococcus aureus* CA-347 | NC_021554.1 | 2930 | 2.9 | complete |
| *Staphylococcus aureus* | NC_021670.1 | 3026 | 3.0 | complete |
| *Staphylococcus aureus* subsp. *aureus* 55/2053 | NC_022113.1 | 2807 | 2.8 | complete |
| *Staphylococcus aureus* subsp. *aureus* 6850 | NC_022222.1 | 2739 | 2.7 | complete |
| *Staphylococcus aureus* subsp. *aureus* CN1 | NC_022226.1 | 2716 | 2.7 | complete |
| *Staphylococcus aureus* subsp. *aureus* SA957 | NC_022442.1 | 2794 | 2.8 | complete |
| *Staphylococcus aureus* subsp. *aureus* SA40 | NC_022443.1 | 2710 | 2.7 | complete |

**8.11 List of the 47 genomes used to make the *Mycobacterium tuberculosis* pangenome (partially adapted from (7) and (8))**

| **Organism/name** | **NCBI Accession number** | **Nb of genes** | **Size (Mb)** | **Level** |
| --- | --- | --- | --- | --- |
| *Mycobacterium tuberculosis* strain F1 | CP010329 | 4400 | 4.43 | complete |
| *Mycobacterium tuberculosis* strain F28 | CP010330 | 4366 | 4.42 | complete |
| *Mycobacterium tuberculosis* strain H37Ra | NC_009525 | 4348 | 4.42 | complete |
| *Mycobacterium tuberculosis* strain Erdman | NC_020559 | 4373 | 4.39 | complete |
| *Mycobacterium tuberculosis* strain 22103 | CP010339 | 4345 | 4.4 | complete |
| *Mycobacterium tuberculosis* strain 22115 | CP010337 | 4356 | 4.4 | complete |
| *Mycobacterium tuberculosis* strain 37004 | CP010338 | 4375 | 4.42 | complete |
| *Mycobacterium tuberculosis* strain KZN 4207 | NC_016768 | 4324 | 4.4 | complete |
| *Mycobacterium tuberculosis* strain KZN 605 | NC_018078 | 4326 | 4.4 | complete |
| *Mycobacterium tuberculosis* strain KZN 1435 | NC_012943 | 4333 | 4.4 | complete |
| *Mycobacterium tuberculosis* strain Haarlem | NC_022350 | 4322 | 4.41 | complete |
| *Mycobacterium tuberculosis* strain F11 | NC_009565 | 4352 | 4.42 | complete |
| *Mycobacterium tuberculosis* strain H37Rv | NC_000962 | 4336 | 4.41 | complete |
| *Mycobacterium tuberculosis* strain CDC1551 | NC_002755 | 4351 | 4.4 | complete |
| *Mycobacterium tuberculosis* strain 7199-99 | NC_020089 | 4344 | 4.42 | complete |
| *Mycobacterium tuberculosis* strain CTRI-2 | NC_017524 | 4331 | 4.4 | complete |
| *Mycobacterium tuberculosis* strain Kurono | NZ_AP014573 | 4342 | 4.42 | complete |
| *Mycobacterium tuberculosis* strain 26105 | CP010340 | 4393 | 4.43 | complete |
| *Mycobacterium tuberculosis* strain 2242 | CP010335 | 4428 | 4.42 | complete |
| *Mycobacterium tuberculosis* strain 2279 | CP010336 | 4400 | 4.41 | complete |
| *Mycobacterium tuberculosis* strain NITR203 | NC_021054 | 4442 | 4.41 | complete |
| *Mycobacterium tuberculosis* strain HKBS1 | CP002871 | 4343 | 4.41 | complete |
| *Mycobacterium tuberculosis* strain CCDC5079 | NC_021251 | 4354 | 4.41 | complete |
| *Mycobacterium tuberculosis* strain 49-02 | HG813240 | 4350 | 4.42 | complete |
| *Mycobacterium tuberculosis* strain 96075 | CP009426 | 4327 | 4.4 | complete |
| *Mycobacterium tuberculosis* strain BT1 | CP002883 | 4337 | 4.4 | complete |
| *Mycobacterium tuberculosis* strain BT2 | CP002882 | 4343 | 4.4 | complete |
| *Mycobacterium tuberculosis* strain CCDC5180 | CP002885 | 4353 | 4.41 | complete |
| *Mycobacterium tuberculosis* strain 323 | CP010873 | 4403 | 4.41 | complete |
| *Mycobacterium tuberculosis* strain ZMC13-88 | CP009101 | 4364 | 4.41 | complete |
| *Mycobacterium tuberculosis* strain ZMC13-264 | CP009100 | 4365 | 4.41 | complete |
| *Mycobacterium tuberculosis* strain KIT87190 | CP007809 | 4341 | 4.41 | complete |
| *Mycobacterium tuberculosis* strain K | CP007803 | 4334 | 4.39 | complete |
| *Mycobacterium tuberculosis* strain 96121 | CP009427 | 4375 | 4.41 | complete |
| *Mycobacterium tuberculosis* strain EAI5 | NC_021740 | 4322 | 4.39 | complete |
| *Mycobacterium tuberculosis* strain NITR206 | NC_021194 | 4417 | 4.39 | complete |
| *Mycobacterium tuberculosis* strain PanR0201 | CM002049.1 | 4352 | 4.41 | wgs |
| *Mycobacterium tuberculosis* strain PanR0802 | CM002050.1 | 4339 | 4.4 | wgs |
| *Mycobacterium tuberculosis* strain PanR1005 | CM002051.1 | 4322 | 4.39 | wgs |
| *Mycobacterium tuberculosis* strain PanR0704 | CM002048.1 | 4332 | 4.39 | wgs |
| *Mycobacterium tuberculosis* strain Beijing/NITR203 | CP005082.1 | 4461 | 4.41 | complete |
| *Mycobacterium tuberculosis* strain EAI5/NITR206 | CP005387.1 | 4433 | 4.39 | complete |
| *Mycobacterium tuberculosis* strain EAI5 | CP006578.1 | 4341 | 4.41 | complete |
| *Mycobacterium tuberculosis* strain UT205 | HE608151.1 | 4212 | 4.29 | complete |
| *Mycobacterium tuberculosis* strain 7199-99 | HE663067.1 | 3994 | 4.0 | complete |
| *Mycobacterium tuberculosis* strain H37Rv | AL123456.3 | 4367 | 4.41 | complete |
| *Mycobacterium tuberculosis* strain TCDC11 | CP046728.2 | 4121 | 4.2 | complete |

**8.12 List of the 226 genomes and plasmids used to make the *Salmonella typhimurium* pangenome**

| **Organism/name** | **NCBI Accession number** | Nb of genes | **Size (Mb)** | **Level** |
| --- | --- | --- | --- | --- |
| *Salmonella enterica* subsp. *enterica* serovar *Typhimurium* strain LT2 | AE006468.2 | 4714 | 5*.*0 | complete |
| *Salmonella enterica* subsp. *enterica* serovar *Typhimurium* strain LT2 plasmid pSLT | AE006471.2 | 102 | 0.1 | plasmid |
| *Salmonella enterica* subsp. *enterica* serovar *Typhimurium* strain ATCC 13311 | CP009102.1 | 4658 | 4*.*8 | complete |
| *Salmonella enterica* subsp. *enterica* serovar *Typhimurium* strain ATCC 13311 plasmid pSTY1 | CP009103.1 | 44 | 0 | plasmid |
| *Salmonella enterica* subsp. *enterica* serovar *Typhimurium* strain L-3553 | AP014565.1 | 5101 | 5*.*2 | complete |
| *Salmonella enterica* subsp. *enterica* serovar *Typhimurium* strain L-3553 plasmid pST3553 | AP014566.1 | 159 | 0.1 | plasmid |
| *Salmonella enterica* subsp. *enterica* serovar *Typhimurium* strain SO4698-09 | LN999997.1 | 4939 | 5*.*0 | complete |
| *Salmonella enterica* subsp. *enterica* serovar *Typhimurium* strain AUSMDU00008979 | CP045952.1 | 5188 | 5*.*2 | complete |
| *Salmonella enterica* subsp. *enterica* serovar *Typhimurium* strain AUSMDU00008979 plasmid pAUSMDU00008979_01 | CP045953.1 | 187 | 0.2 | plasmid |
| *Salmonella enterica* subsp. *enterica* serovar *Typhimurium* strain TW-Stm6 | CP019649.1 | 5176 | 5*.*3 | complete |
| *Salmonella enterica* subsp. *enterica* serovar *Typhimurium* strain TW-Stm6 plasmid p275_TW-Stm6 | CP019647.1 | 287 | 0.3 | plasmid |
| *Salmonella enterica* subsp. *enterica* serovar *Typhimurium* strain TW-Stm6 plasmid p4_TW-Stm6 | CP019648.1 | 4 | 0 | plasmid |
| *Salmonella enterica* subsp. *enterica* serovar *Typhimurium* strain WW012 | CP022168.1 | 5020 | 5*.*1 | complete |
| *Salmonella enterica* subsp. *enterica* serovar *Typhimurium* strain WW012 plasmid pWW012 | CP022169.1 | 0 | 0 | plasmid |
| *Salmonella enterica* subsp. *enterica* serovar *Typhimurium* strain VNB151-sc-2315230 | LT795114.1 | 5118 | 5*.*2 | complete |
| *Salmonella enterica* subsp. *enterica* serovar *Typhimurium* strain VNB151-sc-2315230 plasmid p1 | LT795115.1 | 0 | 0 | plasmid |
| *Salmonella enterica* subsp. *enterica* serovar *Typhimurium* strain VNB151-sc-2315230 plasmid p2 | LT795116.1 | 0 | 0 | plasmid |
| *Salmonella enterica* subsp. *enterica* serovar *Typhimurium* strain TJWQ005 | CP040458.1 | 5138 | 5*.*2 | complete |
| *Salmonella enterica* subsp. *enterica* serovar *Typhimurium* strain TJWQ005 plasmid unnamed1 | CP040457.1 | 0 | 0 | plasmid |
| *Salmonella enterica* subsp. *enterica* serovar *Typhimurium* strain 81741 | CP019442.1 | 5293 | 5*.*3 | complete |
| *Salmonella enterica* subsp. *enterica* serovar *Typhimurium* strain 81741 plasmid unnamed1 | CP019443.1 | 266 | 0.2 | plasmid |
| *Salmonella enterica* subsp. *enterica* serovar *Typhimurium* strain 81741 plasmid unnamed2 | CP019444.1 | 114 | 0.1 | plasmid |
| *Salmonella enterica* subsp. *enterica* serovar *Typhimurium* strain FDAARGOS_317 | CP027410.1 | 5204 | 5*.*2 | complete |
| *Salmonella enterica* subsp. *enterica* serovar *Typhimurium* strain FDAARGOS_317 plasmid unnamed | CP027409.1 | 273 | 0.2 | plasmid |
| *Salmonella enterica* subsp. *enterica* serovar *Typhimurium* strain SAP17-8290 | CP040568.1 | 5037 | 5*.*1 | complete |
| *Salmonella enterica* subsp. *enterica* serovar *Typhimurium* strain SAP17-8290 plasmid pCFSAN059542 | CP040569.1 | 121 | 0.1 | plasmid |
| *Salmonella enterica* subsp. *enterica* serovar *Typhimurium* strain AUSMDU00010530 | CP045947.1 | 4849 | 5*.*0 | complete |
| *Salmonella enterica* subsp. *enterica* serovar *Typhimurium* strain AUSMDU00010530 plasmid pAUSMDU00010530_01 | CP045948.1 | 6 | 0 | plasmid |
| *Salmonella enterica* subsp. *enterica* serovar *Typhimurium* strain NCCP 16207 | CP041976.1 | 5004 | 5*.*1 | complete |
| *Salmonella enterica* subsp. *enterica* serovar *Typhimurium* strain NCCP 16207 plasmid unnamed1 | CP041974.1 | 151 | 0.1 | plasmid |
| *Salmonella enterica* subsp. *enterica* serovar *Typhimurium* strain NCCP 16207 plasmid unnamed2 | CP041975.1 | 23 | 0 | plasmid |
| *Salmonella enterica* subsp. *enterica* serovar *Typhimurium* strain T000240 | AP011957.1 | 4956 | 5*.*1 | complete |
| *Salmonella enterica* subsp. *enterica* serovar *Typhimurium* strain T000240 plasmid pSTMDT12_L | AP011958.1 | 139 | 0.1 | plasmid |
| *Salmonella enterica* subsp. *enterica* serovar *Typhimurium* strain T000240 plasmid pSTMDT12_S | AP011959.1 | 10 | 0 | plasmid |
| *Salmonella enterica* subsp. *enterica* serovar *Typhimurium* strain PNCS014862 | CP039591.1 | 4909 | 5*.*0 | complete |
| *Salmonella enterica* subsp. *enterica* serovar *Typhimurium* strain PNCS014862 plasmid p11-0500.1 | CP039592.1 | 121 | 0.1 | plasmid |
| *Salmonella enterica* subsp. *enterica* serovar *Typhimurium* strain USDA-ARS-USMARC-1808 | CP014969.1 | 4919 | 5*.*0 | complete |
| *Salmonella enterica* subsp. *enterica* serovar *Typhimurium* strain USDA-ARS-USMARC-1808 plasmid pSTY1-1808 | CP014970.1 | 112 | 0.1 | plasmid |
| *Salmonella enterica* subsp. *enterica* serovar *Typhimurium* strain CDC 2009K-1640 | CP014975.1 | 4998 | 5*.*0 | complete |
| *Salmonella enterica* subsp. *enterica* serovar *Typhimurium* strain CDC 2009K-1640 plasmid pSTY1-2009K-1640 | CP014976.1 | 111 | 0.1 | plasmid |
| *Salmonella enterica* subsp. *enterica* serovar *Typhimurium* strain DT104 | HF937208.1 | 4918 | 5*.*0 | complete |
| *Salmonella enterica* subsp. *enterica* serovar *Typhimurium* strain DT104 plasmid II | HF937209.1 | 106 | 0.1 | plasmid |
| *Salmonella enterica* subsp. *enterica* serovar *Typhimurium* strain 138736 | CP007581.1 | 4997 | 5*.*0 | complete |
| *Salmonella enterica* subsp. *enterica* serovar *Typhimurium* strain 138736 plasmid unnamed | CP007582.1 | 105 | 0.1 | plasmid |
| *Salmonella enterica* subsp. *enterica* serovar *Typhimurium* strain YU15 | CP014358.1 | 4914 | 5*.*0 | complete |
| *Salmonella enterica* subsp. *enterica* serovar *Typhimurium* strain YU15 plasmid pYU15_94 | CP014359.1 | 114 | 0.1 | plasmid |
| *Salmonella enterica* subsp. *enterica* serovar *Typhimurium* strain PNCS014856 | CP039576.1 | 4974 | 5*.*1 | complete |
| *Salmonella enterica* subsp. *enterica* serovar *Typhimurium* strain PNCS014856 plasmid p10-3857.1 | CP039577.1 | 191 | 0.2 | plasmid |
| *Salmonella enterica* subsp. *enterica* serovar *Typhimurium* strain PNCS014856 plasmid p10-3857.2 | CP039578.1 | 6 | 0 | plasmid |
| *Salmonella enterica* subsp. *enterica* serovar *Typhimurium* strain sg_wt7 | CP036168.1 | 4943 | 4*.*9 | complete |
| *Salmonella enterica* subsp. *enterica* serovar *Typhimurium* str. USDA-ARS-USMARC-1810 strain USDA-ARS-USMARC-1810 | CP014982.2 | 4855 | 4*.*9 | complete |
| *Salmonella enterica* subsp. *enterica* serovar *Typhimurium* strain PNCS014879 | CP040321.1 | 4907 | 5*.*0 | complete |
| *Salmonella enterica* subsp. *enterica* serovar *Typhimurium* strain PNCS014879 plasmid p16-6397.1 | CP040322.1 | 121 | 0.1 | plasmid |
| *Salmonella enterica* subsp. *enterica* serovar *Typhimurium* strain PNCS014879 plasmid p16-6397.2 | CP040323.1 | 9 | 0 | plasmid |
| *Salmonella enterica* subsp. *enterica* serovar *Typhimurium* strain SO2 | CP014356.1 | 4888 | 5*.*0 | complete |
| *Salmonella enterica* subsp. *enterica* serovar *Typhimurium* strain SO2 plasmid pSO2_STV | CP014357.1 | 115 | 0.1 | plasmid |
| *Salmonella enterica* subsp. *enterica* serovar *Typhimurium* strain B3589 | CP034968.1 | 4962 | 4*.*9 | complete |
| *Salmonella enterica* subsp. *enterica* serovar *Typhimurium* strain B3589 plasmid p3589 | CP034969.1 | 7 | 0 | plasmid |
| *Salmonella enterica* subsp. *enterica* serovar *Typhimurium* strain SO3 | CP014536.1 | 4882 | 5*.*0 | complete |
| *Salmonella enterica* subsp. *enterica* serovar *Typhimurium* strain SO3 plasmid pSO3_STV | CP014537.1 | 113 | 0.1 | plasmid |
| *Salmonella enterica* subsp. *enterica* serovar *Typhimurium* strain CDC 2011K-1702 | CP014967.1 | 4944 | 5*.*0 | complete |
| *Salmonella enterica* subsp. *enterica* serovar *Typhimurium* strain CDC 2011K-1702 plasmid pSTY1-2011K-1702 | CP014968.1 | 112 | 0.1 | plasmid |
| *Salmonella enterica* subsp. *enterica* serovar *Typhimurium* strain SAP17-7399 | CP040562.1 | 4835 | 4*.*9 | complete |
| *Salmonella enterica* subsp. *enterica* serovar *Typhimurium* strain SAP17-7399 plasmid pCFSAN059545 | CP040563.1 | 5 | 0 | plasmid |
| *Salmonella enterica* subsp. *enterica* serovar *Typhimurium* strain YU39 | CP011428.1 | 5111 | 5*.*2 | complete |
| *Salmonella enterica* subsp. *enterica* serovar *Typhimurium* strain YU39 plasmid pYU39_2.7 | CP011435.1 | 4 | 0 | plasmid |
| *Salmonella enterica* subsp. *enterica* serovar *Typhimurium* strain YU39 plasmid pYU39_4.2 | CP011434.1 | 4 | 0 | plasmid |
| *Salmonella enterica* subsp. *enterica* serovar *Typhimurium* strain YU39 plasmid pYU39_4.8 | CP011433.1 | 5 | 0 | plasmid |
| *Salmonella enterica* subsp. *enterica* serovar *Typhimurium* strain YU39 plasmid pYU39_5.1 | CP011432.1 | 3 | 0 | plasmid |
| *Salmonella enterica* subsp. *enterica* serovar *Typhimurium* strain YU39 plasmid pYU39_89 | CP011430.1 | 109 | 0.1 | plasmid |
| *Salmonella enterica* subsp. *enterica* serovar *Typhimurium* strain YU39 plasmid pYU39_IncA/C | CP011429.1 | 178 | 0.1 | plasmid |
| *Salmonella enterica* subsp. *enterica* serovar *Typhimurium* strain YU39 plasmid pYU39_IncX | CP011431.1 | 48 | 0 | plasmid |
| *Salmonella enterica* subsp. *enterica* serovar *Typhimurium* strain FDAARGOS_321 | CP022070.2 | 4986 | 5*.*0 | complete |
| *Salmonella enterica* subsp. *enterica* serovar *Typhimurium* strain FDAARGOS_321 plasmid unnamed1 | CP022071.2 | 120 | 0.1 | plasmid |
| *Salmonella enterica* subsp. *enterica* serovar *Typhimurium* strain FDAARGOS_321 plasmid unnamed2 | CP022072.2 | 65 | 0 | plasmid |
| *Salmonella enterica* subsp. *enterica* serovar *Typhimurium* strain CDC H2662 | CP014979.2 | 4938 | 5*.*0 | complete |
| *Salmonella enterica* subsp. *enterica* serovar *Typhimurium* strain CDC H2662 plasmid pSTY1-H2662 | CP014980.1 | 121 | 0.1 | plasmid |
| *Salmonella enterica* subsp. *enterica* serovar *Typhimurium* var. 5- strain CFSAN067216 | CP028318.1 | 4933 | 5*.*0 | complete |
| *Salmonella enterica* subsp. *enterica* serovar *Typhimurium* var. 5- strain CFSAN067216 plasmid pSC-09-1 | CP028319.1 | 120 | 0.1 | plasmid |
| *Salmonella enterica* subsp. *enterica* serovar *Typhimurium* strain SAP17-7699 | CP040564.1 | 4913 | 5*.*0 | complete |
| *Salmonella enterica* subsp. *enterica* serovar *Typhimurium* strain SAP17-7699 plasmid pCFSAN059544 | CP040565.1 | 120 | 0.1 | plasmid |
| *Salmonella enterica* subsp. *enterica* serovar *Typhimurium* strain D23580 | LS997973.1 | 5037 | 5*.*1 | complete |
| *Salmonella enterica* subsp. *enterica* serovar *Typhimurium* strain D23580 plasmid D23580_liv_pBT1 | LS997975.1 | - | **-** | plasmid |
| *Salmonella enterica* subsp. *enterica* serovar *Typhimurium* strain D23580 plasmid D23580_liv_pBT2 | LS997976.1 | - | **-** | plasmid |
| *Salmonella enterica* subsp. *enterica* serovar *Typhimurium* strain D23580 plasmid D23580_liv_pBT3 | LS997977.1 | - | **-** | plasmid |
| *Salmonella enterica* subsp. *enterica* serovar *Typhimurium* strain D23580 plasmid D23580_liv_pSLT-BT | LS997974.1 | - | **-** | plasmid |
| *Salmonella enterica* subsp. *enterica* serovar *Typhimurium* str. D23580 strain D23580 | FN424405.1 | 4729 | 4*.*9 | complete |
| *Salmonella enterica* subsp. *enterica* serovar *Typhimurium* strain UGA14 | CP021462.1 | 5249 | 5*.*4 | complete |
| *Salmonella enterica* subsp. *enterica* serovar *Typhimurium* strain UGA14 plasmid pUGA14_1 | CP021463.1 | 389 | 0.4 | plasmid |
| *Salmonella enterica* subsp. *enterica* serovar *Typhimurium* strain UGA14 plasmid pUGA14_2 | CP021464.1 | 154 | 0.1 | plasmid |
| *Salmonella enterica* subsp. *enterica* serovar *Typhimurium* strain UGA14 plasmid pUGA14_3 | CP021465.1 | 4 | 0 | plasmid |
| *Salmonella enterica* subsp. *enterica* serovar *Typhimurium* strain UGA14 plasmid pUGA14_4 | CP021466.1 | 2 | 0 | plasmid |
| *Salmonella enterica* subsp. *enterica* serovar *Typhimurium* strain ST4/74 | CP002487.1 | 4941 | 5*.*1 | complete |
| *Salmonella enterica* subsp. *enterica* serovar *Typhimurium* strain ST4/74 plasmid TY474p1 | CP002488.1 | 116 | 0.1 | plasmid |
| *Salmonella enterica* subsp. *enterica* serovar *Typhimurium* strain ST4/74 plasmid TY474p2 | CP002489.1 | 90 | 0.1 | plasmid |
| *Salmonella enterica* subsp. *enterica* serovar *Typhimurium* strain ST4/74 plasmid TY474p3 | CP002490.1 | 11 | 0 | plasmid |
| *Salmonella enterica* subsp. *enterica* serovar *Typhimurium* strain SL1344 | FQ312003.1 | 4941 | 5*.*1 | complete |
| *Salmonella enterica* subsp. *enterica* serovar *Typhimurium* strain SL1344 plasmid pCol1B9_SL1344 | HE654725.1 | 101 | 0.1 | plasmid |
| *Salmonella enterica* subsp. *enterica* serovar *Typhimurium* strain SL1344 plasmid pRSF1010_SL1344 | HE654726.1 | 12 | 0 | plasmid |
| *Salmonella enterica* subsp. *enterica* serovar *Typhimurium* strain SL1344 plasmid pSLT_SL1344 | HE654724.1 | 101 | 0.1 | plasmid |
| *Salmonella enterica* subsp. *enterica* serovar *Typhimurium* strain 798 | CP003386.1 | 4897 | 5*.*0 | complete |
| *Salmonella enterica* subsp. *enterica* serovar *Typhimurium* strain 798 plasmid p798_93 | CP003387.1 | 104 | 0.1 | plasmid |
| *Salmonella enterica* subsp. *enterica* serovar *Typhimurium* strain 14028S substr. GXS275 | CP043399.1 | 4772 | 4*.*9 | complete |
| *Salmonella enterica* subsp. *enterica* serovar *Typhimurium* strain RM13672 | CP047323.1 | 4825 | 5*.*0 | complete |
| *Salmonella enterica* subsp. *enterica* serovar *Typhimurium* strain RM13672 plasmid pRM13672 | CP047324.1 | 111 | 0.1 | plasmid |
| *Salmonella enterica* subsp. *enterica* serovar *Typhimurium* strain 14028S | CP001363.1 | 4818 | 5*.*0 | complete |
| *Salmonella enterica* subsp. *enterica* serovar *Typhimurium* strain 14028S plasmid unnamed | CP001362.1 | 102 | 0.1 | plasmid |
| *Salmonella enterica* subsp. *enterica* serovar *Typhimurium* str. 14028S strain 14028S substr. JY996 | CP043400.1 | 4769 | 4*.*9 | complete |
| *Salmonella enterica* subsp. *enterica* serovar *Typhimurium* strain AR_0031 | CP026700.1 | 4825 | 5*.*0 | complete |
| *Salmonella enterica* subsp. *enterica* serovar *Typhimurium* strain AR_0031 plasmid unitig_1_pilon | CP026701.1 | 120 | 0.1 | plasmid |
| *Salmonella enterica* subsp. *enterica* serovar *Typhimurium* strain PIR00538 | CP025555.1 | 4826 | 5*.*0 | complete |
| *Salmonella enterica* subsp. *enterica* serovar *Typhimurium* strain PIR00538 plasmid pPIR00538 | CP025556.1 | 120 | 0.1 | plasmid |
| *Salmonella enterica* subsp. *enterica* serovar *Typhimurium* strain 14028 | CP034479.1 | 4889 | 5*.*0 | complete |
| *Salmonella enterica* subsp. *enterica* serovar *Typhimurium* strain 14028 plasmid unnamed | CP034480.1 | 119 | 0.1 | plasmid |
| *Salmonella enterica* subsp. *enterica* serovar *Typhimurium* strain 01ST04081 | CP029840.1 | 5093 | 5*.*1 | complete |
| *Salmonella enterica* subsp. *enterica* serovar *Typhimurium* strain 01ST04081 plasmid p01ST04081A | CP029841.1 | 189 | 0.2 | plasmid |
| *Salmonella enterica* subsp. *enterica* serovar *Typhimurium* strain 01ST04081 plasmid p01ST04081B | CP029842.1 | 139 | 0.1 | plasmid |
| *Salmonella enterica* subsp. *enterica* serovar *Typhimurium* strain ATCC 14028 | CP034230.1 | 5006 | 5*.*0 | complete |
| *Salmonella enterica* subsp. *enterica* serovar *Typhimurium* strain ATCC 14028 plasmid pATCC14028 | CP034231.1 | 124 | 0.1 | plasmid |
| *Salmonella enterica* subsp. *enterica* serovar *Typhimurium* strain 14028S substr. GXS259 | CP043401.1 | 4771 | 4*.*9 | complete |
| *Salmonella enterica* subsp. *enterica* serovar *Typhimurium* strain 14028S substr. GXS254 | CP043402.1 | 4774 | 4*.*9 | complete |
| *Salmonella enterica* subsp. *enterica* serovar *Typhimurium* strain AUSMDU00010529 | CP045949.1 | 4922 | 5*.*1 | complete |
| *Salmonella enterica* subsp. *enterica* serovar *Typhimurium* strain AUSMDU00010529 plasmid pAUSMDU00010529_01 | CP045950.1 | 112 | 0.1 | plasmid |
| *Salmonella enterica* subsp. *enterica* serovar *Typhimurium* strain AUSMDU00010529 plasmid pAUSMDU00010529_02 | CP045951.1 | 99 | 0.1 | plasmid |
| *Salmonella enterica* subsp. *enterica* serovar *Typhimurium* strain 10ST07093 | CP029839.1 | 5002 | 5*.*1 | complete |
| *Salmonella enterica* subsp. *enterica* serovar *Typhimurium* strain 10ST07093 plasmid p10ST07093A | CP029837.1 | 120 | 0.1 | plasmid |
| *Salmonella enterica* subsp. *enterica* serovar *Typhimurium* strain 10ST07093 plasmid p10ST07093B | CP029838.1 | 120 | 0.1 | plasmid |
| *Salmonella enterica* subsp. *enterica* serovar *Typhimurium* var. 5- strain CFSAN001921 | CP006048.1 | 4967 | 5*.*1 | complete |
| *Salmonella enterica* subsp. *enterica* serovar *Typhimurium* var. 5- strain CFSAN001921 plasmid unnamed | CP006050.1 | 233 | 0.2 | plasmid |
| *Salmonella enterica* subsp. *enterica* serovar *Typhimurium* var. 5- strain CFSAN001921 plasmid unnamed2 | CP006051.1 | 5 | 0 | plasmid |
| *Salmonella enterica* subsp. *enterica* serovar *Typhimurium* var. 5- strain CFSAN001921 plasmid unnamed3 | CP006052.1 | 2 | 0 | plasmid |
| *Salmonella enterica* subsp. *enterica* serovar *Typhimurium* strain SAP17-7299 | CP040566.1 | 4908 | 5*.*0 | complete |
| *Salmonella enterica* subsp. *enterica* serovar *Typhimurium* strain SAP17-7299 plasmid pCFSAN059543 | CP040567.1 | 133 | 0.1 | plasmid |
| *Salmonella enterica* subsp. *enterica* serovar *Typhimurium* strain CFSAN018746 | CP028199.1 | 4807 | 5*.*0 | complete |
| *Salmonella enterica* subsp. *enterica* serovar *Typhimurium* strain CFSAN018746 plasmid pGMI14-001 | CP028200.1 | 120 | 0.1 | plasmid |
| *Salmonella enterica* subsp. *enterica* serovar *Typhimurium* strain RM10961 | CP013702.1 | 4772 | 4*.*9 | complete |
| *Salmonella enterica* subsp. *enterica* serovar *Typhimurium* strain USDA-ARS-USMARC-1896 | CP014977.1 | 4897 | 5*.*0 | complete |
| *Salmonella enterica* subsp. *enterica* serovar *Typhimurium* strain USDA-ARS-USMARC-1896 plasmid pSTY1-1896 | CP014978.1 | 176 | 0.1 | plasmid |
| *Salmonella enterica* subsp. *enterica* serovar *Typhimurium* strain USDA-ARS-USMARC-1899 | CP007235.2 | 4698 | 4*.*9 | complete |
| *Salmonella enterica* subsp. *enterica* serovar *Typhimurium* strain FORC098 | CP030029.1 | 4747 | 4*.*9 | complete |
| *Salmonella enterica* subsp. *enterica* serovar *Typhimurium* strain PNCS014851 | CP038847.1 | 4799 | 4*.*9 | complete |
| *Salmonella enterica* subsp. *enterica* serovar *Typhimurium* strain PNCS014851 plasmid p09-0499.1 | CP038848.1 | 119 | 0.1 | plasmid |
| *Salmonella enterica* subsp. *enterica* serovar *Typhimurium* strain ST931R | CP016385.1 | 5184 | 5*.*2 | complete |
| *Salmonella enterica* subsp. *enterica* serovar *Typhimurium* strain ST931R plasmid p931-3904 | CP016386.1 | 3 | 0 | plasmid |
| *Salmonella enterica* subsp. *enterica* serovar *Typhimurium* strain ST931R plasmid p931IncI1 | CP016387.1 | 102 | 0.1 | plasmid |
| *Salmonella enterica* subsp. *enterica* serovar *Typhimurium* strain ST931R plasmid p931IncI2 | CP016388.1 | 69 | 0 | plasmid |
| *Salmonella enterica* subsp. *enterica* serovar *Typhimurium* strain ST931R plasmid pESBL931 | CP016389.1 | 78 | 0.1 | plasmid |
| *Salmonella enterica* subsp. *enterica* serovar *Typhimurium* strain ST931R plasmid pSLT931 | CP016390.1 | 111 | 0.1 | plasmid |
| *Salmonella enterica* subsp. *enterica* serovar *Typhimurium* strain U288 | CP003836.1 | 4893 | 5*.*0 | complete |
| *Salmonella enterica* subsp. *enterica* serovar *Typhimurium* strain U288 plasmid pSTU288-1 | CP004058.1 | 200 | 0.2 | plasmid |
| *Salmonella enterica* subsp. *enterica* serovar *Typhimurium* strain U288 plasmid pSTU288-2 | CP004059.1 | 12 | 0 | plasmid |
| *Salmonella enterica* subsp. *enterica* serovar *Typhimurium* strain U288 plasmid pSTU288-3 | CP004060.1 | 5 | 0 | plasmid |
| *Salmonella enterica* subsp. *enterica* serovar *Typhimurium* strain NC983 | CP015157.1 | 4869 | 4*.*9 | complete |
| *Salmonella enterica* subsp. *enterica* serovar *Typhimurium* strain NC983 plasmid unnamed | CP015158.1 | 111 | 0.1 | plasmid |
| *Salmonella enterica* subsp. *enterica* serovar *Typhimurium* strain SL26 | CP032490.1 | 5005 | 5*.*1 | complete |
| *Salmonella enterica* subsp. *enterica* serovar *Typhimurium* strain SL26 plasmid pSL26_91 | CP032492.1 | 116 | 0.1 | plasmid |
| *Salmonella enterica* subsp. *enterica* serovar *Typhimurium* strain SL26 plasmid pSL26_ColRNAI | CP032493.1 | 6 | 0 | plasmid |
| *Salmonella enterica* subsp. *enterica* serovar *Typhimurium* strain SL26 plasmid pSL26_IncA/C2 | CP032491.1 | 203 | 0.2 | plasmid |
| *Salmonella enterica* subsp. *enterica* serovar *Typhimurium* strain BL10 | CP024619.1 | 5051 | 5*.*2 | complete |
| *Salmonella enterica* subsp. *enterica* serovar *Typhimurium* strain BL10 plasmid p10k | CP025337.1 | 12 | 0 | plasmid |
| *Salmonella enterica* subsp. *enterica* serovar *Typhimurium* strain BL10 plasmid p113k | CP025339.1 | 143 | 0.1 | plasmid |
| *Salmonella enterica* subsp. *enterica* serovar *Typhimurium* strain BL10 plasmid p11k | CP025338.2 | 17 | 0 | plasmid |
| *Salmonella enterica* subsp. *enterica* serovar *Typhimurium* strain BL10 plasmid p220k | CP025340.1 | 236 | 0.2 | plasmid |
| *Salmonella enterica* subsp. *enterica* serovar *Typhimurium* strain BL10 plasmid p3.8k | CP025336.1 | 6 | 0 | plasmid |
| *Salmonella enterica* subsp. *enterica* serovar *Typhimurium* strain CDC 2009K-2059 | CP014983.1 | 4719 | 4*.*8 | complete |
| *Salmonella enterica* subsp. *enterica* serovar *Typhimurium* strain FDAARGOS_320 | CP027414.1 | 4982 | 5*.*0 | complete |
| *Salmonella enterica* subsp. *enterica* serovar *Typhimurium* strain FDAARGOS_320 plasmid unnamed1 | CP027415.1 | 144 | 0.1 | plasmid |
| *Salmonella enterica* subsp. *enterica* serovar *Typhimurium* strain FDAARGOS_320 plasmid unnamed2 | CP027413.1 | 120 | 0.1 | plasmid |
| *Salmonella enterica* subsp. *enterica* serovar *Typhimurium* strain FDAARGOS_320 plasmid unnamed3 | CP027416.1 | 10 | 0 | plasmid |
| *Salmonella enterica* subsp. *enterica* serovar *Typhimurium* strain SARA13 | CP017728.1 | 5019 | 4*.*9 | complete |
| *Salmonella enterica* subsp. *enterica* serovar *Typhimurium* strain SARA13 plasmid pSARA13 | CP017729.1 | 111 | 0.1 | plasmid |
| *Salmonella enterica* subsp. *enterica* serovar *Typhimurium* strain UK-1 | CP002614.1 | 4751 | 4*.*9 | complete |
| *Salmonella enterica* subsp. *enterica* serovar *Typhimurium* strain UK-1 plasmid pSTUK-100 | CP002615.1 | 100 | 0.1 | plasmid |
| *Salmonella enterica* subsp. *enterica* serovar *Typhimurium* strain 22495 | CP017617.1 | 4949 | 4*.*9 | complete |
| *Salmonella enterica* subsp. *enterica* serovar *Typhimurium* strain 22495 plasmid unnamed1 | CP017618.1 | 112 | 0.1 | plasmid |
| *Salmonella enterica* subsp. *enterica* serovar *Typhimurium* strain 22495 plasmid unnamed2 | CP017619.1 | 1 | 0 | plasmid |
| *Salmonella enterica* subsp. *enterica* serovar *Typhimurium* strain USDA-ARS-USMARC-1880 | CP014981.1 | 4643 | 4*.*8 | complete |
| *Salmonella enterica* subsp. *enterica* serovar *Typhimurium* strain DT2 | HG326213.1 | 4824 | 4*.*9 | complete |
| *Salmonella enterica* subsp. *enterica* serovar *Typhimurium* strain DT2 plasmid pSLT | LN999012.1 | 121 | 0.1 | plasmid |
| *Salmonella enterica* subsp. *enterica* serovar *Typhimurium* strain SAP18-6199 | CP040900.1 | 4848 | 4*.*9 | complete |
| *Salmonella enterica* subsp. *enterica* serovar *Typhimurium* strain SAP18-6199 plasmid pCFSAN074387 | CP040901.1 | 121 | 0.1 | plasmid |
| *Salmonella enterica* subsp. *enterica* serovar *Typhimurium* strain STMU2UK | LT855376.1 | 4753 | 4*.*9 | complete |
| *Salmonella enterica* subsp. *enterica* serovar *Typhimurium* strain STMU2UK plasmid 2 | LT855377.1 | 100 | 0.1 | plasmid |
| *Salmonella enterica* subsp. *enterica* serovar *Typhimurium* strain 33676 | CP012681.1 | 5063 | 5*.*1 | complete |
| *Salmonella enterica* subsp. *enterica* serovar *Typhimurium* strain 33676 plasmid p33673_IncF | CP012683.1 | 115 | 0.1 | plasmid |
| *Salmonella enterica* subsp. *enterica* serovar *Typhimurium* strain 33676 plasmid p33676_4.5 | CP012684.1 | 5 | 0 | plasmid |
| *Salmonella enterica* subsp. *enterica* serovar *Typhimurium* strain 33676 plasmid p33676_IncA/C | CP012682.1 | 198 | 0.2 | plasmid |
| *Salmonella enterica* subsp. *enterica* serovar *Typhimurium* var. 5- strain CFSAN067217 | CP028314.1 | 5043 | 5*.*1 | complete |
| *Salmonella enterica* subsp. *enterica* serovar *Typhimurium* var. 5- strain CFSAN067217 plasmid pSC-31-1 | CP028315.1 | 129 | 0.1 | plasmid |
| *Salmonella enterica* subsp. *enterica* serovar *Typhimurium* var. 5- strain CFSAN067217 plasmid pSC-31-2 | CP028316.1 | 217 | 0.2 | plasmid |
| *Salmonella enterica* subsp. *enterica* serovar *Typhimurium* var. 5- strain CFSAN067217 plasmid pSC-31-3 | CP028317.1 | 1 | 0 | plasmid |
| *Salmonella enterica* subsp. *enterica* serovar *Typhimurium* strain 22792 | CP017621.1 | 4817 | 4*.*9 | complete |
| *Salmonella enterica* subsp. *enterica* serovar *Typhimurium* strain 22792 plasmid unnamed1 | CP017620.1 | 111 | 0.1 | plasmid |
| *Salmonella enterica* subsp. *enterica* serovar *Typhimurium* strain FORC_020 | CP012144.1 | 4705 | 4*.*8 | complete |
| *Salmonella enterica* subsp. *enterica* serovar *Typhimurium* strain CDC 2010K-1587 | CP014965.1 | 4988 | 5*.*0 | complete |
| *Salmonella enterica* subsp. *enterica* serovar *Typhimurium* strain CDC 2010K-1587 plasmid pSTY1-2010K-1587 | CP016864.1 | 133 | 0.1 | plasmid |
| *Salmonella enterica* subsp. *enterica* serovar *Typhimurium* strain CDC 2010K-1587 plasmid pSTY2-2010K-1587 | CP016865.1 | 116 | 0.1 | plasmid |
| *Salmonella enterica* subsp. *enterica* serovar *Typhimurium* strain CDC 2010K-1587 plasmid pSTY3-2010K-1587 | CP016866.1 | 6 | 0 | plasmid |
| *Salmonella enterica* subsp. *enterica* serovar *Typhimurium* strain CDC 2010K-1587 plasmid pSTY4-2010K-1587 | CP016867.1 | 4 | 0 | plasmid |
| *Salmonella enterica* subsp. *enterica* serovar *Typhimurium* strain USDA-ARS-USMARC-1898 | CP014971.2 | 4859 | 5*.*0 | complete |
| *Salmonella enterica* subsp. *enterica* serovar *Typhimurium* strain USDA-ARS-USMARC-1898 plasmid pSTY1-1898 | CP014972.2 | 122 | 0.1 | plasmid |
| *Salmonella enterica* subsp. *enterica* serovar *Typhimurium* strain USDA-ARS-USMARC-1898 plasmid pSTY2-1898 | CP014973.1 | 121 | 0.1 | plasmid |
| *Salmonella enterica* subsp. *enterica* serovar *Typhimurium* strain USDA-ARS-USMARC-1898 plasmid pSTY3-1898 | CP014974.1 | 47 | 0 | plasmid |
| *Salmonella enterica* subsp. *enterica* serovar *Typhimurium* strain CDC 2011K-0870 | CP007523.1 | 4634 | 4*.*8 | complete |
| *Salmonella enterica* subsp. *enterica* serovar *Typhimurium* strain RSE04 | CP034719.1 | 4875 | 4*.*9 | complete |
| *Salmonella enterica* subsp. *enterica* serovar *Typhimurium* strain RSE04 plasmid pRSE04 | CP034720.1 | 119 | 0.1 | plasmid |
| *Salmonella enterica* subsp. *enterica* serovar *Typhimurium* strain SO21 | CP032494.1 | 4937 | 5*.*1 | complete |
| *Salmonella enterica* subsp. *enterica* serovar *Typhimurium* strain SO21 plasmid pSO21_118 | CP032495.1 | 149 | 0.1 | plasmid |
| *Salmonella enterica* subsp. *enterica* serovar *Typhimurium* strain SO21 plasmid pSO21_75 | CP032497.1 | 101 | 0.1 | plasmid |
| *Salmonella enterica* subsp. *enterica* serovar *Typhimurium* strain SO21 plasmid pSO21_ColRNAI_5.8 | CP032499.1 | 12 | 0 | plasmid |
| *Salmonella enterica* subsp. *enterica* serovar *Typhimurium* strain SO21 plasmid pSO21_ColRNAI_6.8 | CP032498.1 | 3 | 0 | plasmid |
| *Salmonella enterica* subsp. *enterica* serovar *Typhimurium* strain SO21 plasmid pSO21_IncA/C2 | CP032496.1 | 126 | 0.1 | plasmid |
| *Salmonella enterica* subsp. *enterica* serovar *Typhimurium* strain VNP20009 | CP007804.2 | 4791 | 4*.*9 | complete |
| *Salmonella enterica* subsp. *enterica* serovar *Typhimurium* strain VNP20009 plasmid pSLT_VNP20009 | CP008745.1 | 142 | 0.1 | plasmid |
| *Salmonella enterica* subsp. *enterica* serovar *Typhimurium* strain FORC_015 | CP011365.1 | 4667 | 4*.*8 | complete |
| *Salmonella enterica* subsp. *enterica* serovar *Typhimurium* strain FORC58 | CP020565.1 | 4525 | 4*.*7 | complete |
| *Salmonella enterica* subsp. *enterica* serovar *Typhimurium* strain FORC50 | CP019383.1 | 4519 | 4*.*7 | complete |
| *Salmonella enterica* subsp. *enterica* serovar *Typhimurium* strain FORC88 | CP029029.1 | 4517 | 4*.*6 | complete |
| *Salmonella enterica* subsp. *enterica* serovar *Typhimurium* strain RM9437 | CP012985.1 | 4792 | 5*.*1 | complete |
| *Salmonella enterica* subsp. *enterica* serovar *Typhimurium* strain RM9437 plasmid pRM9437 | CP014577.1 | 95 | 0.1 | plasmid |
| *Salmonella enterica* subsp. *enterica* serovar *Typhimurium* strain E40 | CP038432.1 | 5027 | 5*.*0 | complete |
| *Salmonella enterica* subsp. *enterica* serovar *Typhimurium* strain E40 plasmid unnamed | CP038433.1 | 121 | 0.1 | plasmid |
| *Salmonella enterica* subsp. *enterica* serovar *Typhimurium* strain E40V | CP038434.1 | 5027 | 5*.*0 | complete |
| *Salmonella enterica* subsp. *enterica* serovar *Typhimurium* strain E40V plasmid unnamed | CP038435.1 | 121 | 0.1 | plasmid |
| *Salmonella enterica* subsp. *enterica* serovar *Typhimurium* strain RM10607 | CP013720.1 | 5117 | 5*.*0 | complete |
| *Salmonella enterica* subsp. *enterica* serovar *Typhimurium* strain RM10607 plasmid pRM10607 | CP013721.1 | 98 | 0.1 | plasmid |

**9 Visualization of integrated plasmids and chromosomal portion in *B. subtilis* GM (10-11)**

Visualization of DUGMO results for plasmids and insertion of a chromosomal portion in *B. subtilis* GM. The genes correctly detected by DUGMO are in green (true positives). The genes discarded by DUGMO are in red (false negatives). Genes belonging to *B. subtilis* are in blue. Genes aligning on the pangenome of *B. subtilis* during BLASTN alignment are in orange.

**9.1 Visualization of integrated plasmids in *B. subtilis* GM (10-11)**

**
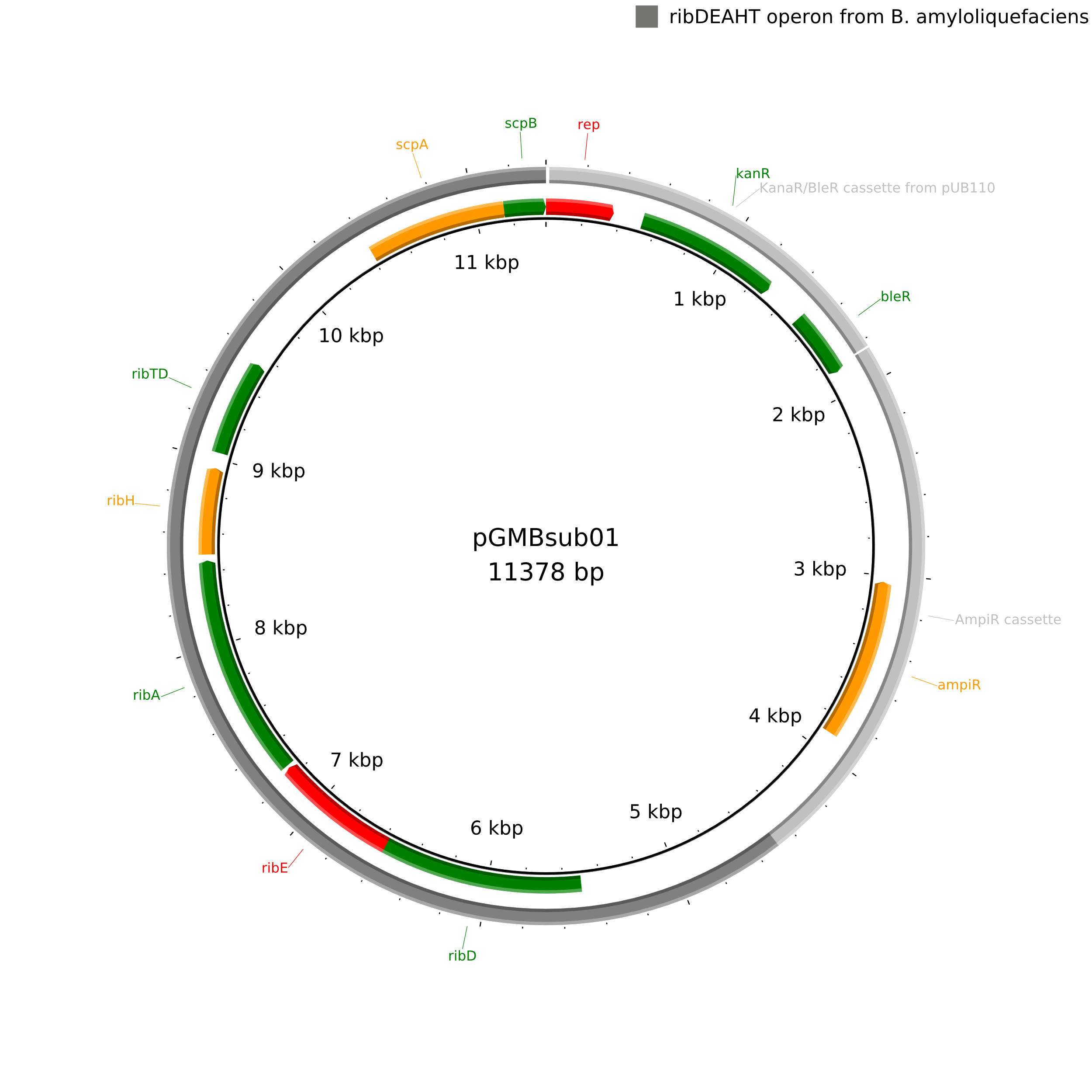

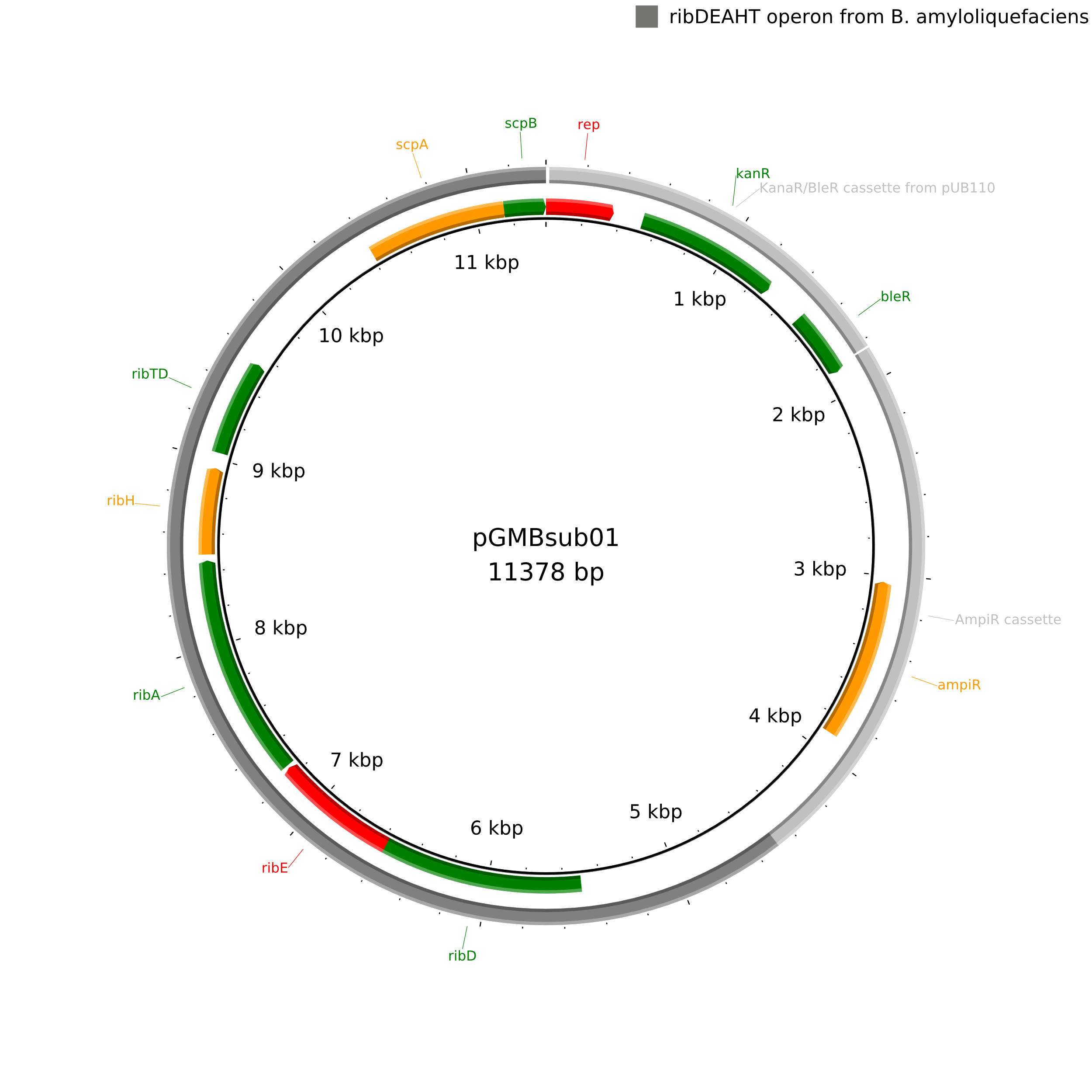
**

**
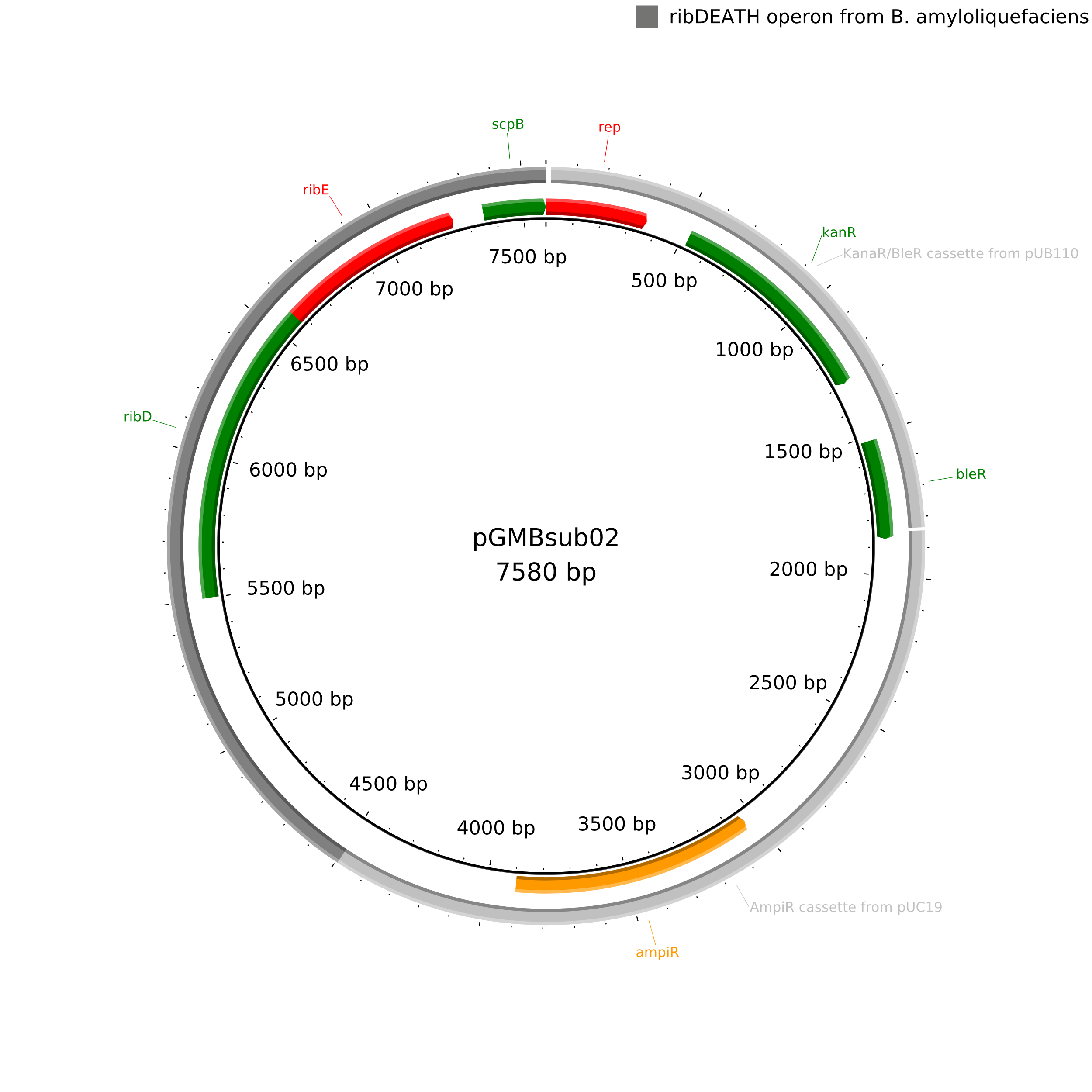
**

**
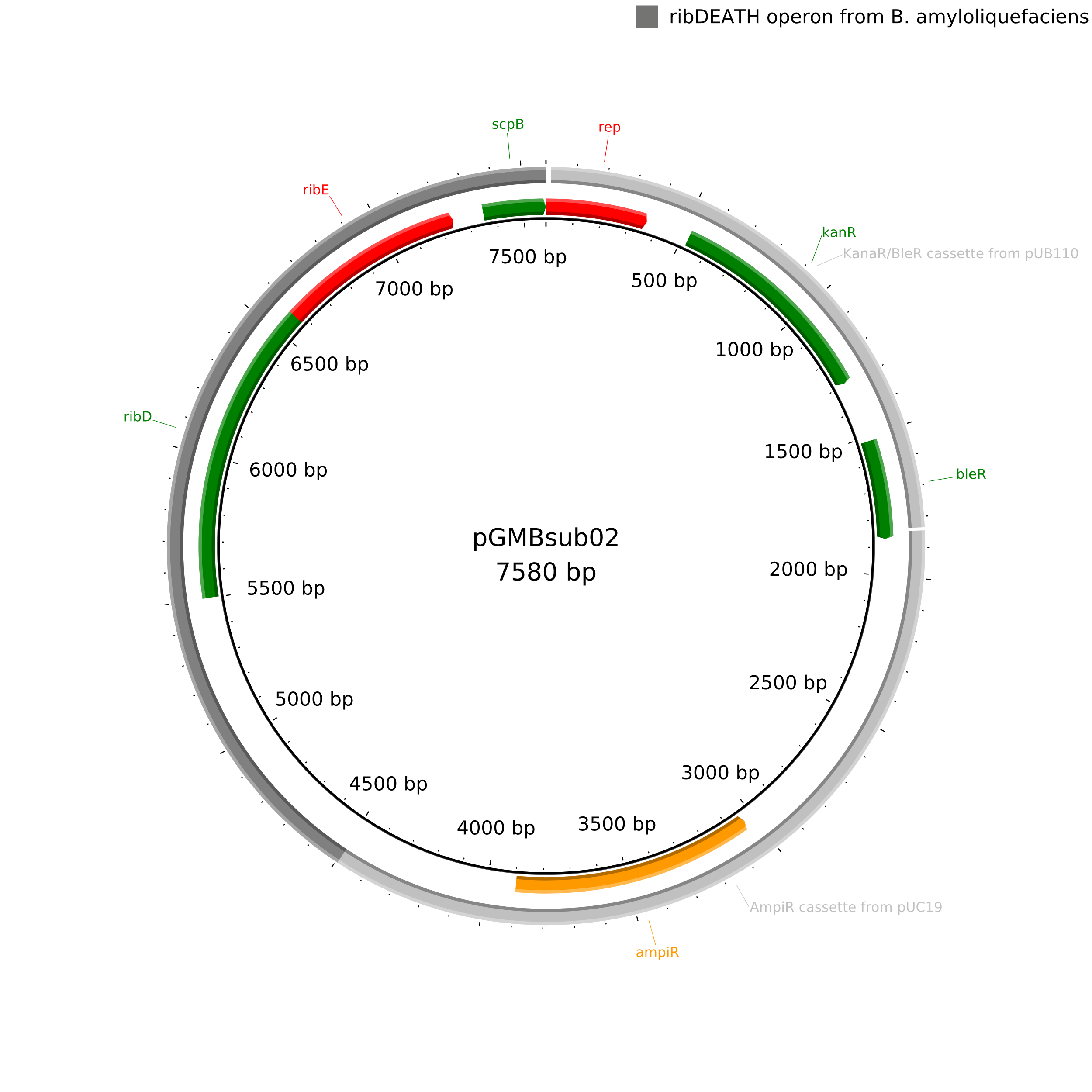
**

**
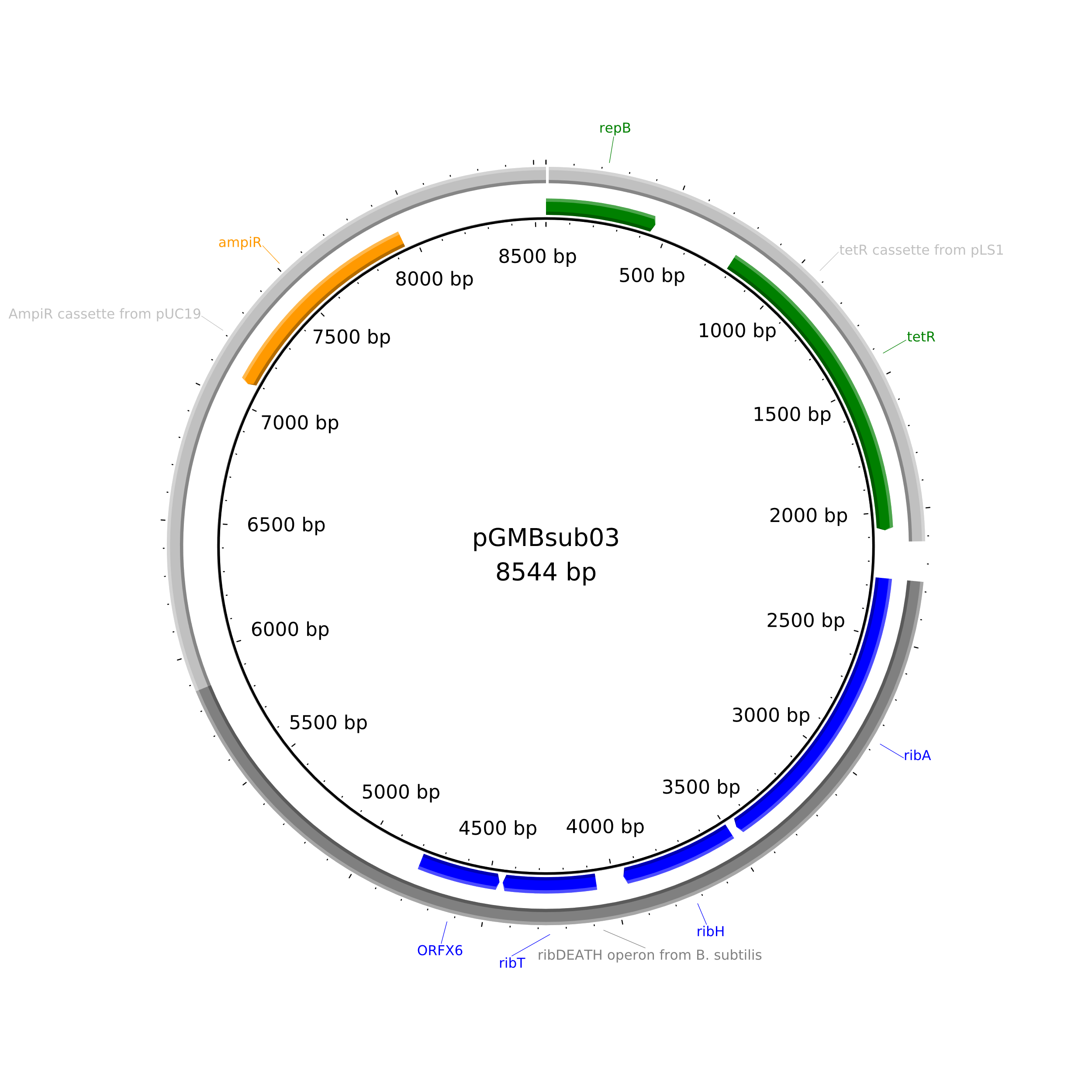
**

**
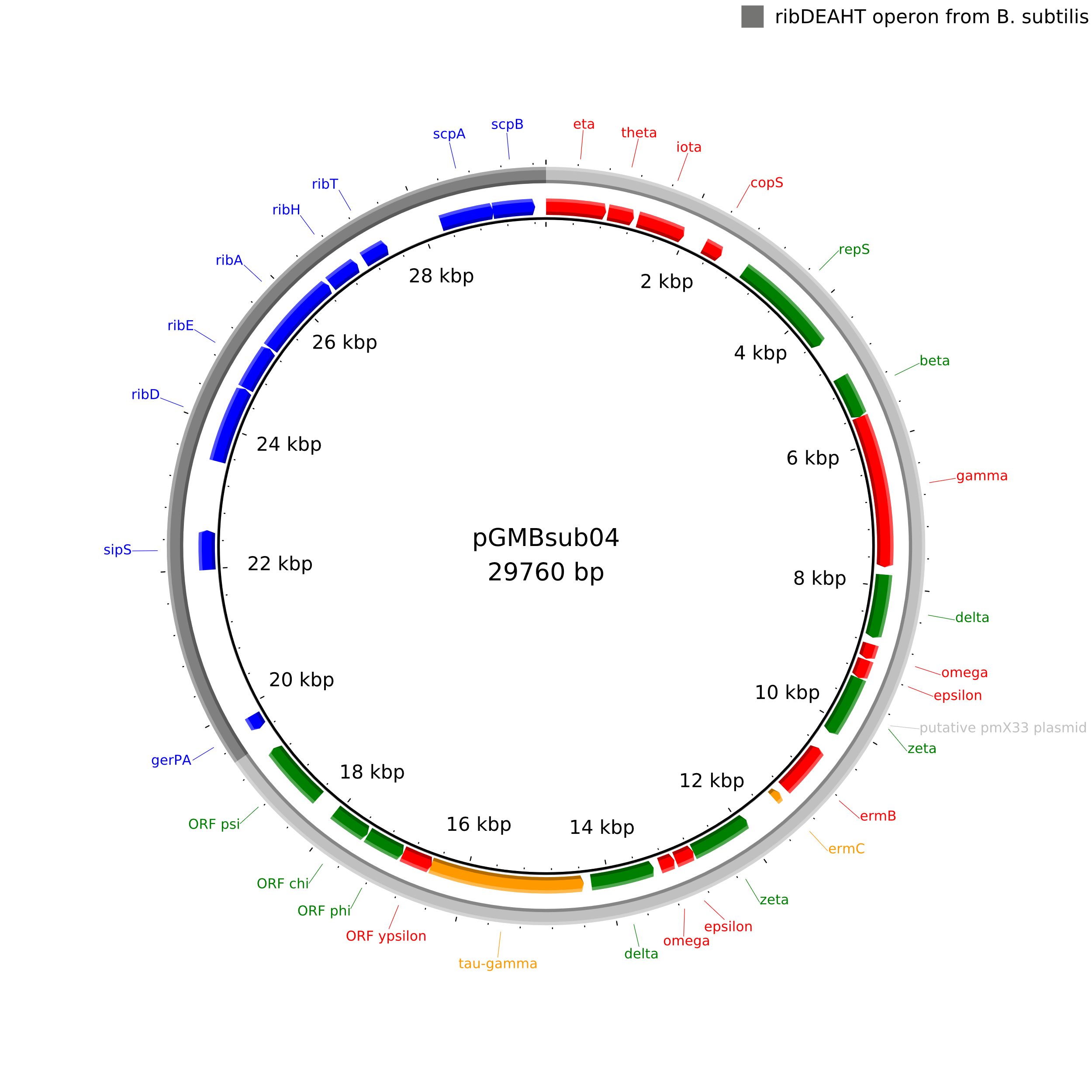
**

**
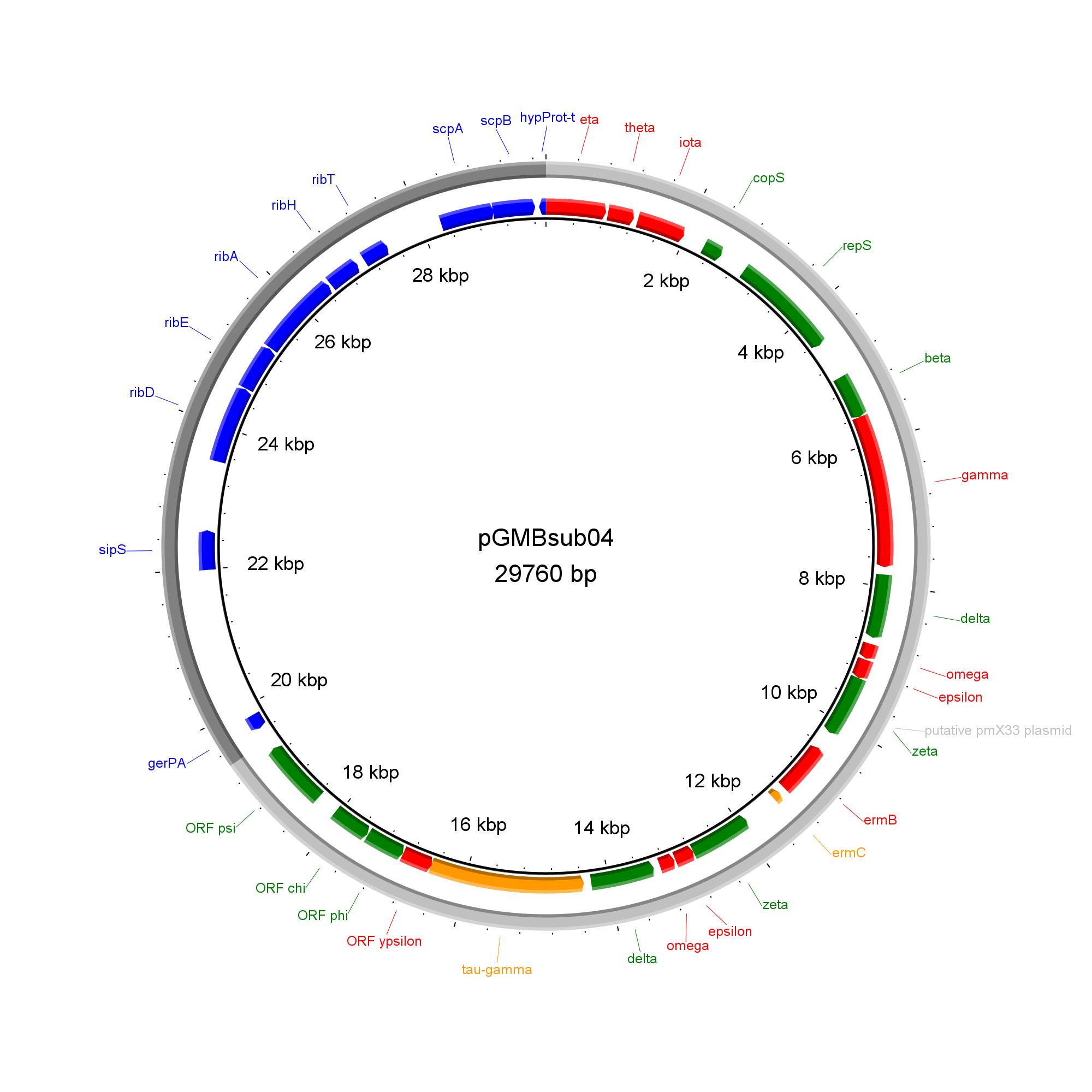
**


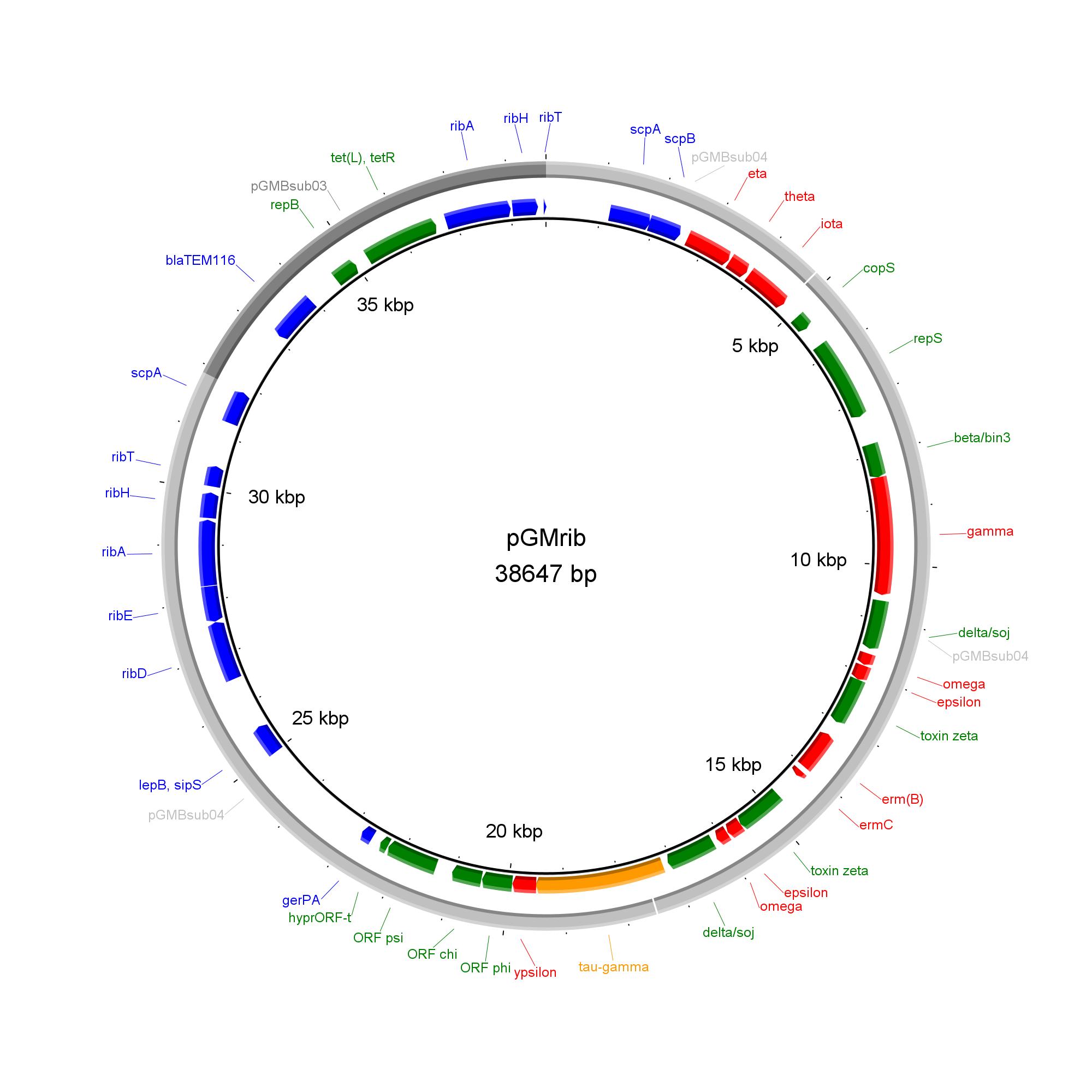


**9.2 Visualization of chromosomal portion in *B. subtilis* GM (10-11)**

**
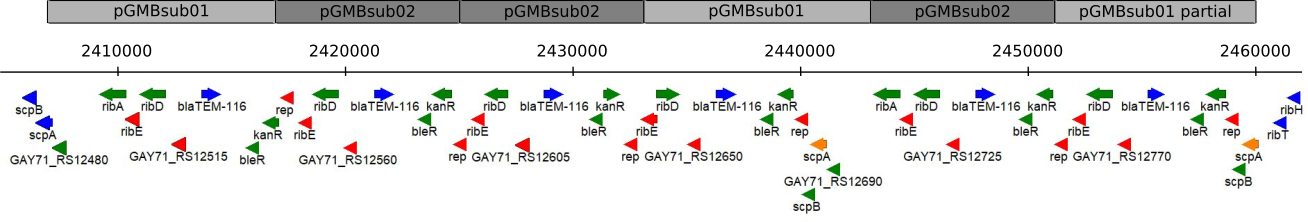
**

**References**

1. Genetic Competence Drives Genome Diversity in Bacillus subtilis [Internet]. [cited 2019 Jul 30]. Available from: https://www.ncbi.nlm.nih.gov/pmc/articles/PMC5765554/

2. Touzain F, Denamur E, Médigue C, Barbe V, El Karoui M, Petit M-A. Small variable segments constitute a major type of diversity of bacterial genomes at the species level. Genome Biol. 2010;11(4):R45.

3. Trifonov V, Rabadan R. Frequency Analysis Techniques for Identification of Viral Genetic Data. mBio. 2010 Aug 31;1(3):e00156-10.

4. Karlin S, Mrázek J, Campbell AM. Codon usages in different gene classes of the Escherichia coli genome. Mol Microbiol. 1998 Sep;29(6):1341–55.

5. Di H, Ye L, Yan H, Meng H, Yamasak S, Shi L. Comparative analysis of CRISPR loci in different Listeria monocytogenes lineages. Biochem Biophys Res Commun. 2014;454(3):399–403.

6. Bosi E, Monk JM, Aziz RK, Fondi M, Nizet V, Palsson BØ. Comparative genome-scale modelling of Staphylococcus aureus strains identifies strain-specific metabolic capabilities linked to pathogenicity. Proc Natl Acad Sci U S A. 2016;113(26):E3801–E3809.

7. Yang T, Zhong J, Zhang J, et al. Pan-Genomic Study of Mycobacterium tuberculosis Reflecting the Primary/Secondary Genes, Generality/Individuality, and the Interconversion Through Copy Number Variations. Front Microbiol. 2018 Aug 17;9:1886.

8. Kavvas ES, Catoiu E, Mih N, et al. Machine learning and structural analysis of Mycobacterium tuberculosis pan-genome identifies genetic signatures of antibiotic resistance. Nat Commun. 2018 Oct 17;9(1):4306.

9. Gill SR, Fouts DE, Archer GL, Mongodin EF, Deboy RT, Ravel J, Paulsen IT, Kolonay JF, Brinkac L, Beanan M, Dodson RJ, Daugherty SC, Madupu R, Angiuoli, SV, Durkin AS, Haft DH, Vamathevan J, Khouri H, Utterback T, Lee C, Dimitrov G, Jiang L, Qin H, Weidman J, Tran K, Kang K, Hance IR, Nelson KE, and Fraser CM. Insights on evolution of virulence and resistance from the complete genome analysis of an early methicillin-resistant Staphylococcus aureus strain and a biofilm-producing methicillin-resistant Staphylococcus epidermidis strain. J Bacteriol. 2005 Apr;187(7):2426-38.

10. Paracchini V, Petrillo M, Reiting R, Angers-Loustau A, Wahler D, Stolz A, et al. Molecular characterization of an unauthorized genetically modified Bacillus subtilis production strain identified in a vitamin B2 feed additive. Food Chem. 2017 Sep 1;230:681–9.

11. Berbers B, Saltykova A, Garcia-Graells C, Philipp P, Arella F, et al. Combining short and long read sequencing to characterize antimicrobial resistance genes on plasmids applied to an unauthorized genetically modified Bacillus. Scientific Reports. 2020 Dec; 10(1):4310.
